# Supplementary figures and images for: The Species-Specific Acquisition and Diversification of a K1-like Family of Killer Toxins in Budding Yeasts of the Saccharomycotina
Source: PLoS Genet. 2021 Feb 4;17(2):e1009341. doi: 10.1371/journal.pgen.1009341 (PMC7888664; doi:10.1371/journal.pgen.1009341)

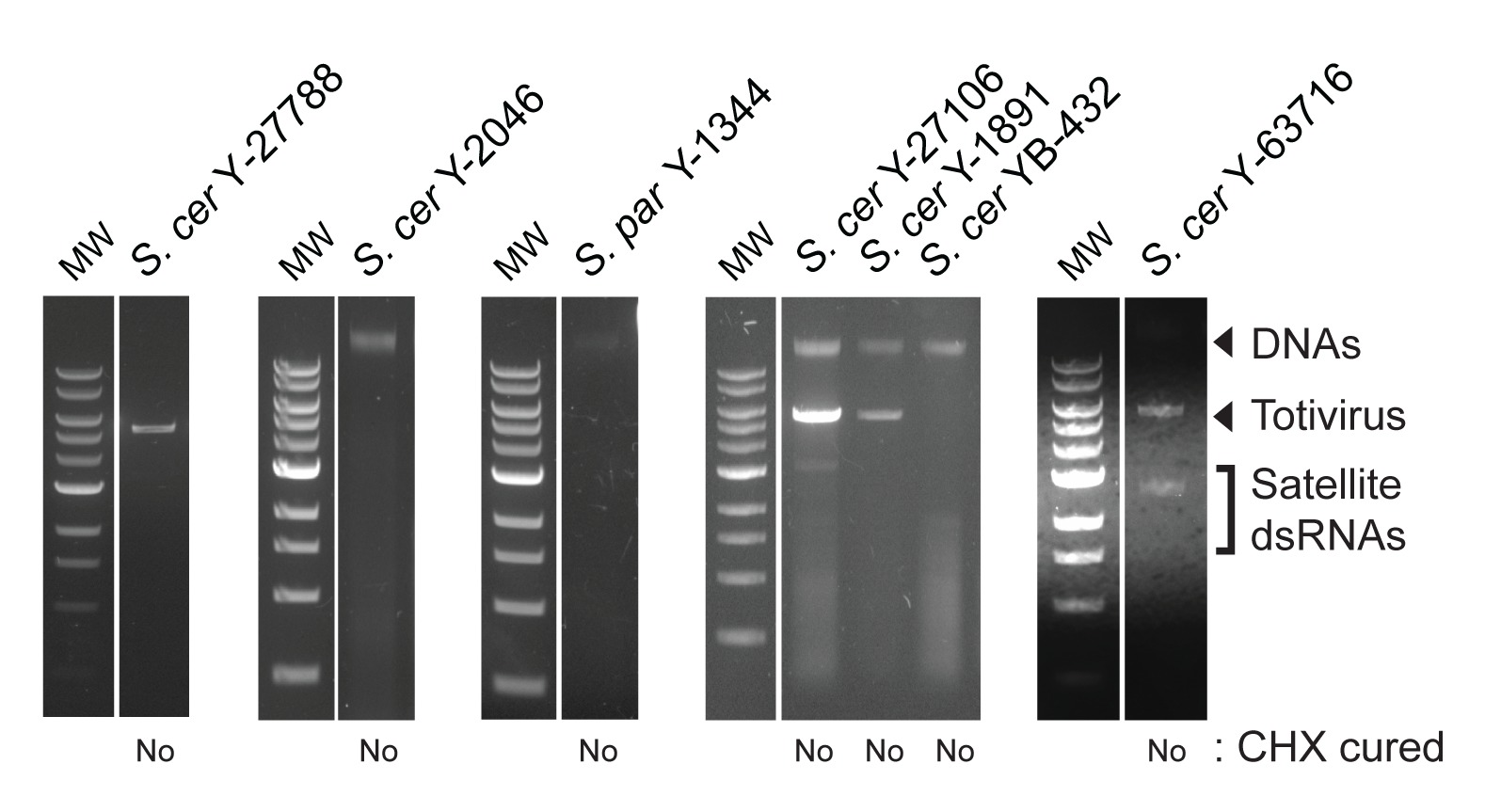

Supplement: S1 Fig — Agarose gel electrophoresis was used to show dsRNAs present in killer yeasts. Satellite dsRNAs are labeled as dsRNAs that are smaller than the associated totivirus dsRNAs. The high molecular weight DNAs are predicted to be mitochondrial in origin. (TIF) [file pgen.1009341.s001.tif]

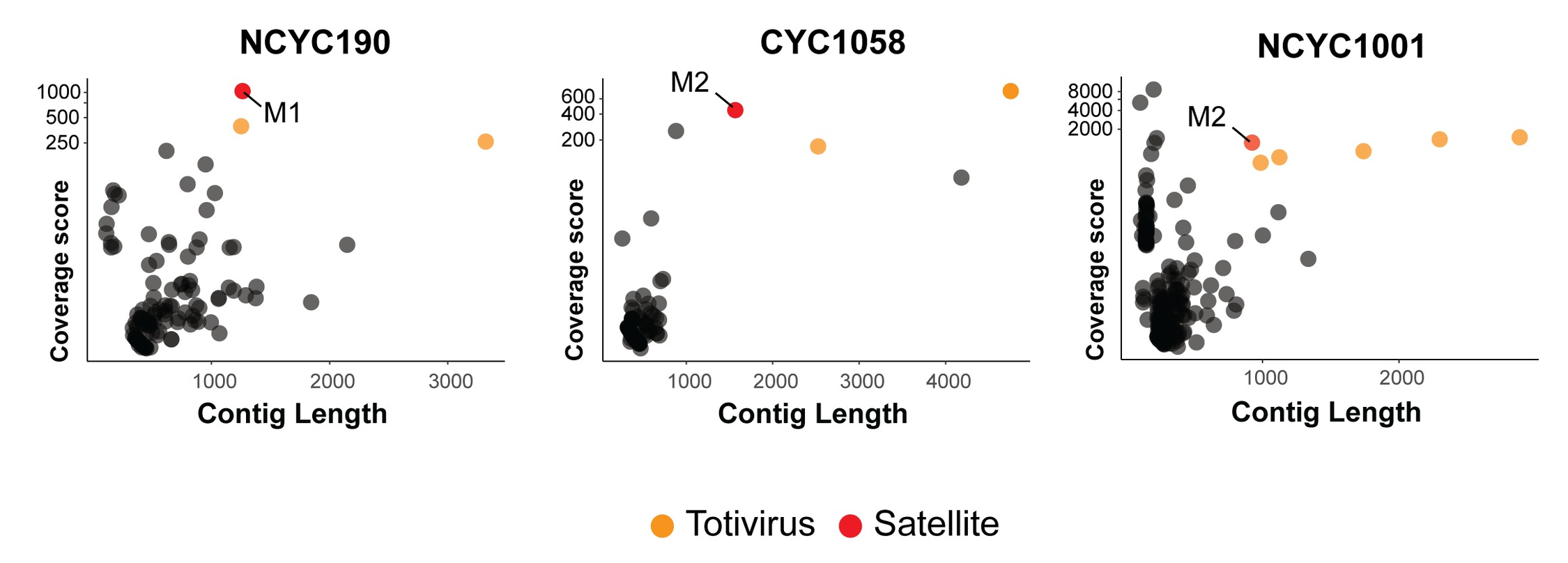

Supplement: S2 Fig — The scatter plots represent all contigs generated after de novo assembly of sequence reads. M satellites are labeled in red according to their relatedness to other previously described sequences. (TIF) [file pgen.1009341.s002.tif]

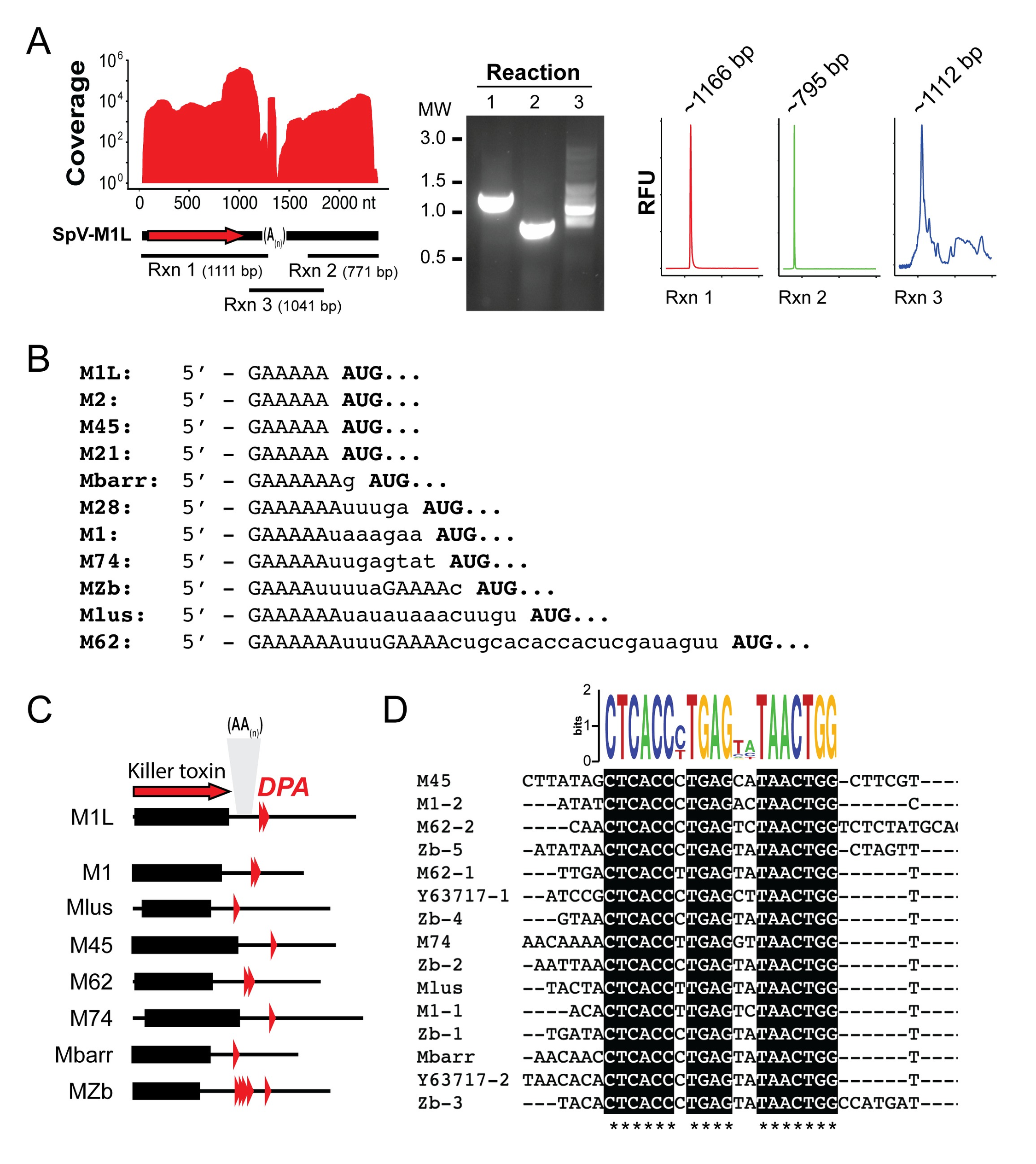

Supplement: S3 Fig — (A) Coverage of the assembled sequence reads for M1L and the positioning of the expected products from three RT-PCR reactions to amplify portions of the K1L ORF (reaction 1), the 3’ UTR (reaction 2), and across the internal poly(A) tract (reaction 3). PCR products shown by agarose gel electrophoresis and their estimated sizes as determined from fragment analysis are represented. (B) The positioning of the repeated DPA element is represented relative to the genomes of eight dsRNA satellites. (C) The consensus sequence derived from 15 DPA elements is shown as a sequence logo and multisequence alignment. (TIF) [file pgen.1009341.s003.tif]

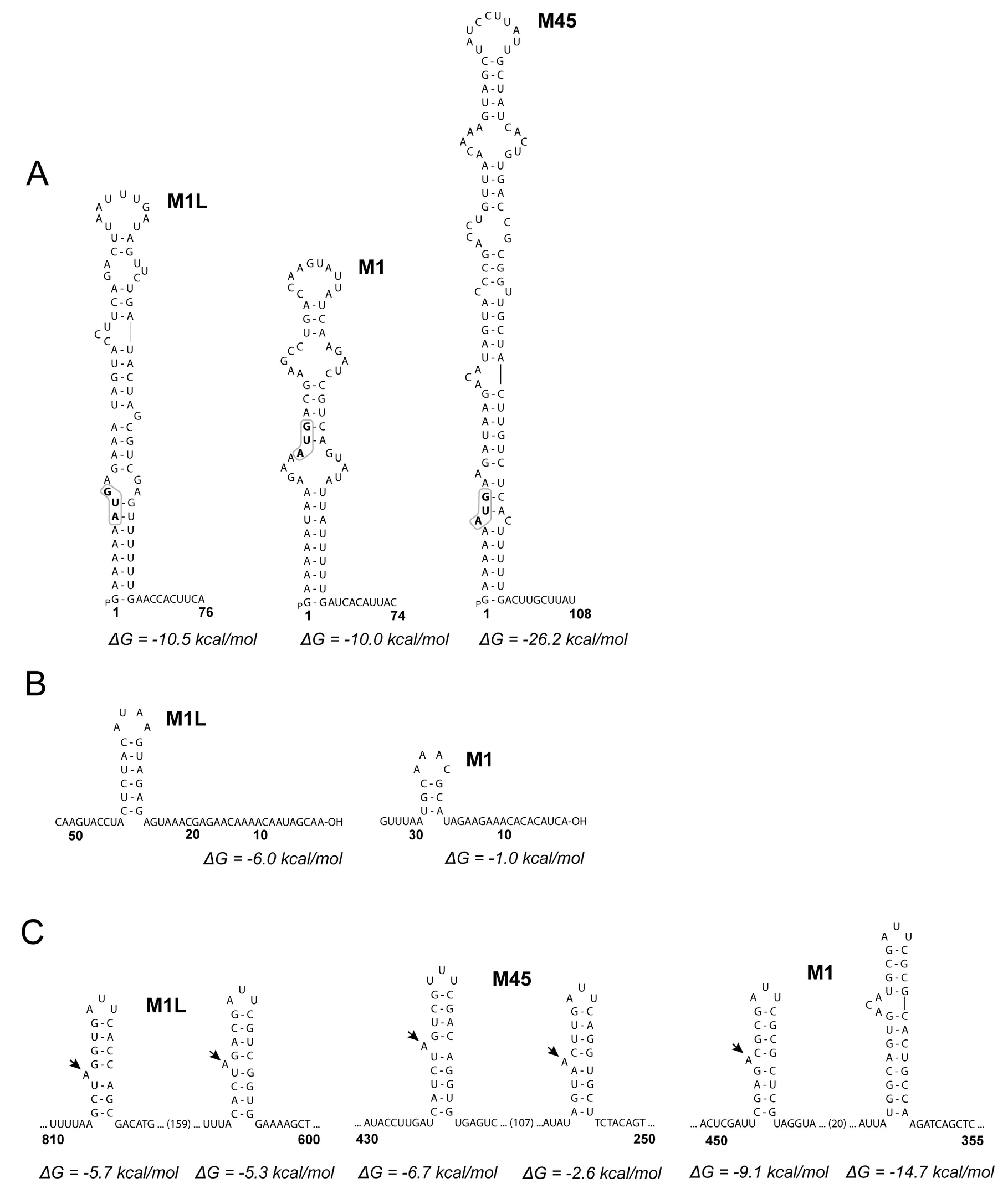

Supplement: S4 Fig — (A) Secondary structure prediction of the 5’ terminal structures. Start codons for the translation of preprotoxin synthesis are highlighted by a grey outline. Numbers represent nucleotides from the 5’ terminus. (B) Putative replication signal represented as a stem-loop at the 3’ end of M1L and M1 satellite. Numbers represent distance from the 3’ terminal nucleotide. (C) Putative viral binding sites (VBS) with a 5’ facing ‘A’ bulge present in the stem-loops (indicated by an arrow). Numbers represent distance from the 3’ terminal nucleotide. The mFold server was used to calculate the change in free energy for each structure [116]. (TIF) [file pgen.1009341.s004.tif]

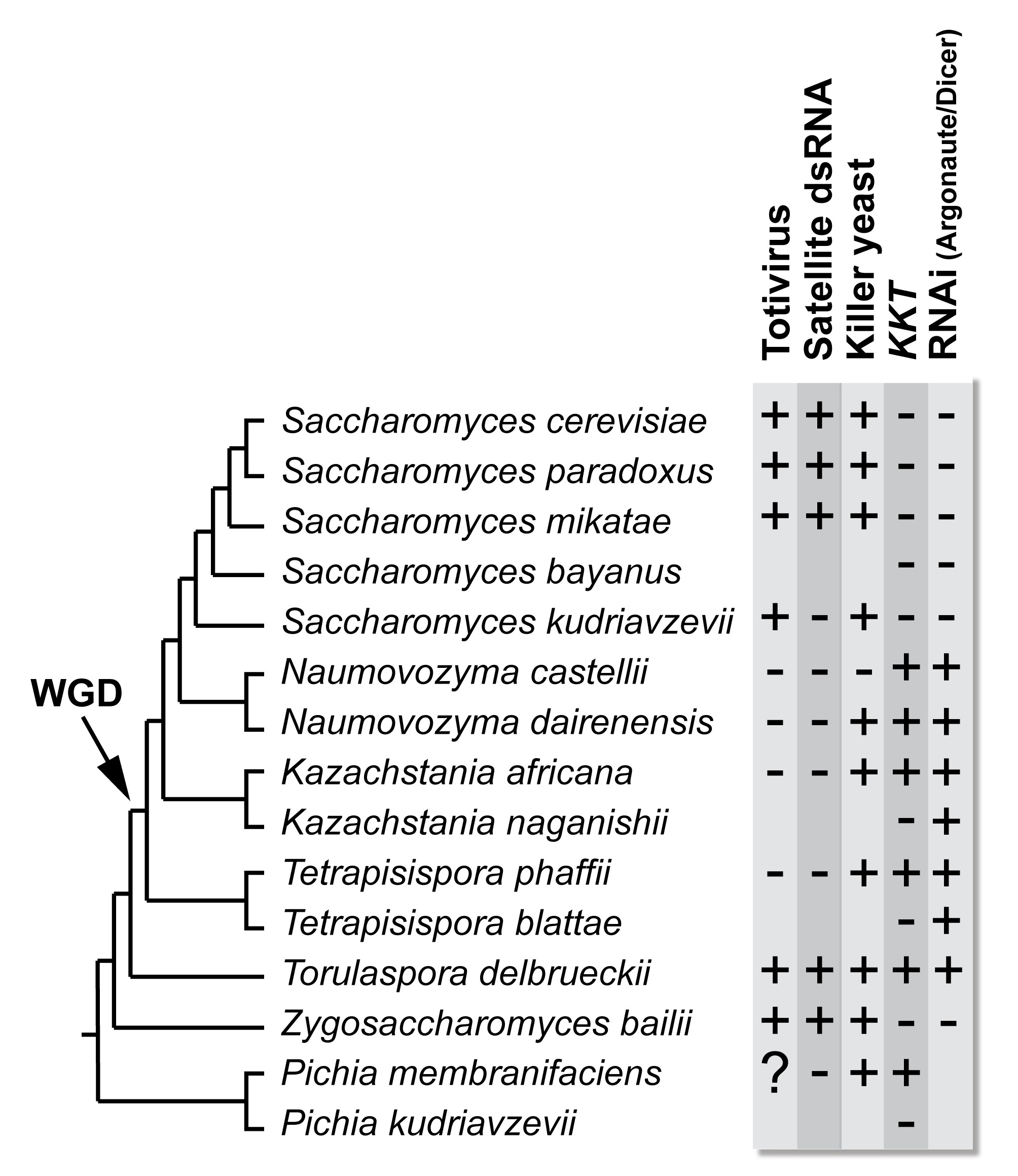

Supplement: S5 Fig — The panel details the presence or absence of totiviruses, satellite dsRNAs, the killer phenotype, KKT genes, and RNAi within 15 species. ‘?’ denotes the uncertainty of the putative dsRNA virus detected in P. membranifaciens in S8 Fig. WGD indicates the ancestral yeast species that underwent a whole genome duplication. (TIF) [file pgen.1009341.s005.tif]

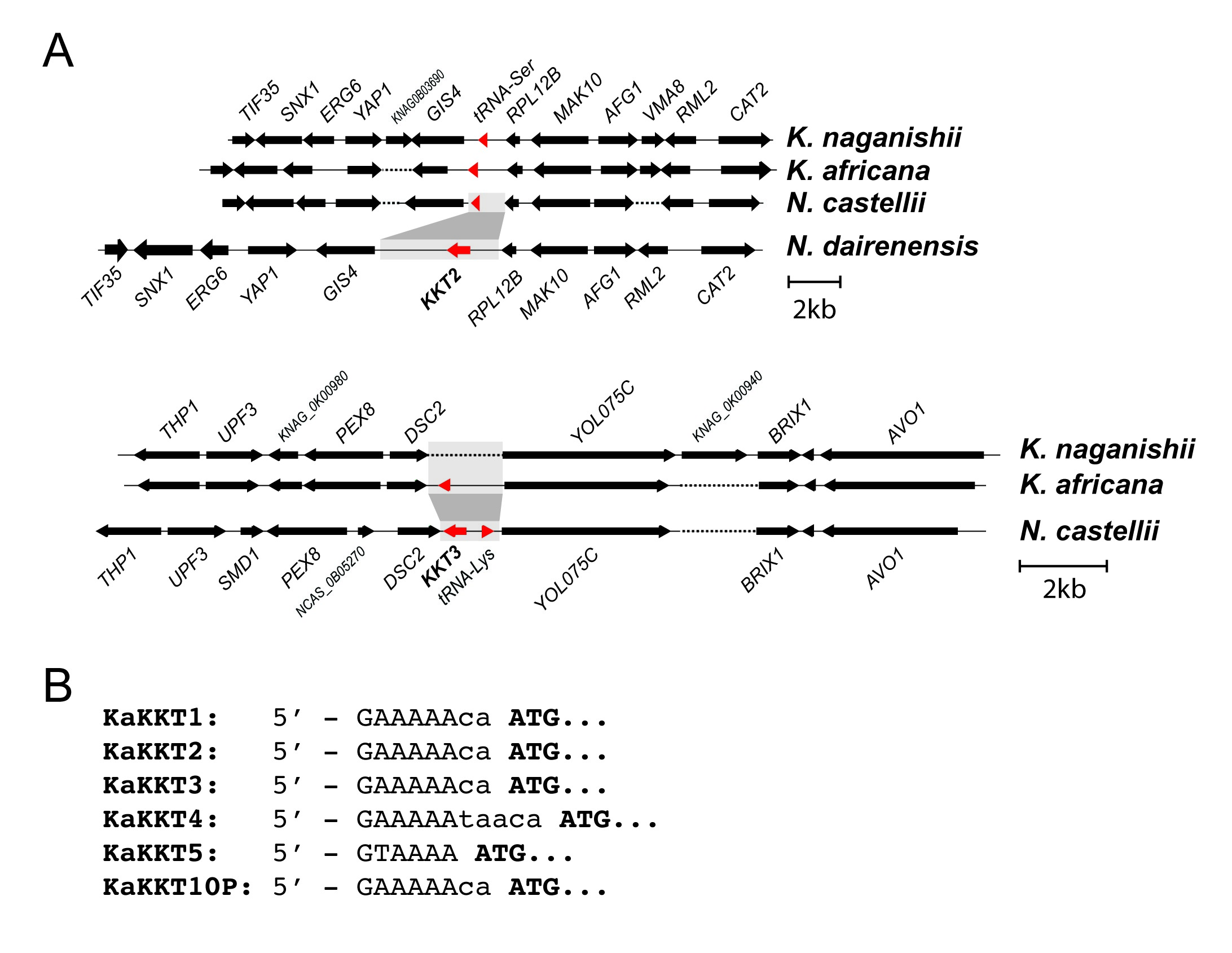

Supplement: S6 Fig — (A) N. dairenensis KKT2 inserted into chromosome III and N. castellii KKT3 inserted into chromosome II. Genes flanking KKT insertions are represented as black arrows and demonstrate synteny between related genomes. Single red triangles represent tRNA genes. Broken lines represent gaps in synteny. (B) 5’ UTR sequence from KKT genes and one pseudogene identified within K. africana. (TIF) [file pgen.1009341.s006.tif]

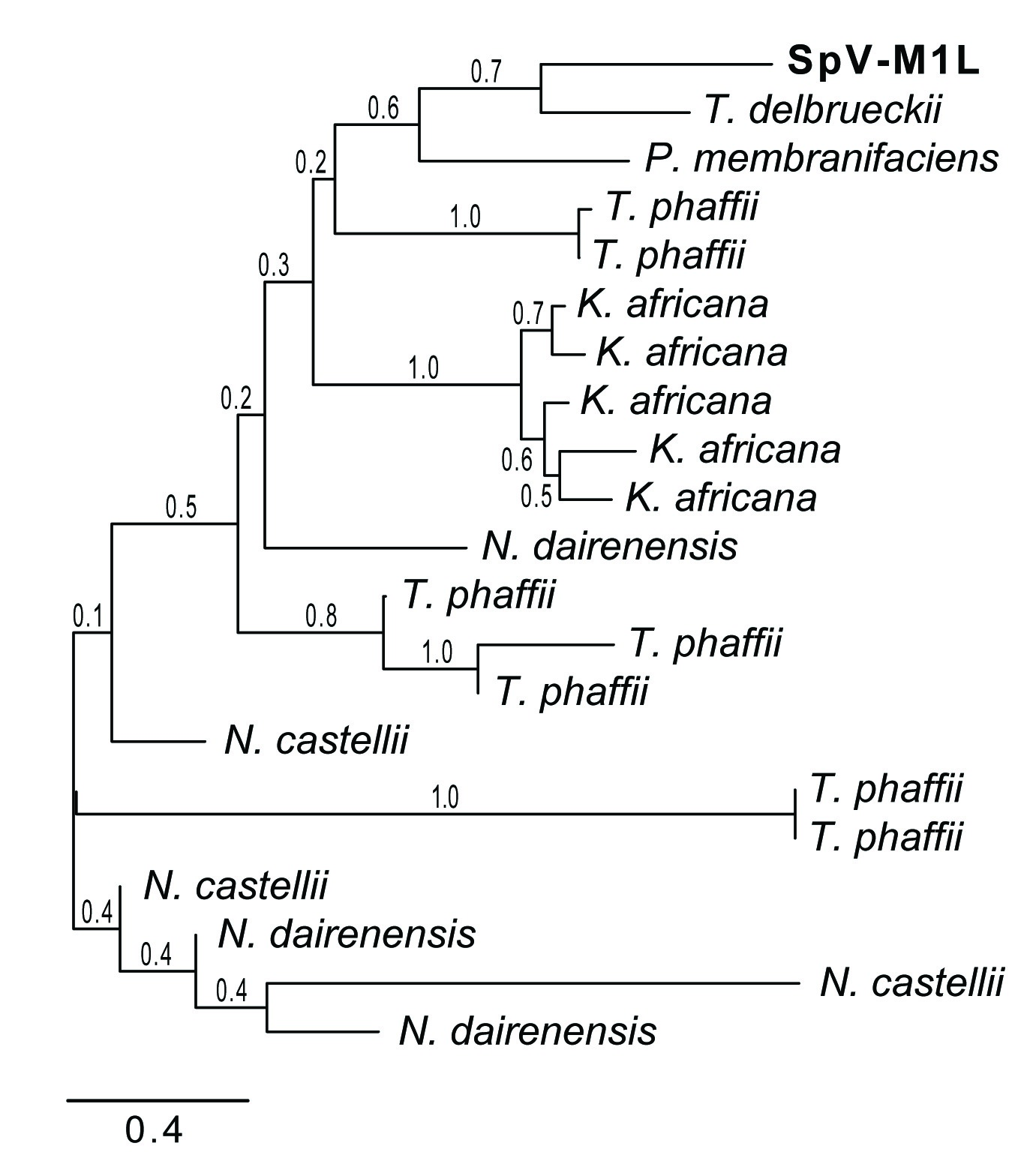

Supplement: S7 Fig — Unrooted neighbor-joining phylogeny of the aligned α-domain of 21 KKT proteins from six species of yeast and K1L from one dsRNA satellite (SpV-M1L). Numerical values represent the bootstrap support for the placement of each node. (TIF) [file pgen.1009341.s007.tif]

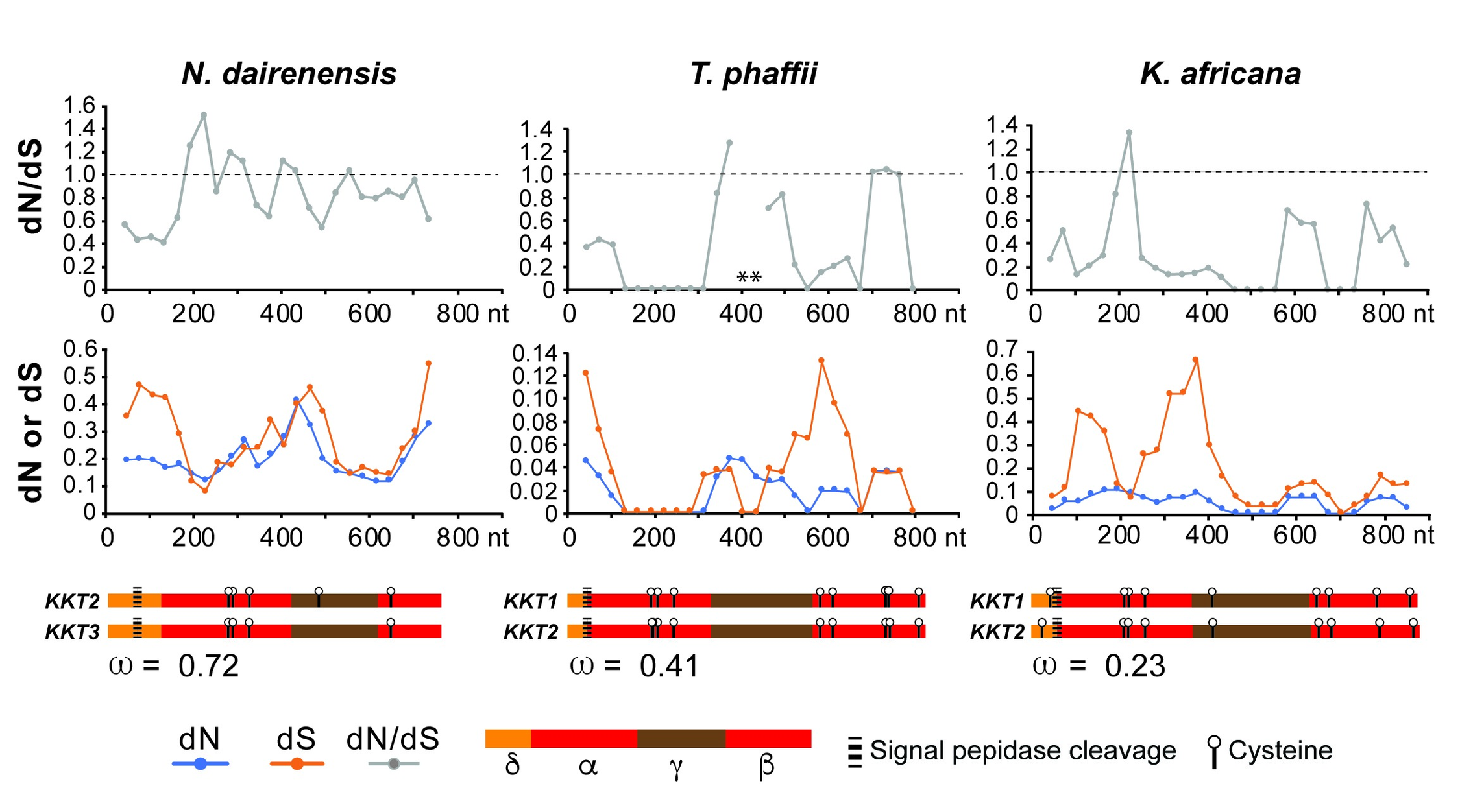

Supplement: S8 Fig — Three sliding window dN/dS calculations are shown for the comparison of three pairs of closely related genes in three yeast species. The x-axis represents the nucleotide (nt) number of each gene and is shown in the context of the predicted domain organization of each pair of genes. Omega values represent the whole gene dN/dS value for each gene pair. Sliding windows analysis was performed using a window of 90 nucleotides with a 30 nucleotide overlap. Asterisks in the T. phaffii dN/dS plot represent instances where dS = 0 and dN > 0. (TIF) [file pgen.1009341.s008.tif]

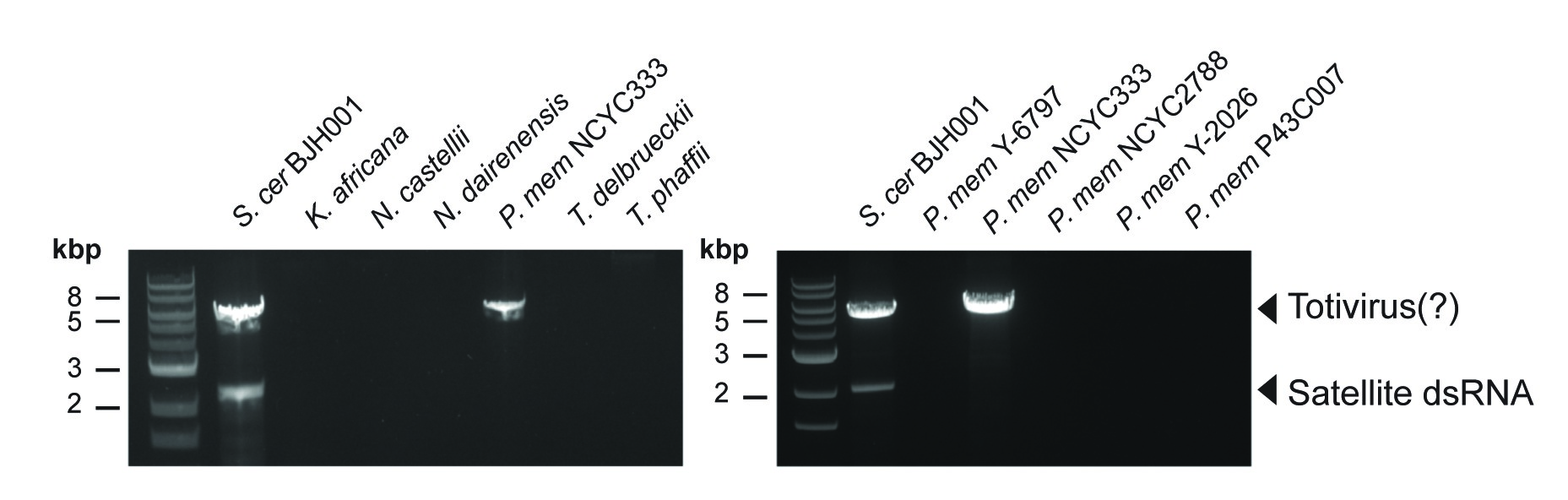

Supplement: S9 Fig — Agarose gel electrophoresis of dsRNAs extracted from different killer yeasts. Stained bands in lane 2 represent a canonical totivirus and satellite dsRNA from S. cerevisiae. (TIF) [file pgen.1009341.s009.tif]

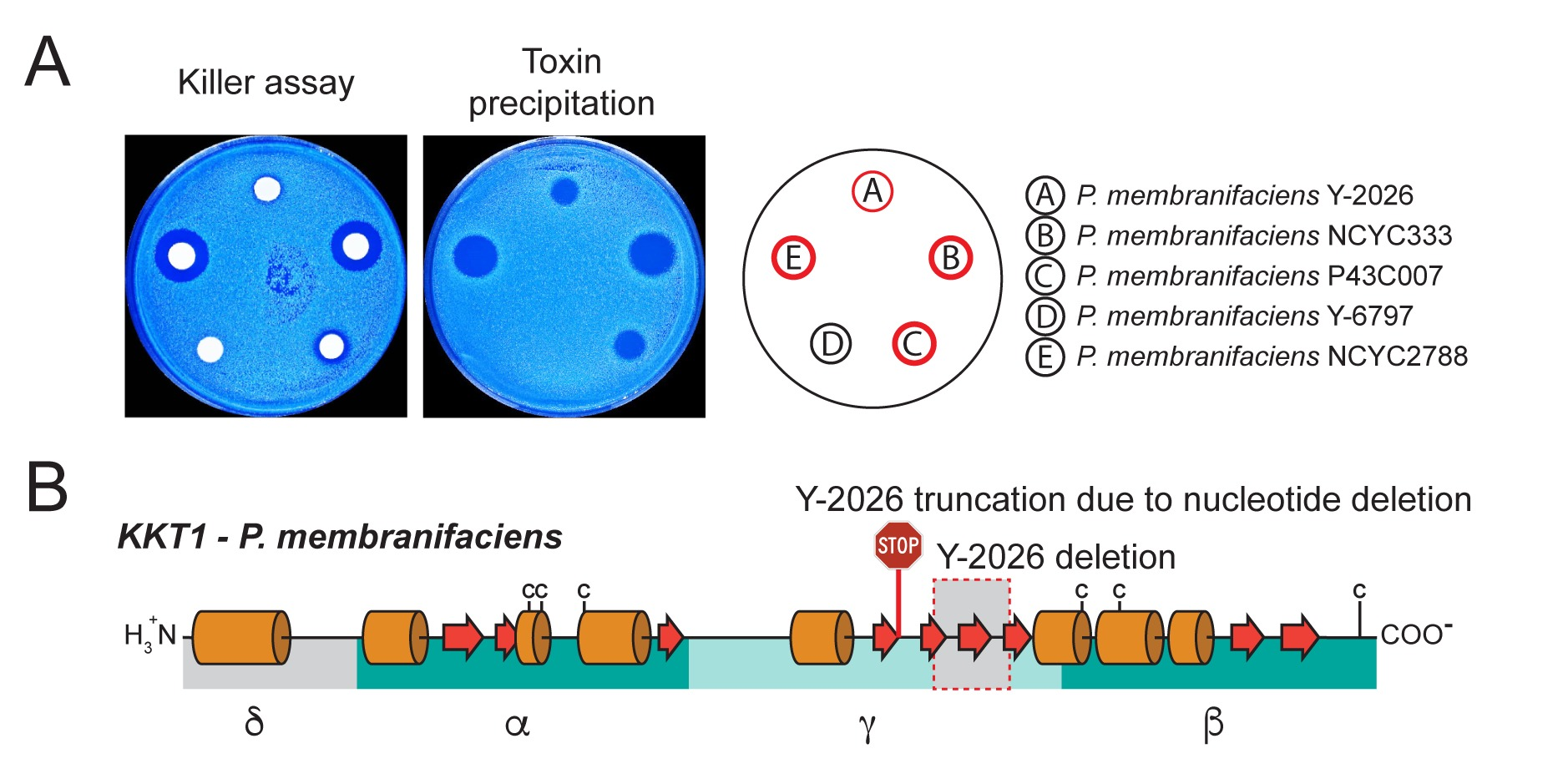

Supplement: S10 Fig — (A) Killer toxin production and partial purification from P. membranifaciens. (B) Mutations within KKT1 in the context of the proteins secondary structure organization (as predicted by Jpred) from strain Y-2026 compared to a full-length active killer toxin sequenced from strain NCYC333. Arrows represent β-sheets and cylinders represent α-helices. “c” represents cysteine residues. (TIF) [file pgen.1009341.s010.tif]

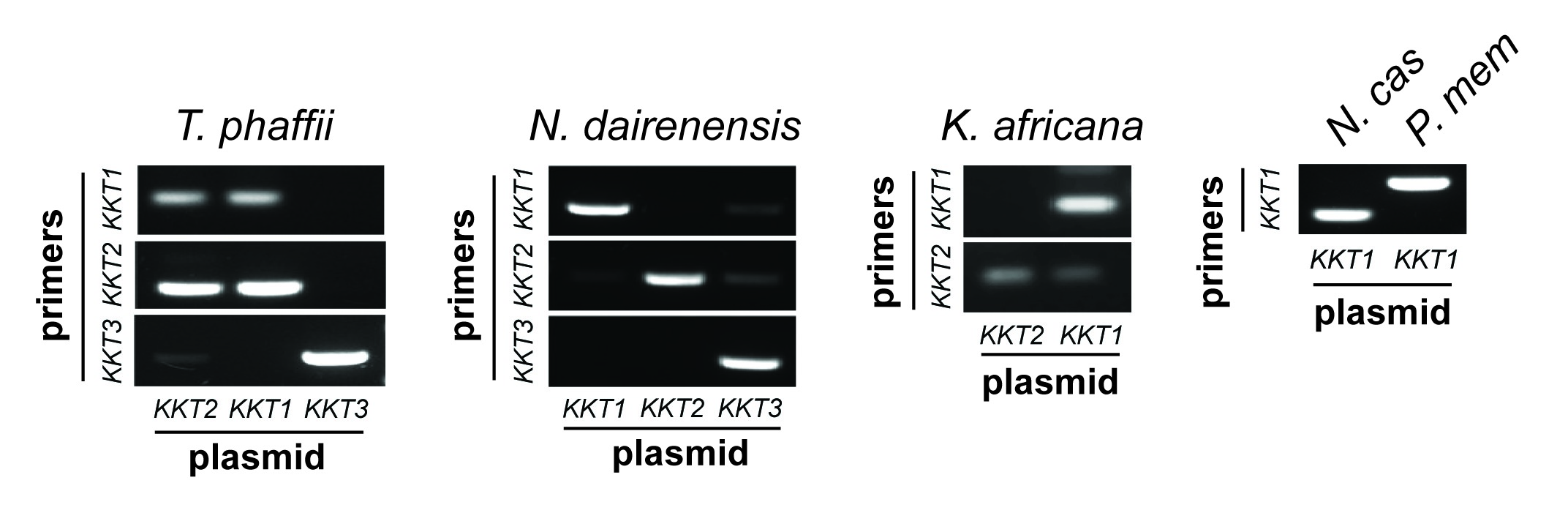

Supplement: S11 Fig — Where possible, primers pairs were designed to recognize different full-length KKT paralogs from T. phaffii, N. dairenensis, and K. africana. DNA plasmids were used as templates to test their specificity. Due the similarities in DNA sequences, specific primer pairs were not identified for TpKKT1, TpKKT2, and KaKKT2. (TIF) [file pgen.1009341.s011.tif]

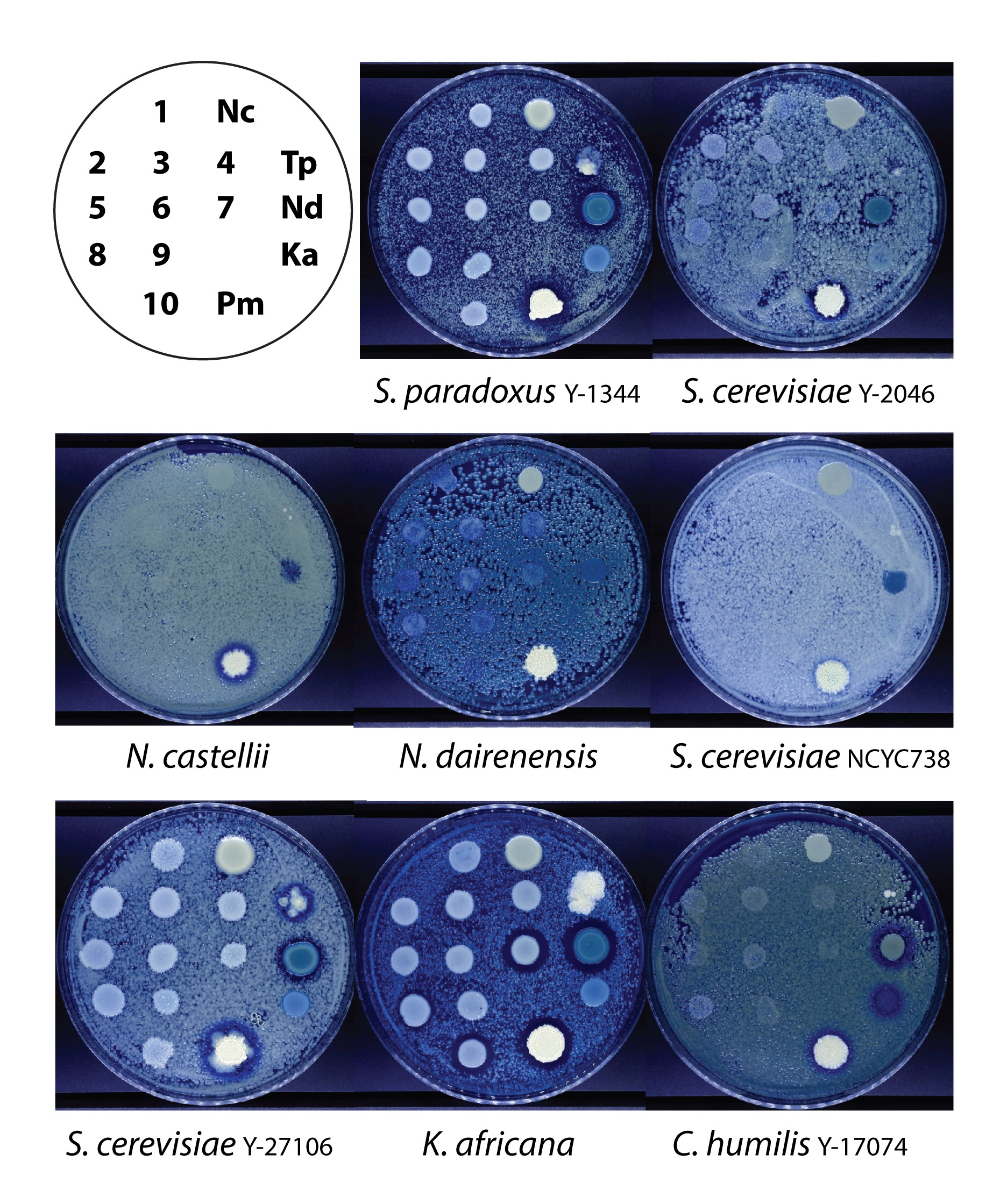

Supplement: S12 Fig — Galactose-dependent ectopic expression of KKT genes from a multicopy plasmid by S. cerevisiae on agar plates seeded with different species of yeasts. Key: 1. pUI114 (NcKKT1), 2. pUI109 (TpKKT2), 3. pUI110 (TpKKT1), 4-pUI111 (TpKKT3), 5. pUI112 (NdKKT3), 6. pUI113 (NdKKT2), 7. pML115 (NdKKT1), 8. pML117 (KaKKT2), 9. pML118 (KaKKT1), 10. pML116 (PmKKT1), Nc. N. castellii NCYC2898, Nd. N. dairenensis NCYC777, Tp. T. phaffii Y-8282, Pm. P. membranifaciens NCYC333. (TIF) [file pgen.1009341.s012.tif]
