# Supplementary material for: The Species-Specific Acquisition and Diversification of a K1-like Family of Killer Toxins in Budding Yeasts of the Saccharomycotina
Source: PLoS Genet. 2021 Feb 4;17(2):e1009341. doi: 10.1371/journal.pgen.1009341 (PMC7888664; doi:10.1371/journal.pgen.1009341)
Supplement: S1 Table — (DOCX) [file pgen.1009341.s013.docx]

| Species | Strain | Killer toxin | Coverage | % ID (nt) K1 | % ID (aa) K1 | % ID (nt) K1 | % ID (aa) K1 |
| --- | --- | --- | --- | --- | --- | --- | --- |
| *S. cerevisiae* | NCYC190 | K1 | 98.5% | 99.7% | 99.4% | n/a | n/a |
|  | CYC1058 | K2 | 92.0% | n/a | n/a | 98.5% | 97.8% |
|  | NCYC1001 | K2 | 86.8% | n/a | n/a | 98.7% | 96.1% |
