# Supplementary material for: The Species-Specific Acquisition and Diversification of a K1-like Family of Killer Toxins in Budding Yeasts of the Saccharomycotina
Source: PLoS Genet. 2021 Feb 4;17(2):e1009341. doi: 10.1371/journal.pgen.1009341 (PMC7888664; doi:10.1371/journal.pgen.1009341)
Supplement: S2 Table — (DOCX) [file pgen.1009341.s014.docx]

| Full-length protein | | | | | | | | **α-domain only** | | | | | | | |
| --- | --- | --- | --- | --- | --- | --- | --- | --- | --- | --- | --- | --- | --- | --- | --- |
| K1L | | | | **K1** | | | | **K1L** | | | | **K1** | | | |
| Name | % ID | % Sim | Name | | % ID | % Sim | Name | | %ID | % Sim | Name | | % ID | % Sim |  |
| KaKKT3 | 27 | 44 | PmKKT1 | | 20 | 35 | TpKKT3 | | 28 | 56 | TpKKT3 | | 25 | 39 |  |
| KaKKT1 | 27 | 45 | TpKKT3 | | 20 | 36 | PmKKT1 | | 28 | 55 | KaKKT3 | | 23 | 42 |  |
| KaKKT2 | 27 | 45 | NdKKT1 | | 19 | 34 | KaKKT1 | | 27 | 53 | PmKKT1 | | 22 | 41 |  |
| TpKKT3 | 25 | 44 | KaKKT3 | | 19 | 33 | KaKKT2 | | 26 | 54 | **K1L** | | **22** | **35** |  |
| NdKKT1 | 24 | 41 | KaKKT1 | | 19 | 32 | KaKKT3 | | 25 | 49 | NdKKT1 | | 21 | 39 |  |
| PmKKT1 | 21 | 36 | KaKKT2 | | 19 | 32 | NdKKT1 | | 24 | 51 | KaKKT2 | | 21 | 39 |  |
| TpKKT2 | 18 | 31 | **K1L** | | **18** | **32** | NdKKT2 | | 23 | 43 | KaKKT1 | | 20 | 38 |  |
| K1 | **18** | **32** | TpKKT1 | | 13 | 28 | **K1** | | **22** | **35** | NcKKT1 | | 17 | 35 |  |
| TpKKT1 | 18 | 30 | TpKKT2 | | 13 | 28 | NcKKT1 | | 22 | 46 | NdKKT2 | | 17 | 34 |  |
| NcKKT1 | 11 | 27 | NcKKT1 | | 11 | 27 | NdKKT3 | | 20 | 41 | NdKKT3 | | 15 | 30 |  |
| NdKKT2 | 10 | 24 | NdKKT2 | | 9 | 22 | TpKKT1 | | 16 | 33 | TpKKT1 | | 13 | 30 |  |
| NdKKT3 | 9 | 22 | NdKKT3 | | 8 | 21 | TpKKT2 | | 16 | 33 | TpKKT2 | | 13 | 30 |  |
