# Supplementary material for: The Species-Specific Acquisition and Diversification of a K1-like Family of Killer Toxins in Budding Yeasts of the Saccharomycotina
Source: PLoS Genet. 2021 Feb 4;17(2):e1009341. doi: 10.1371/journal.pgen.1009341 (PMC7888664; doi:10.1371/journal.pgen.1009341)
Supplement: S3 Table — (DOCX) [file pgen.1009341.s015.docx]

| Species | Strain | Gene | Protein | Chr | Position | nt | aa |
| --- | --- | --- | --- | --- | --- | --- | --- |
| *K. africana* | CBS 2517 | KaKKT1 | XP_003956791.1 | 4 | 4933-5865 | 932 | 310 |
|  |  | KaKKT3 | XP_003959753.1 | 12 | 3868-4800 | 932 | 310 |
|  |  | KaKKT2 | XP_003954584.1 | 1 | 14695-15627 | 932 | 310 |
|  |  | KaKKT5 | XP_003956787.1 | 3 | 1322627-1323511 | 884 | 153 |
|  |  | KaKKT4 | XP_003959210.1 | 9 | 592072-591575 | 885 | 165 |
|  |  | KaKKT6 | XP_003957738.1 | 6 | 4855-5505 | 650 | 216 |
|  |  | KaKKT7P | - | 8 | 738788-737853 | 935 | - |
|  |  | KaKKT8P | - | 3 | 1320455-1320080 | 375 | - |
|  |  | KaKKT9P | - | 11 | 29171-30101 | 930 | 95 |
| *N. castellii* | CBS 4309 | NcKKT1 | XP_003677247.1 | 6 | 820175-821086 | 911 | 303 |
|  |  | NcKKT2 | XP_003677251.1 | 7 | 4472-5032 | 560 | 186 |
|  |  | NcKKT3 | XP_003674985.1 | 2 | 979503-979970 | 467 | 155 |
| *N. dairenensis* | CBS 421 | NdKKT1 | XP_003671029.2 | 7 | 263-1186 | 923 | 307 |
|  |  | NdKKT2 | XP_003669012.1 | 3 | 214531-215346 | 815 | 271 |
|  |  | NdKKT3 | XP_003671820.1 | 8 | 997352-998167 | 815 | 271 |
|  |  | NdKKT4P | - | 5 | 1179525-1178607 | 918 | - |
|  |  | NdKKT5P | - | 6 | 1136398-1135718 | 680 | - |
|  |  | NdKKT7P | - | 6 | 1135727-1135598 | 129 | - |
| *T. phaffii* | CBS 4417 | TpKKT3 | XP_003683531.1 | 1 | 8390-9322 | 932 | 310 |
|  |  | TpKKT1 | XP_003687580.1 | 11 | 11192-10317 | 875 | 291 |
|  |  | TpKKT5 | XP_003684118.1 | 2 | 7999-8806 | 807 | 161 |
|  |  | TpKKT6 | XP_003684596.1 | 2 | 1153004-1153633 | 629 | 209 |
|  |  | TpKKT7 | XP_003686950.1 | 8 | 749558-750160 | 602 | 200 |
|  |  | TpKKT2 | XP_003684117.1 | 2 | 1052-1927 | 875 | 291 |
|  |  | TpKKT4 | XP_003685085.1 | 3 | 1089707-1090513 | 806 | 268 |
|  |  | TpKKT8 | XP_003686288.1 | 7 | 6094-5576 | 518 | 172 |
|  |  | TpKKT12P | - | 11 | 512438-515245 | 807 | - |
|  |  | TpKKT9P | - | 11 | 521694-523935 | 241 | - |
|  |  | TpKKT10P | - | 11 | 518825-521023 | 198 | - |
|  |  | TpKKT11P | - | 13 | 451100-453480 | 380 | - |
|  |  | TpKKT13P | - | 11 | 520515-520628 | 113 | - |
|  |  | TpKKT13P | - | 11 | 520515-520628 | 113 | - |
|  |  | TpKKT14P | - | 7 | 3752-3575 | 177 | - |
| *T. delbrueckii* | CBS 1146 | TdKKT1 | XP_003680807.1 | 4 | 11668-12189 | 522 | 173 |
| *P. membranifaciens* | KS47-1 | PmKKT1 | GAV30688.1 | n/a | c2248-1259 | 990 | 390 |
