# Supplementary material for: The Species-Specific Acquisition and Diversification of a K1-like Family of Killer Toxins in Budding Yeasts of the Saccharomycotina
Source: PLoS Genet. 2021 Feb 4;17(2):e1009341. doi: 10.1371/journal.pgen.1009341 (PMC7888664; doi:10.1371/journal.pgen.1009341)

File S1. Image data of killer phenotypes exhibited by strains of *Saccharomyces* yeasts as summarized in figure 1. The organization of a large collection of strains of *Saccharomyces* yeasts arrayed on killer assay agar plates. Each killer yeast was assayed for toxin activity against 8 different lawns of susceptible yeast strains. Black squares indicate detectable killer toxin production.

		Sensitive Lawns							
		K12	FY4	BY4741	UWOPS 83-787.3	CBS 432	CBS7001	SSS 104	NCYC 2729
Yeasts Tested for Killer Phenotype	Y-580								
	Y-670								■
	Y-846								
	Y-969								
	Y-972								
	Y-1374								
	Y-11845								
	Y-12624								
	Y-12646								
	Y-12648								
	Y-17034								
	Y-27339								
	Y-27470								
	Y-48770								
	Y-63707								
	Y-63718								
	YB-254								
	YB-432								■
	Y-567								
	Y-851								
	Y-852								
	Y-897								
	Y-898								
	YB-908								■
	Y-954								
	Y-975								
	Y-976								
	Y-977								■
	Y-1018								
	Y-1089								
	Y-1285								
	Y-1301								
	Y-1370								
	Y-1428								
	Y-1429								
	Y-1430								
	Y-1436								
	Y-1438			■					■
	Y-1536								
	Y-1540								
	YB-1773								
	Y-1891			■					■
	Y-2044								
	Y-2045								
	Y-2046			■					

	Y-2204								
	Y-2205								
	Y-2429	■		■					■
	Y-2430								
	Y-2432								
	Y-2434								
	YB-4237			■					
	YB-4255								
	YB-4634								
	YB-4635								
	Y-5508								
	Y-5509	■		■					
	Y-5510								
	Y-7327								
	Y-7328								
	Y-7567								
	Y-10988								
	Y-11875								
	Y-12842								
	Y-17009								■
	Y-17898			■					
	Y-27105	■							
	Y-27106							■	
	Y-27437								
	Y-27788			■					
	Y-27796								
	y-63703								
	Y-63748								
	Y-63749								
	Y-27340								
	Y-27341								
	Y-27342			■					
	Y-27471								
	Y-63704			■					
	Y-63705								
	Y-63706								
	Y-788								
	Y-863								
	Y-911								
	Y-1088	■		■				■	
	Y-1344			■					
	Y-1356								
	Y-1548								
	Y-1912								
	Y-2038								
	YB-2047								
	YB-4137								
	YB-4565			■					
	Y-5688								
	Y-6177								
	Y-6179								
	Y-11842								
	Y-12602			■					
	Y-17218								
	Y-17353								
	Y-63708								
	Y-63709								
	Y-63710								
	Y-63711			■				■	
	Y-63712								

	Y-63713								
	Y-63714								
	Y-63715			■				■	■
	Y-63716	■							■
	Y-63717			■					■

Key

<table>
<tr><td colspan="6">Y-1436</td></tr>
<tr><td colspan="3">Y-2045</td><td colspan="3">Y-1438</td></tr>
<tr><td colspan="2">Y-2044</td><td colspan="2">Y-2046</td><td colspan="2">Y-1536</td></tr>
<tr><td colspan="3">Y-1891</td><td colspan="3">Y-1540</td></tr>
<tr><td colspan="6">YB-1773</td></tr>
</table>

BY4741

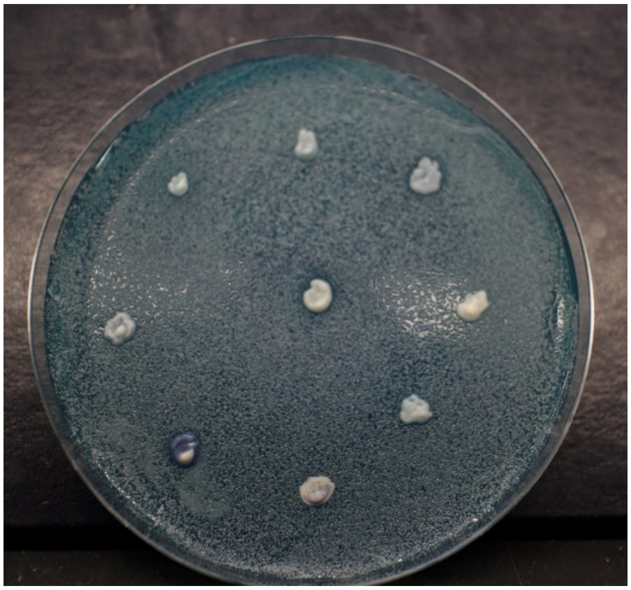

NCYC 2729

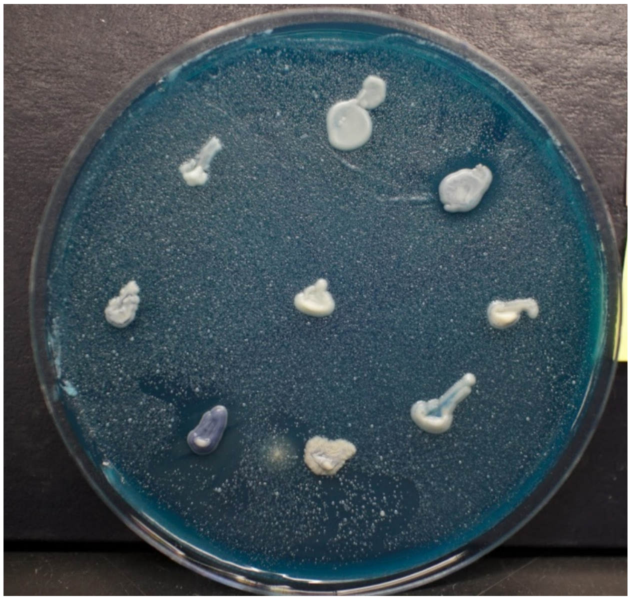

Key

<table>
<tr><td colspan="6">Y-2204</td></tr>
<tr><td colspan="3">YB-4255</td><td colspan="3">Y-2205</td></tr>
<tr><td colspan="2">YB-4237</td><td colspan="2">YB-4634</td><td colspan="2">Y-2429</td></tr>
<tr><td colspan="3">Y-2434</td><td colspan="3">Y-2430</td></tr>
<tr><td colspan="6">Y-2432</td></tr>
</table>

K 12

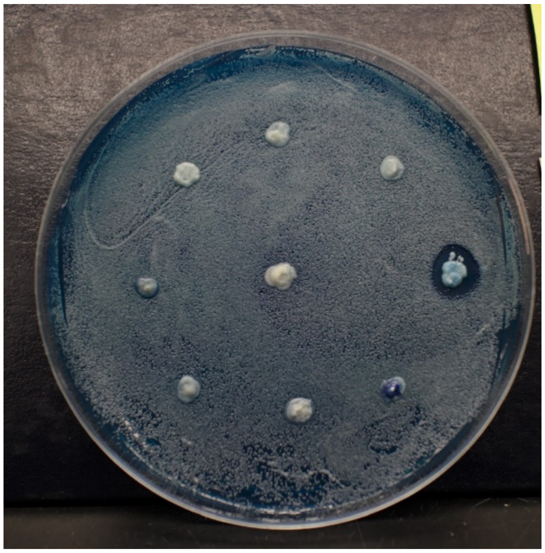

BY4741

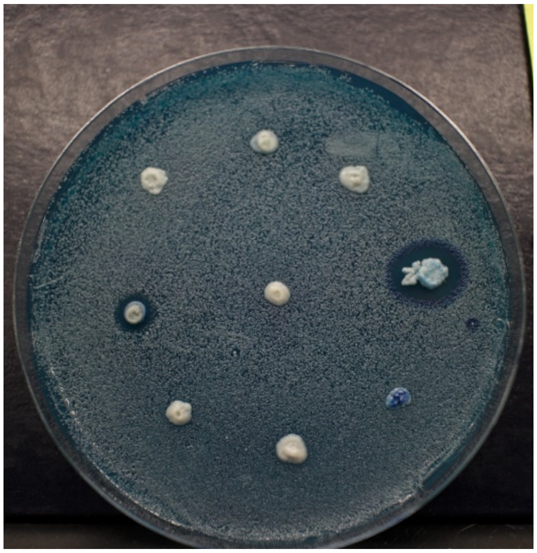

SSS 104

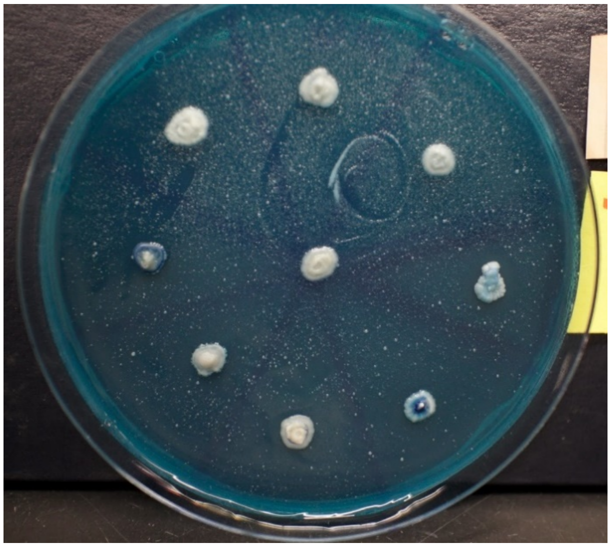

NCYC 2729

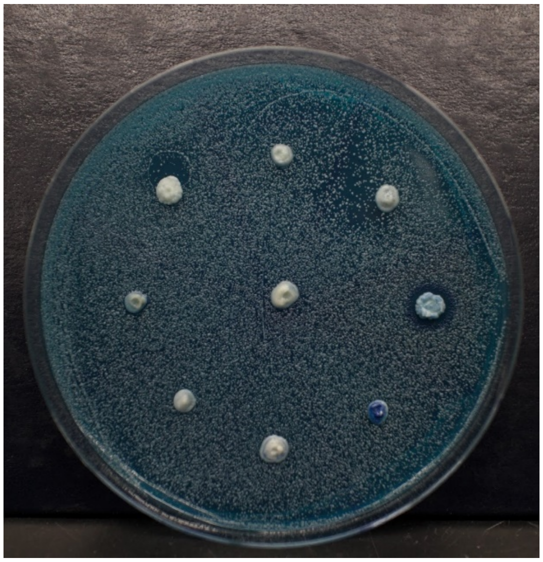

Key

<table>
<tr><td colspan="6">YB-4635</td></tr>
<tr><td colspan="3">Y-10988</td><td colspan="3">Y-5508</td></tr>
<tr><td colspan="2">Y-7567</td><td colspan="2">Y-11875</td><td colspan="2">Y-5509</td></tr>
<tr><td colspan="3">Y-7328</td><td colspan="3">Y-5510</td></tr>
<tr><td colspan="6">Y-7327</td></tr>
</table>

K12

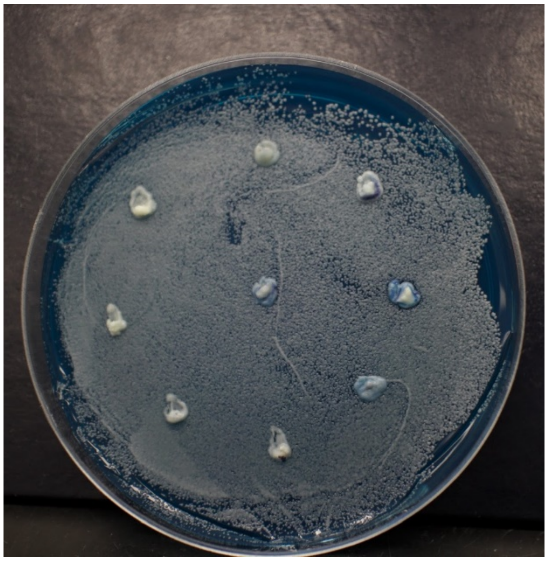

BY4741

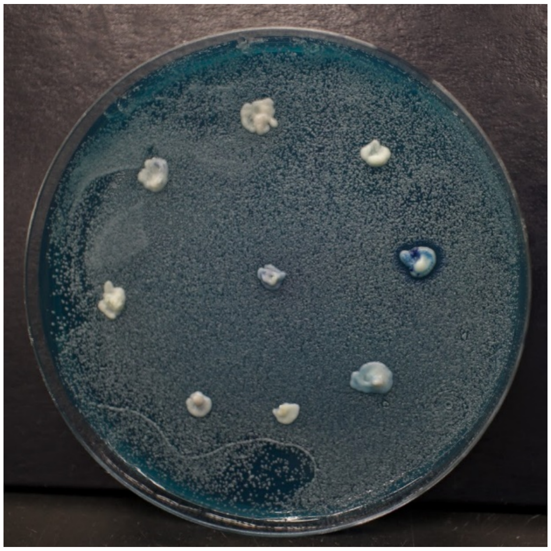

Key

<table>
<tr><td colspan="6">Y-12842</td></tr>
<tr><td colspan="3">Y-27796</td><td colspan="3">Y-17009</td></tr>
<tr><td colspan="2">Y-27788</td><td colspan="2">Y-63703</td><td colspan="2">Y-17898</td></tr>
<tr><td colspan="3">Y-27437</td><td colspan="3">Y-27105</td></tr>
<tr><td colspan="6">Y-27106</td></tr>
</table>

K12

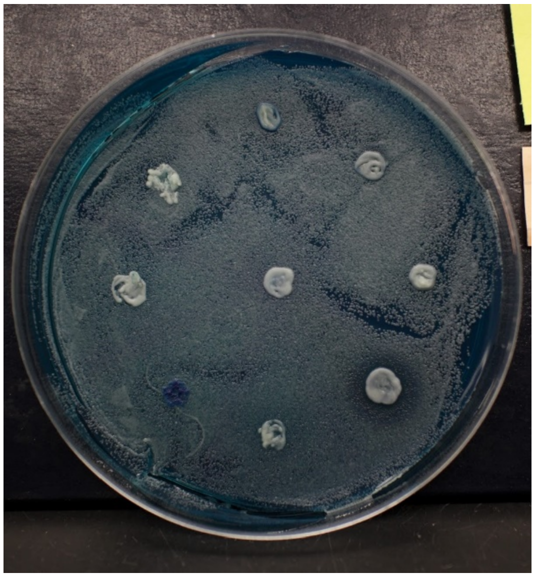

SSS 104

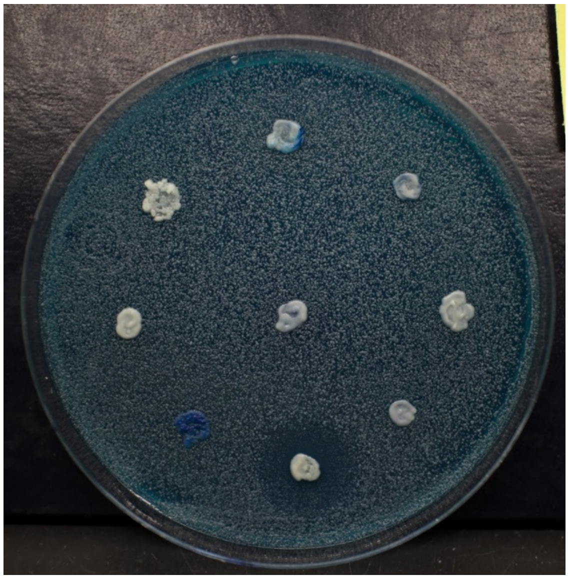

NCYC 2729

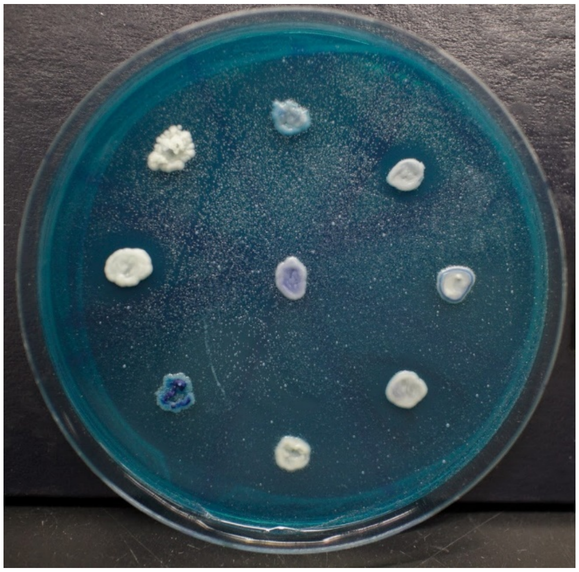

BY4741

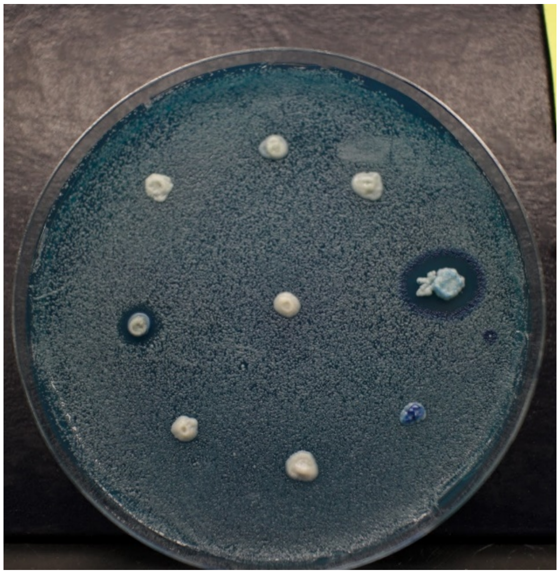

Key

<table>
<tr><td colspan="6">Y-63748</td></tr>
<tr><td colspan="3">Y-63705</td><td colspan="3">Y-63749</td></tr>
<tr><td colspan="2">Y-63704</td><td colspan="2">Y-63706</td><td colspan="2">Y-27340</td></tr>
<tr><td colspan="3">Y-27471</td><td colspan="3">Y-27341</td></tr>
<tr><td colspan="6">Y-27342</td></tr>
</table>

BY4741

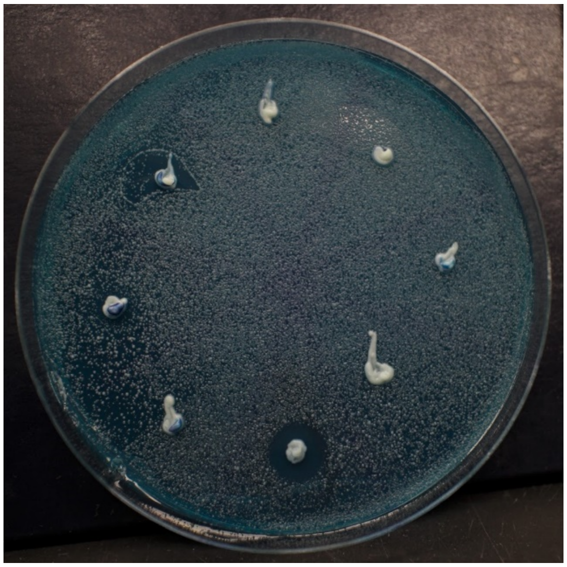

Key

<table>
<tr><td colspan="6">Y-788</td></tr>
<tr><td colspan="3">Y-1912</td><td colspan="3">Y-863</td></tr>
<tr><td colspan="2">Y-1548</td><td colspan="2">Y-2038</td><td colspan="2">Y-911</td></tr>
<tr><td colspan="3">Y-1356</td><td colspan="3">Y-1088</td></tr>
<tr><td colspan="6">Y-1344</td></tr>
</table>

K12

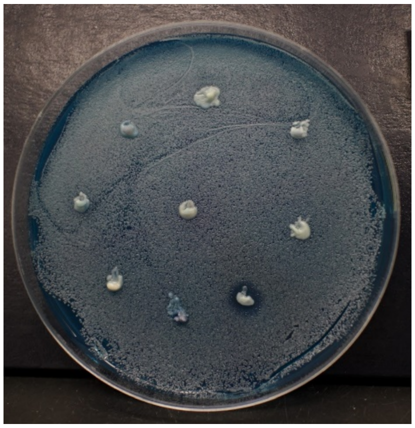

BY4741

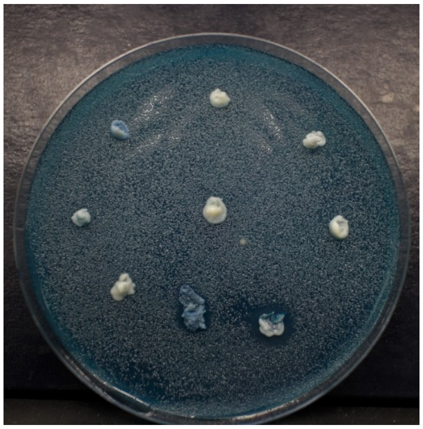

SSS 104

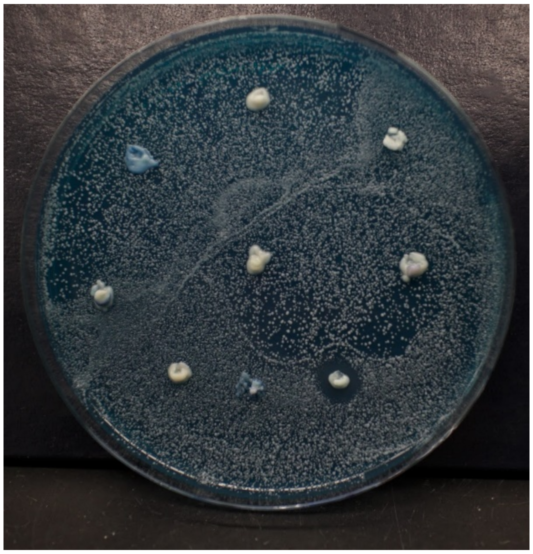

Key

YB-2047					
**Y-12602**			YB-4137		
Y-11842		Y-17218		**YB-4565**	
Y-6179			Y-5688		
Y-6177					

BY4741

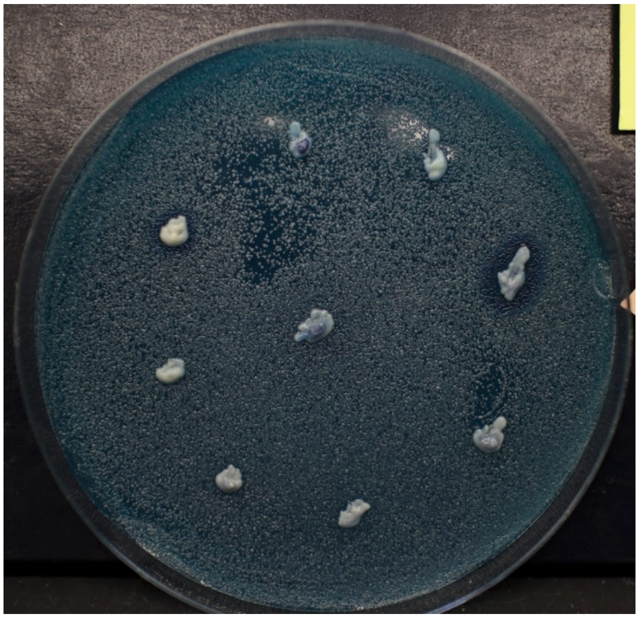

Key

<table>
<tr><td colspan="6" align="center">Y-17353</td></tr>
<tr><td colspan="3" align="center">Y-63714</td><td colspan="3" align="center">Y-63708</td></tr>
<tr><td colspan="2" align="center">Y-63713</td><td colspan="2" align="center">Y-63715</td><td colspan="2" align="center">Y-63709</td></tr>
<tr><td colspan="3" align="center">Y-63712</td><td colspan="3" align="center">Y-63710</td></tr>
<tr><td colspan="6" align="center">Y-63711</td></tr>
</table>

BY4741

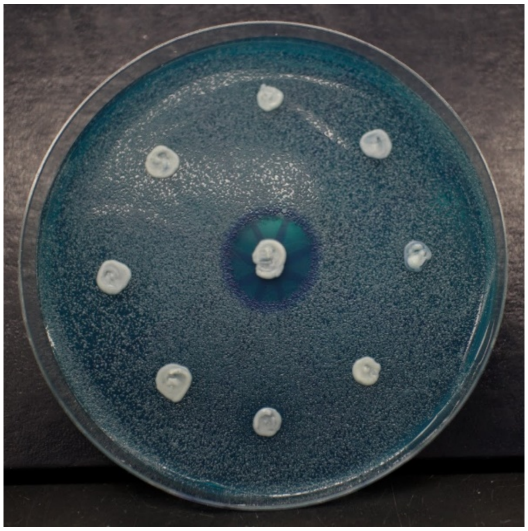

SSS 104

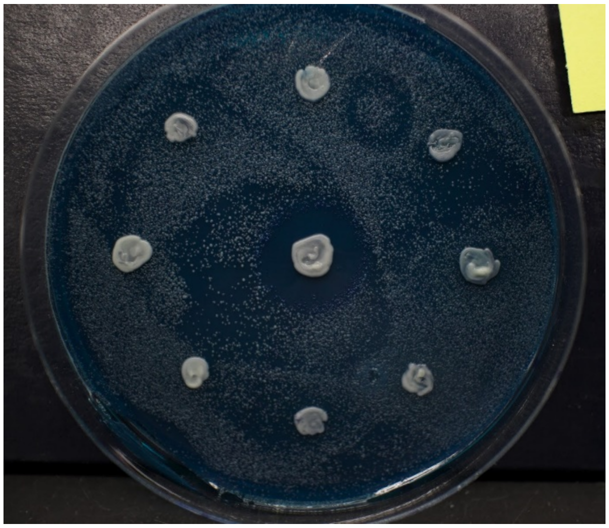

NCYC 2729

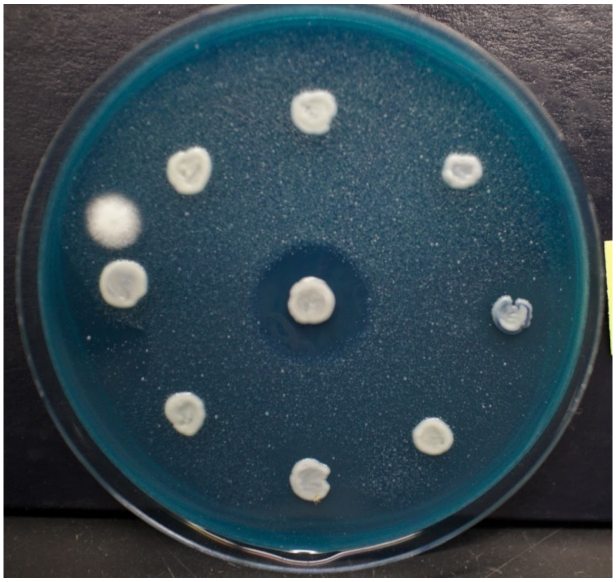

Key

<table>
<tr><td colspan="6">Y-63716</td></tr>
<tr><td colspan="3">1200</td><td colspan="3">Y-63717</td></tr>
<tr><td colspan="2">1130</td><td colspan="2">1133</td><td colspan="2">851</td></tr>
<tr><td colspan="3">1119</td><td colspan="3">859</td></tr>
<tr><td colspan="6">838</td></tr>
</table>

K12

BY4741

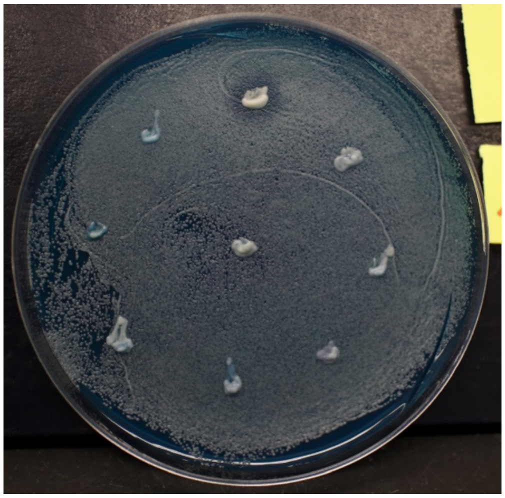

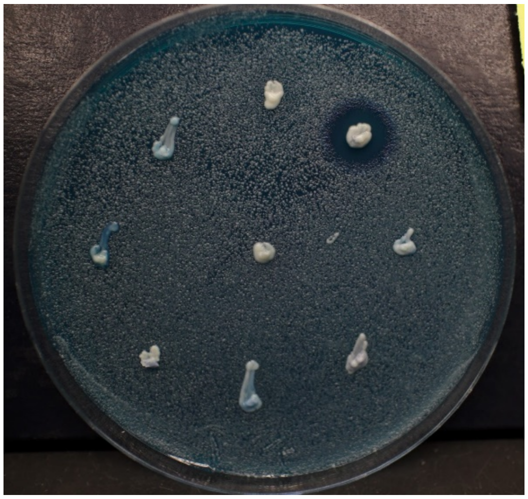

SSS 104

NCYC 2729

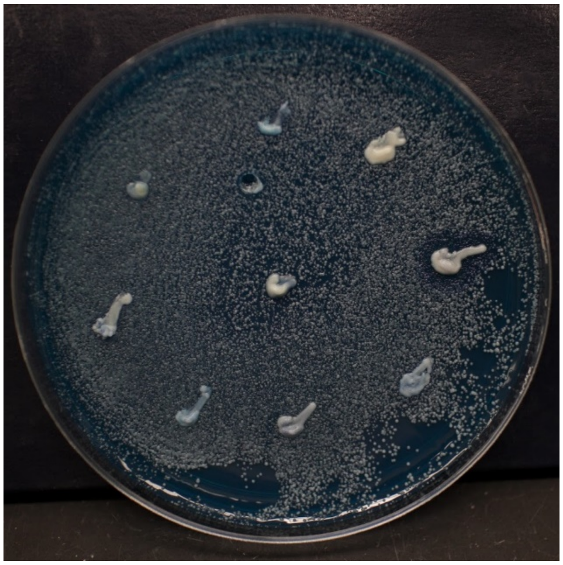

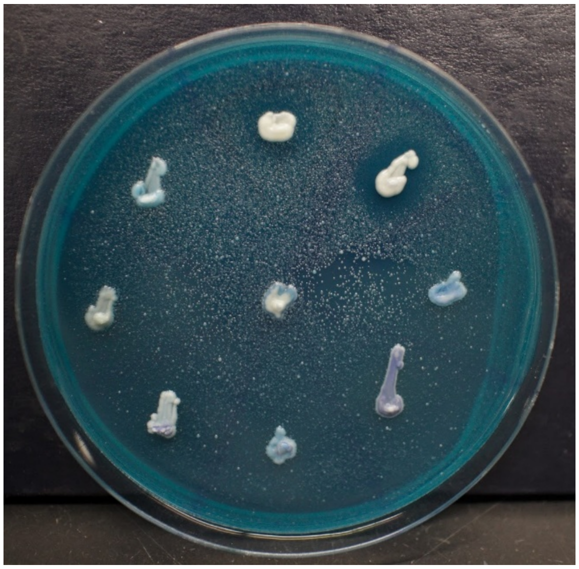

Key

Y-580					
Y-12624			**Y-670**		
Y-11845		Y-12646		Y-846	
Y-1374			Y-969		
Y-972					

NCYC 2729

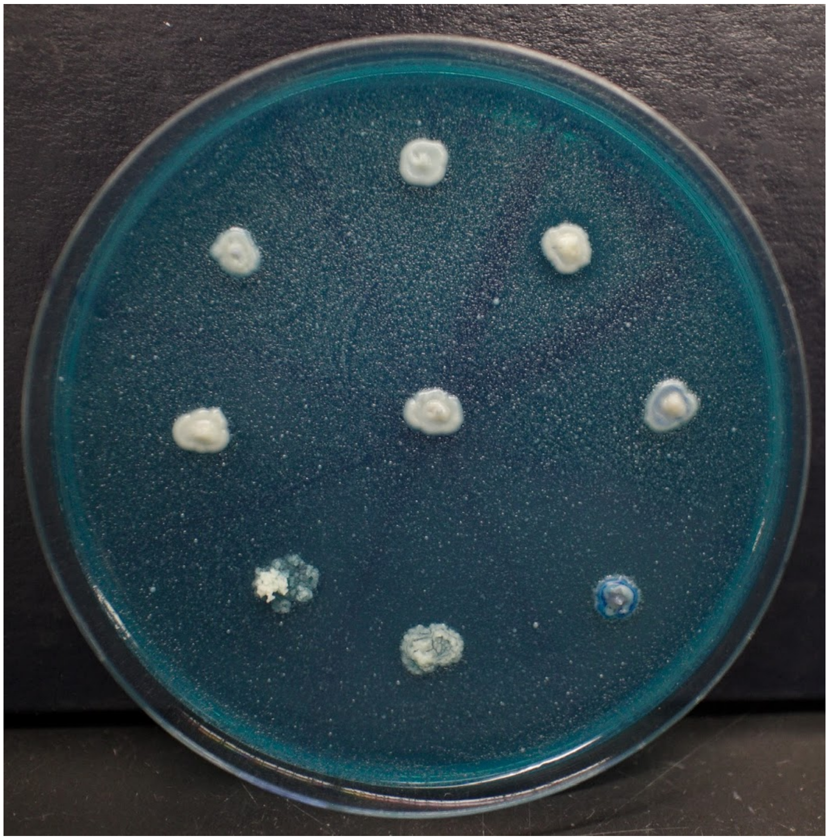

Key

Y-12648				
YB-254		Y-17034		
Y-63718	**YB-432**	Y-27339		
Y-63707		Y-27470		
Y-48770				

NCYC 2729

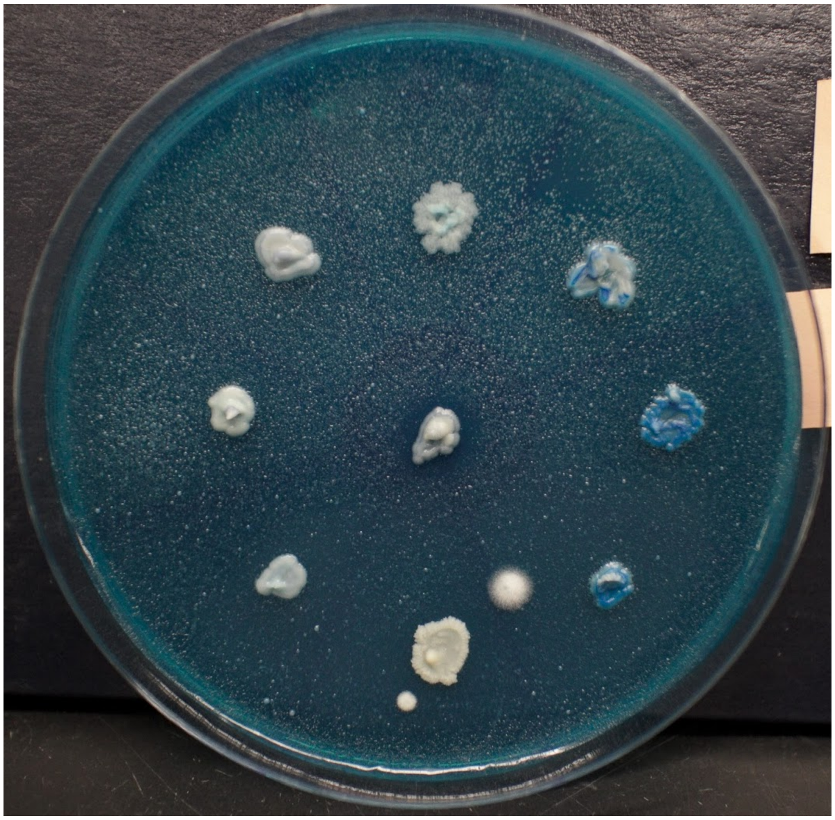

Key

<table>
<tr><td colspan="6">Y-567</td></tr>
<tr><td colspan="3">Y-975</td><td colspan="3">Y-851</td></tr>
<tr><td colspan="2">Y-954</td><td colspan="2">Y-976</td><td colspan="2">Y-852</td></tr>
<tr><td colspan="3">YB-908</td><td colspan="3">Y-897</td></tr>
<tr><td colspan="6">Y-898</td></tr>
</table>

K12

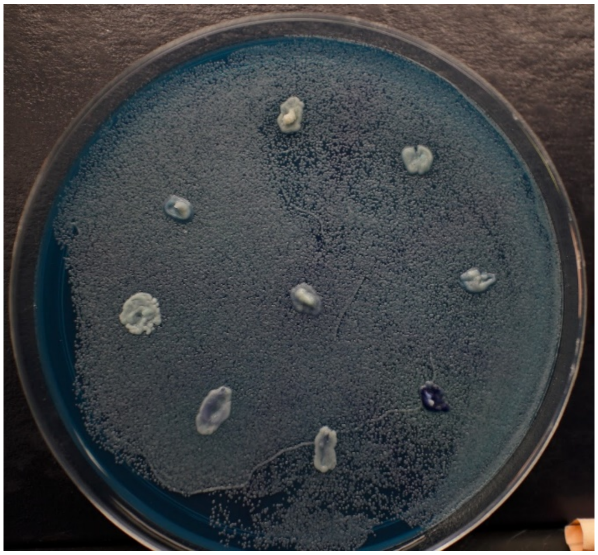

FY4

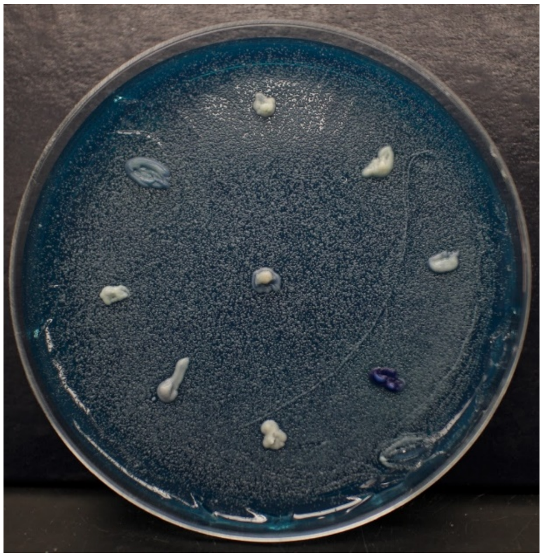

CBS 7001

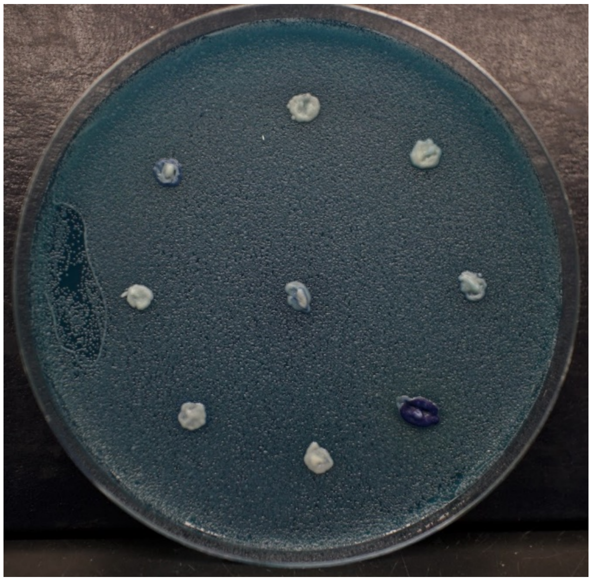

NCYC 2729

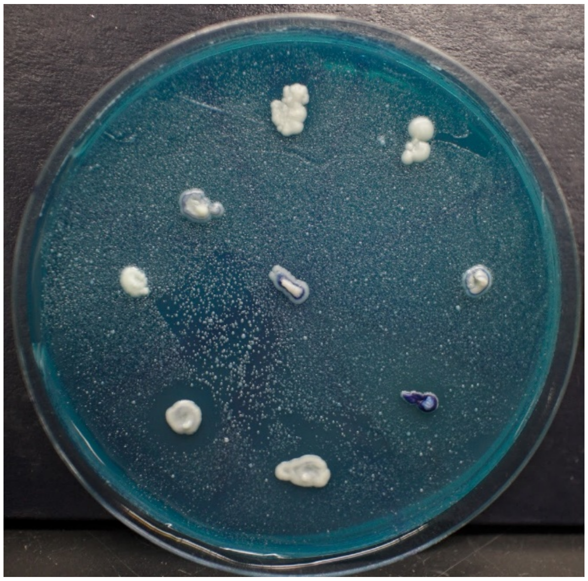

Key

<table>
<tr><td colspan="6">**Y-977**</td></tr>
<tr><td colspan="3">Y-1429</td><td colspan="3">Y-1018</td></tr>
<tr><td colspan="2">Y-1428</td><td colspan="2">Y-1430</td><td colspan="2">Y-1089</td></tr>
<tr><td colspan="3">Y-1370</td><td colspan="3">Y-1285</td></tr>
<tr><td colspan="6">Y-1301</td></tr>
</table>

NCYC 2729

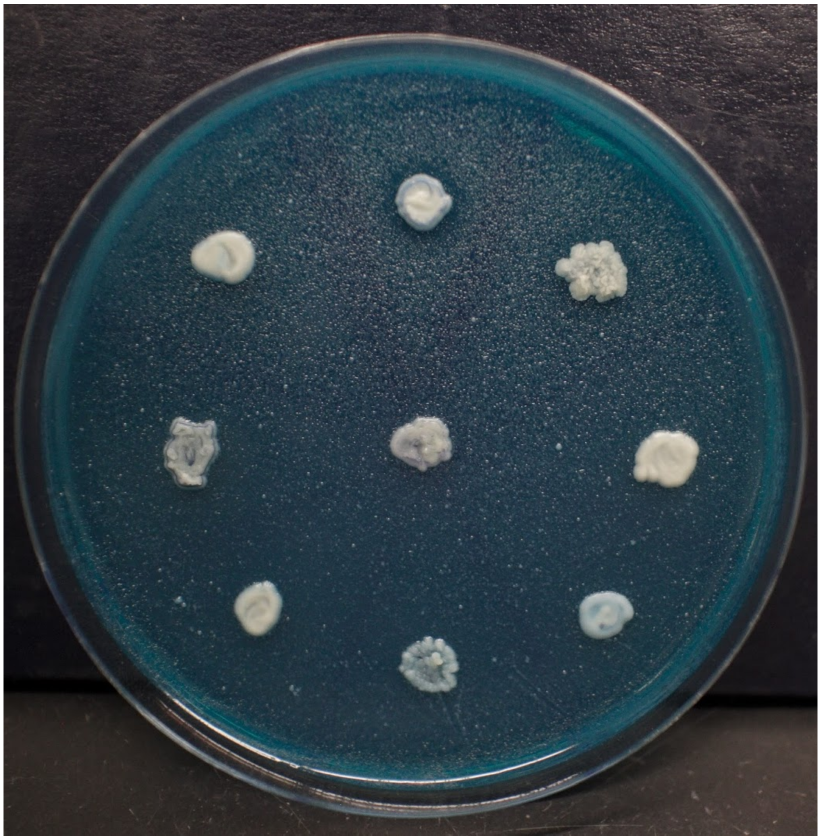

Supplement: S1 File — (PDF) [file pgen.1009341.s016.pdf]
