# Supplementary material for: The Species-Specific Acquisition and Diversification of a K1-like Family of Killer Toxins in Budding Yeasts of the Saccharomycotina
Source: PLoS Genet. 2021 Feb 4;17(2):e1009341. doi: 10.1371/journal.pgen.1009341 (PMC7888664; doi:10.1371/journal.pgen.1009341)

**File S2. Image data of killer phenotypes exhibited by strains of *Saccharomyces* yeasts as summarized in figure 1.** The organization of the 21 strains of *Saccharomyces* yeasts arrayed on killer assay agar plates. Each killer yeast was assayed for toxin activity against 47 different lawns of susceptible yeast strains. n/a: not applicable.

**PLATE KEY (Pages 2-13)**

|  | 1 | 2 | 3 | 4 | 5 | 6 | 7 | 8 |
| --- | --- | --- | --- | --- | --- | --- | --- | --- |
| A | n/a | - | BJH001 | - | NCYC 190 | - | n/a | - |
| B | - | Y-2429 | - | 1116 | - | NCYC 1001 | - | MS300C |
| C | CYC 1058 | - | CYC 1113 | - | n/a | - | n/a | - |
| D | - | Y8.5 | - | Y-63717 | - | Y-63716 | - | Y-63711 |
| E | Y-12602 | - | YB-4565 | - | Y-1088 | - | n/a | - |
| F | - | n/a | - | - | - | - | - | - |


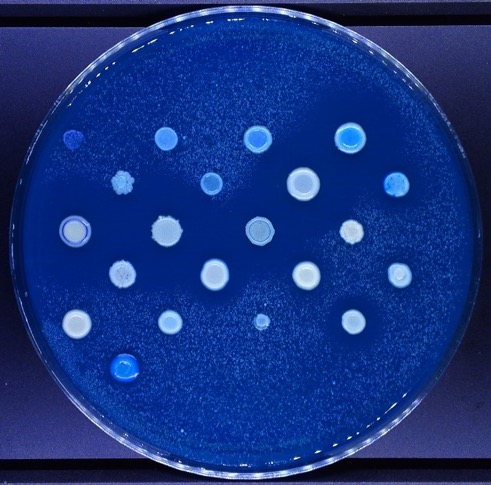

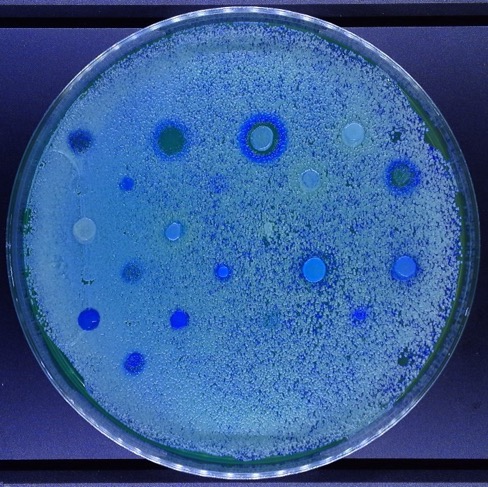
Lawn: BJH001 Lawn: NCYC 738


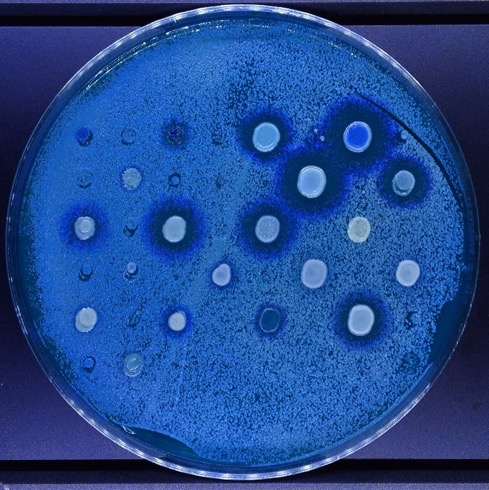

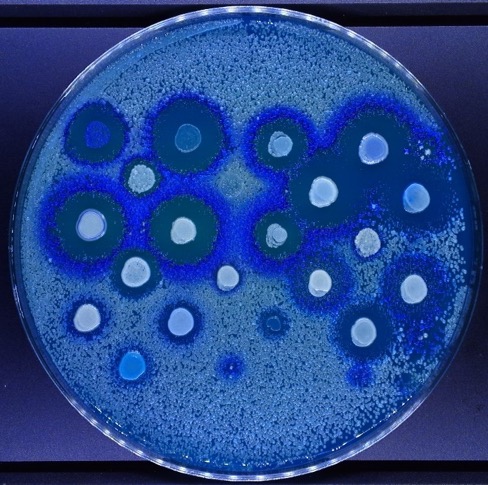
Lawn: DSM70459 Lawn: DBVPG 1373


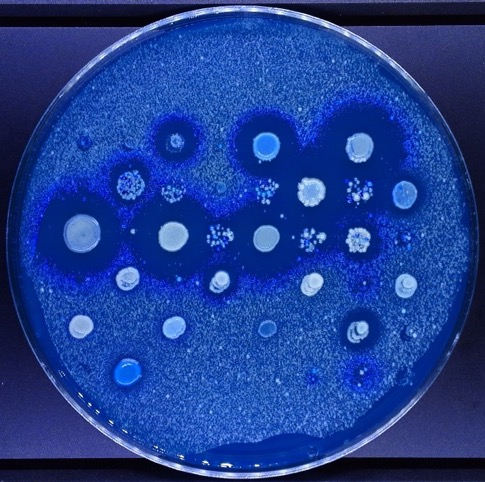

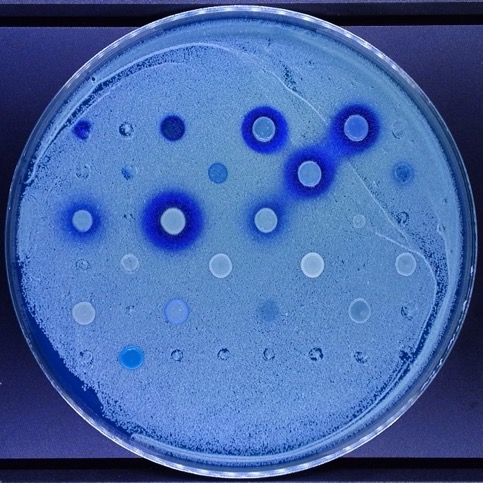
Lawn: Y-27788 Lawn: Y-27106


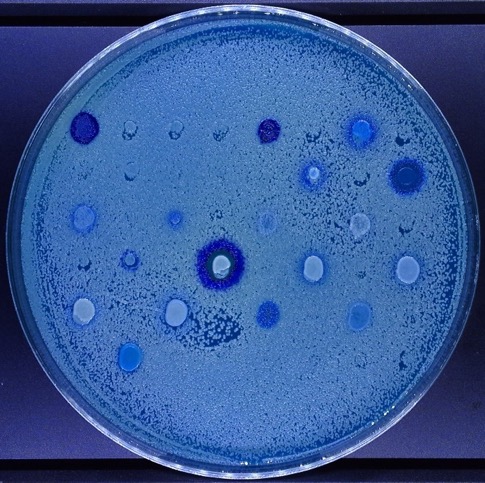
Lawn: Y-5509 Lawn: Y-1891


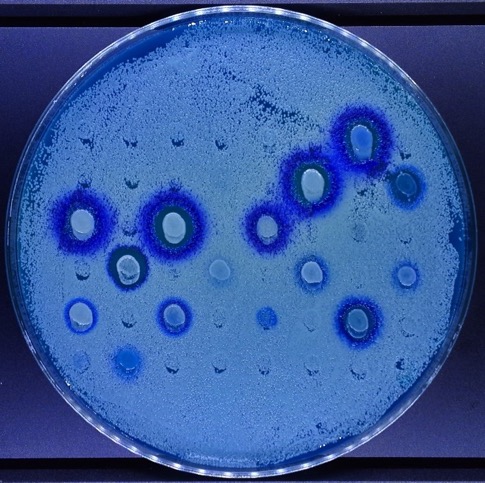


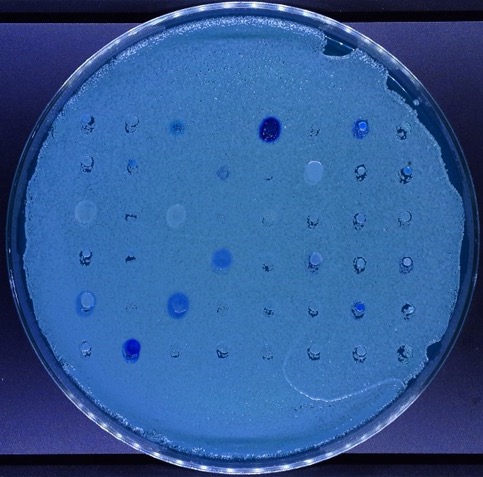

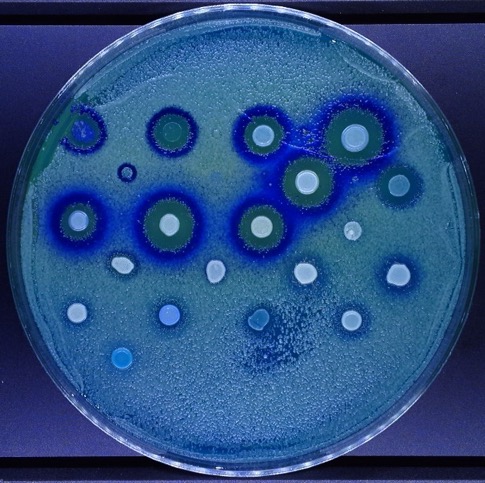
 Lawn: YB 432 Lawn: NCYC 1001


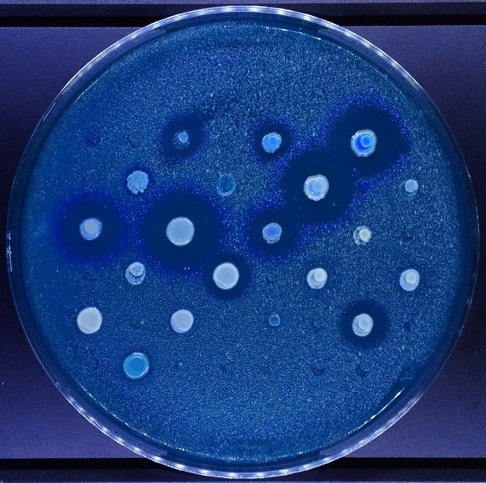
 Lawn: NCYC 1006 Lawn: Ms300C


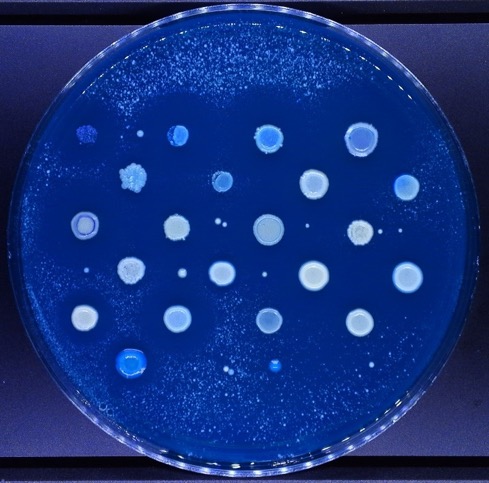


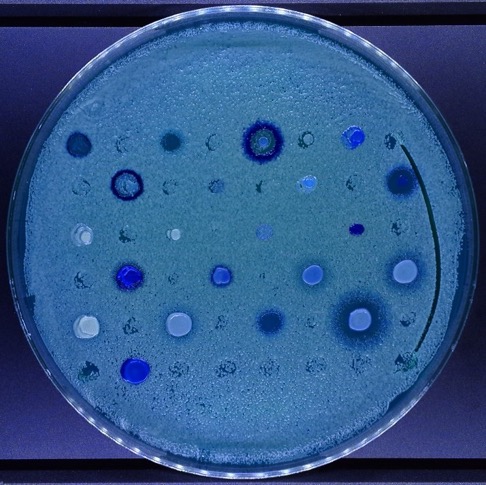
 Lawn: CYC 1058 Lawn: CYC 1113


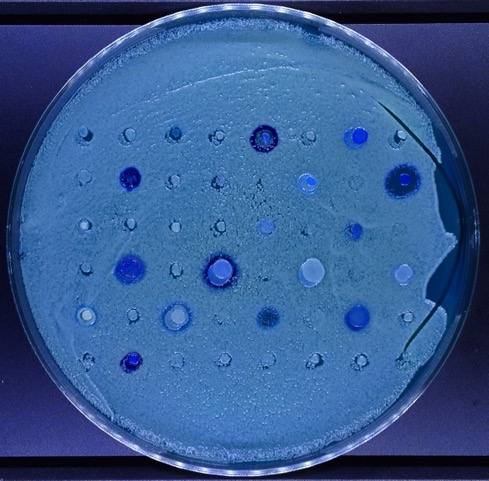


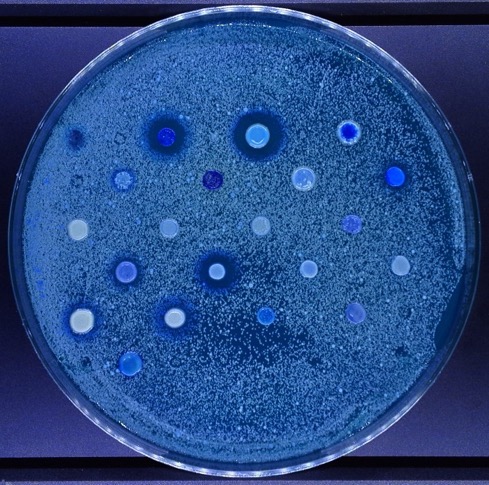

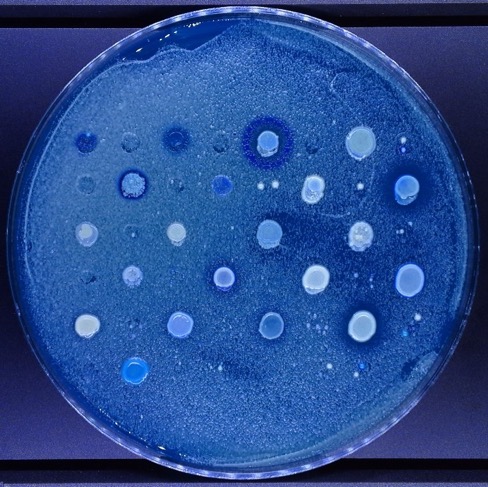
 Lawn: CYC 1170 Lawn: CYC 1172


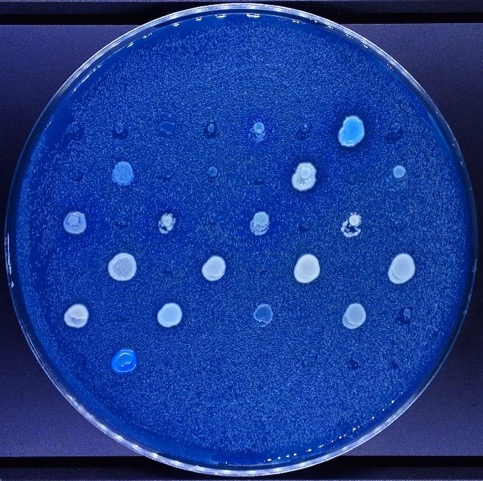

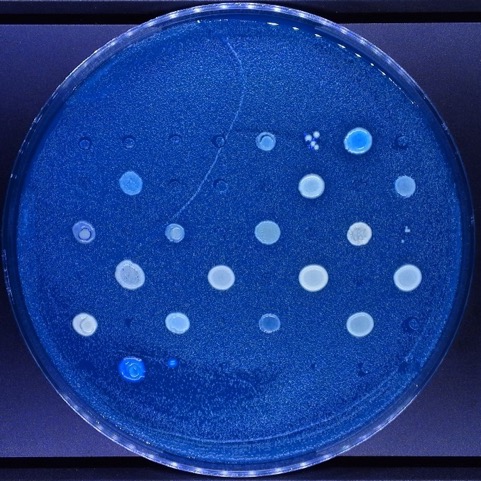
 Lawn: Y-1088 Lawn: Y-27342


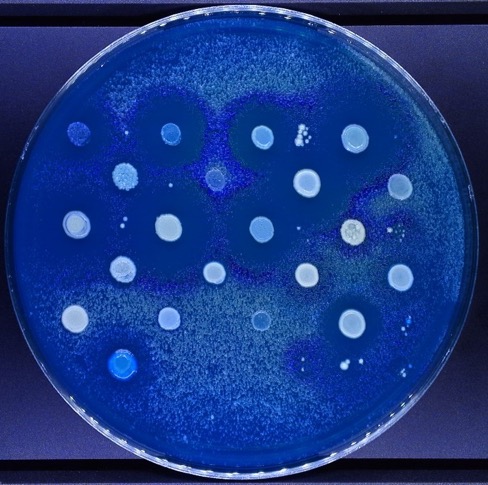

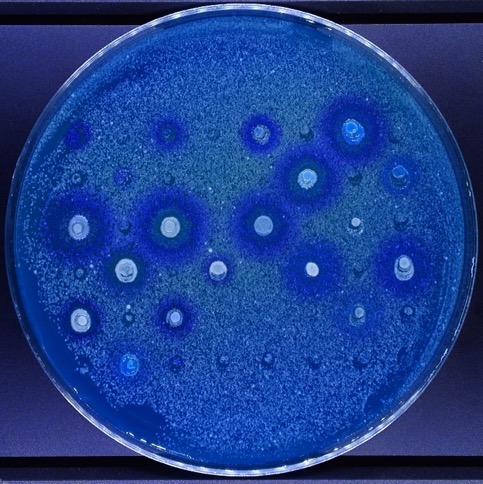
 Lawn: Y-2046 Lawn: Y-1344


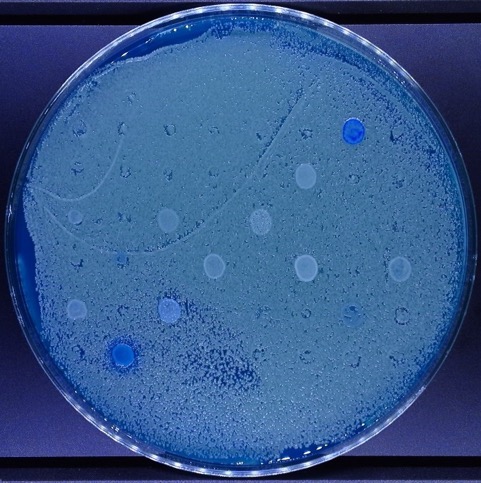
 Lawn: NCYC 777 Lawn: NCYC 2898


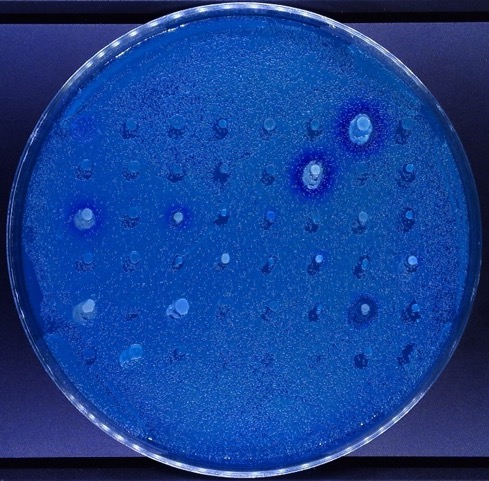


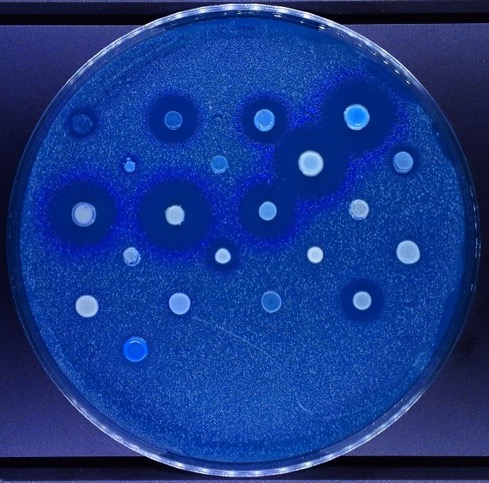
 Lawn: 2729 Lawn: FY4


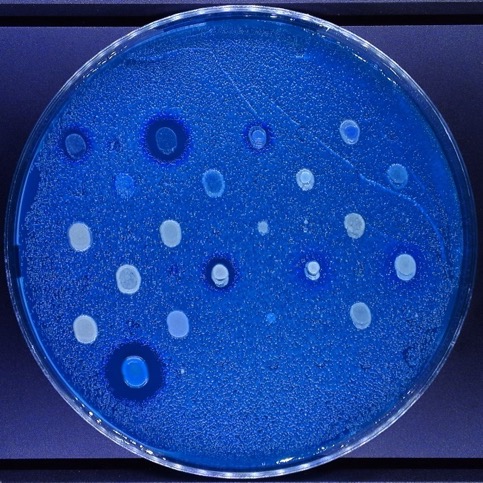


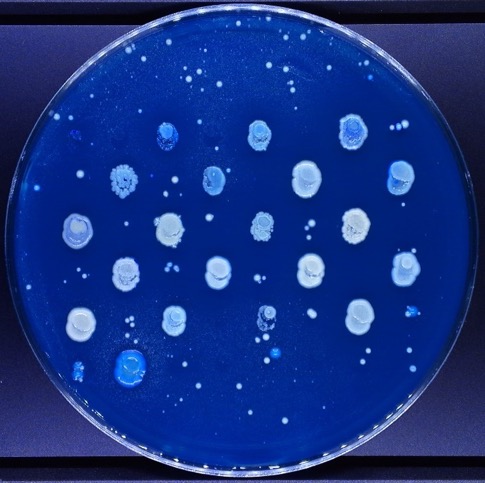
 Lawn: K12 Lawn: BY4741


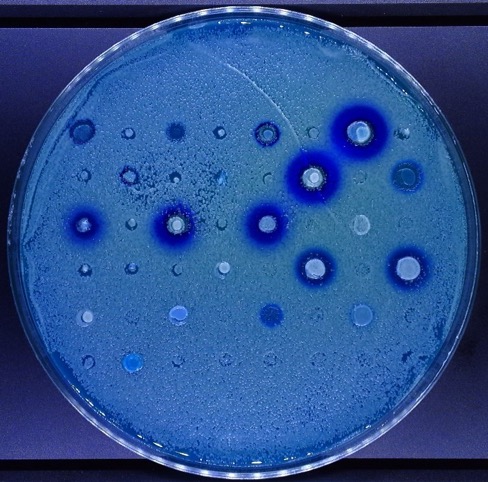


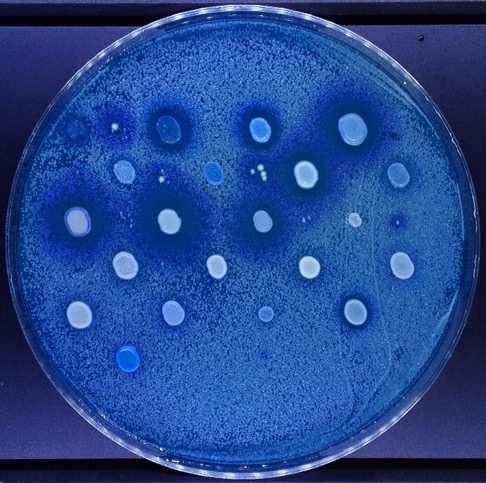
 Lawn: DBVPG 6765 Lawn: CYC 1102


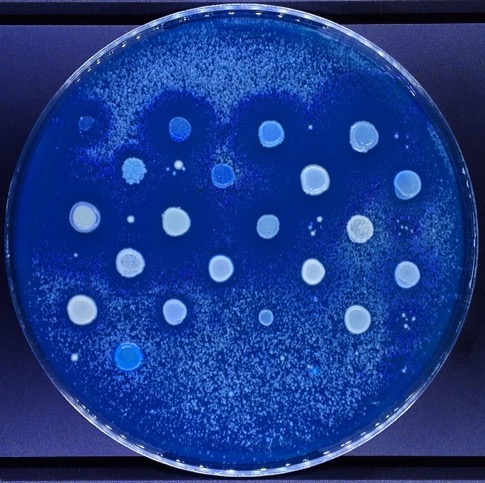


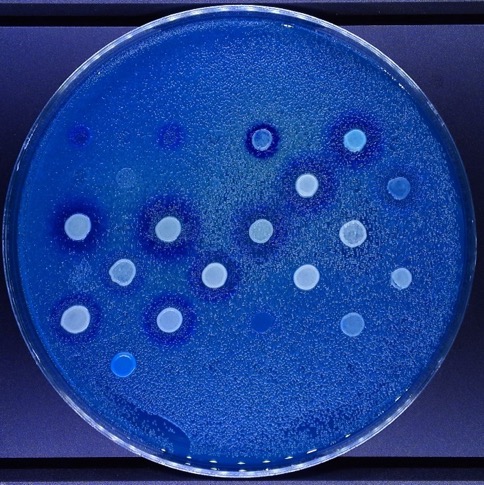
 Lawn: YB-4237 Lawn: CBS 432


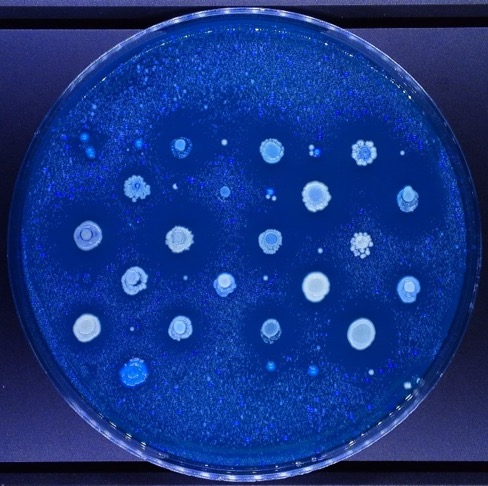


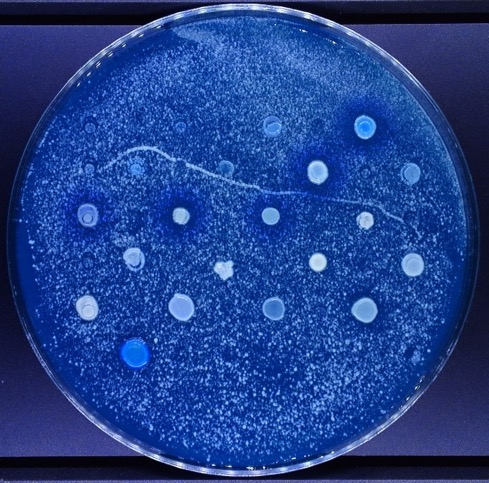
 Lawn: A12C Lawn: NBRC 1815


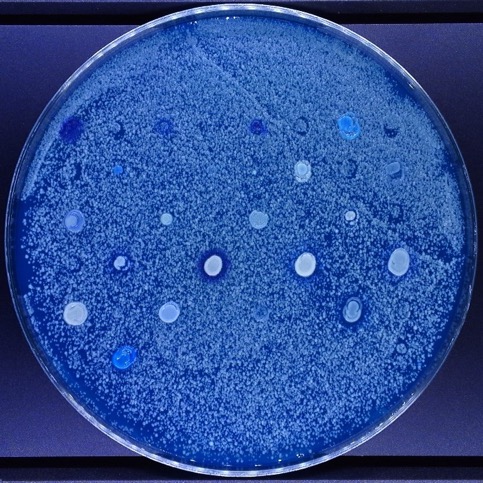


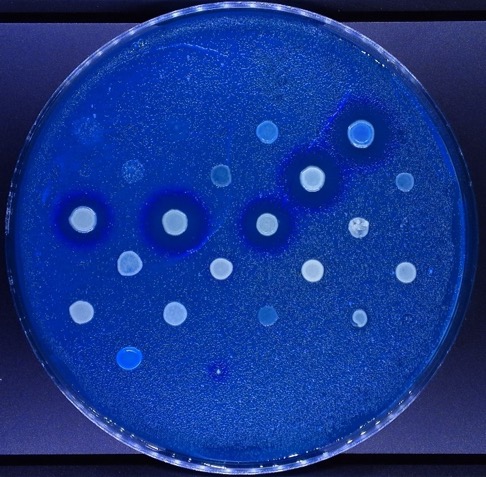
 Lawn: SSS 211 Lawn: NBRC 1802


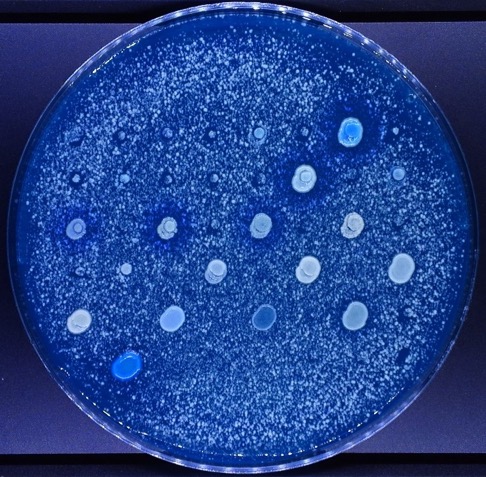


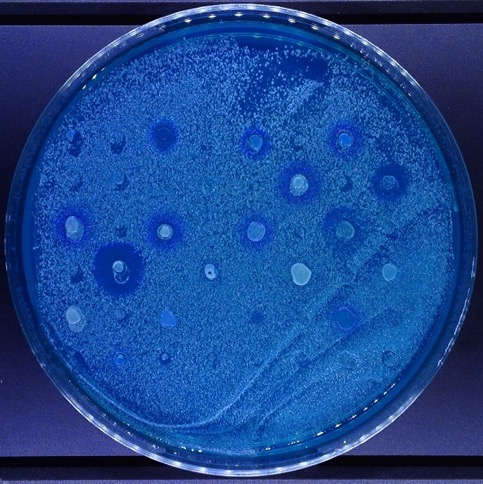
 Lawn: CBS 7001 Lawn: SSS 104


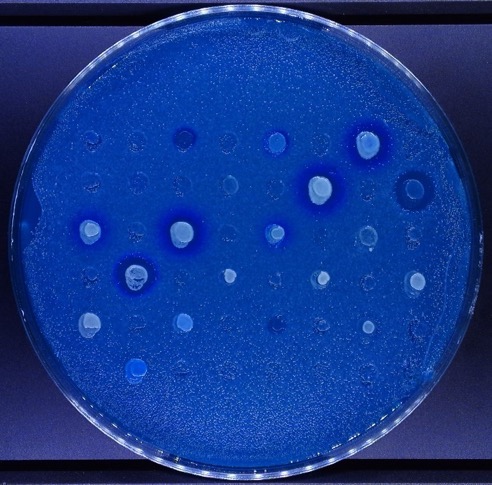


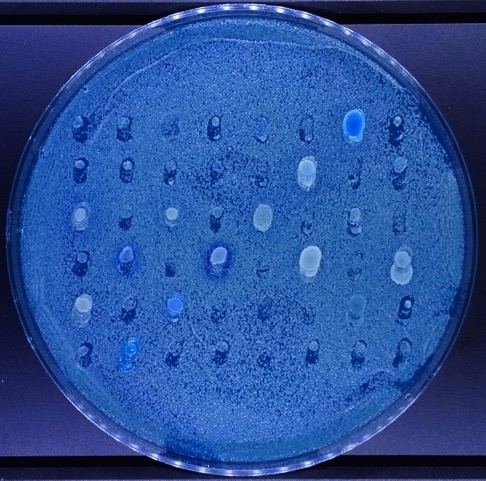
 Lawn: Y-63711 Lawn: Y-63716


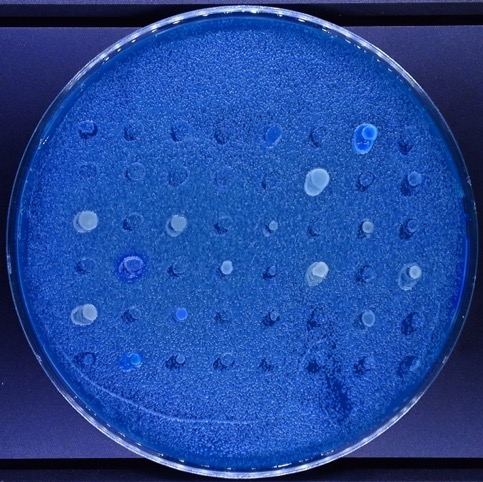


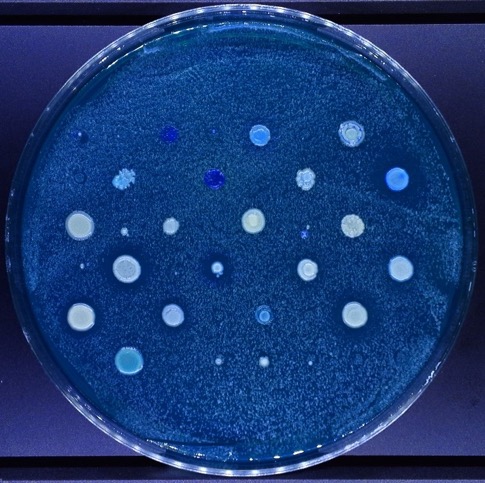
 Lawn: YB-4565 Lawn: 1116


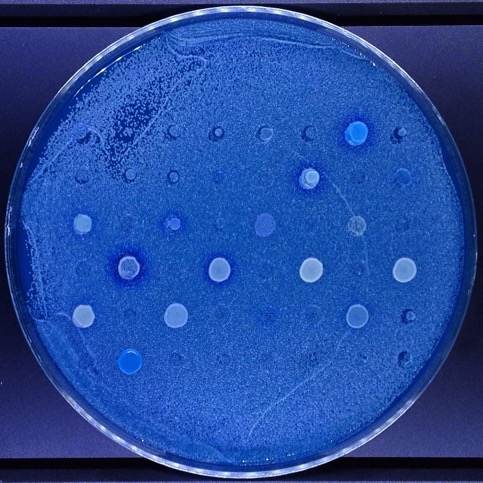


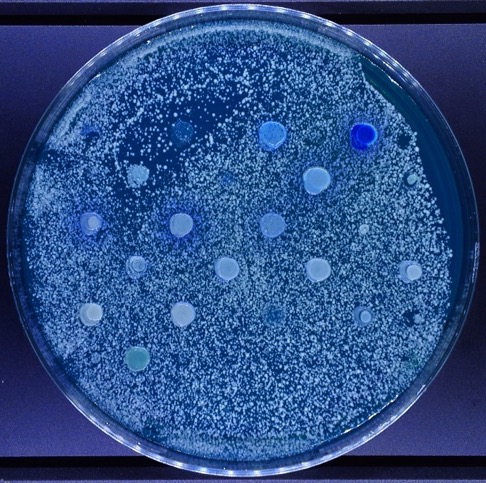
 Lawn: Y-2429 Lawn: DBVPG 6304


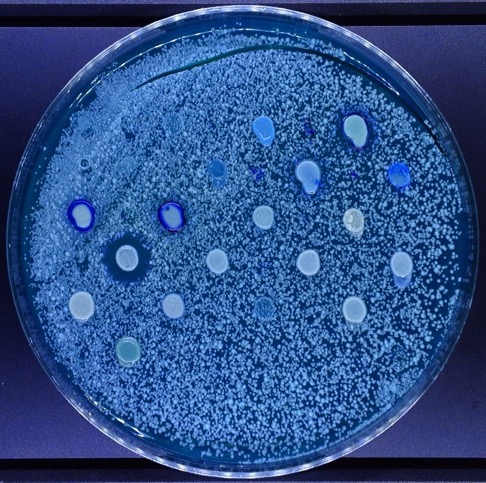


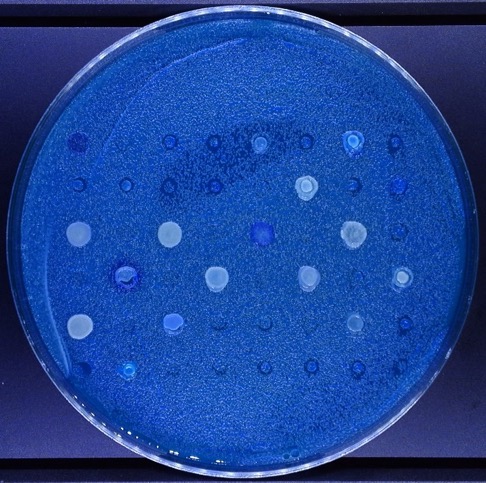

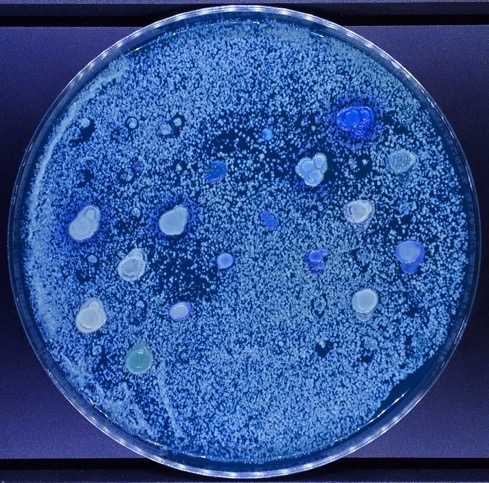
 Lawn: Y-63717 Lawn: Y8.5

Lawn: Y-12602


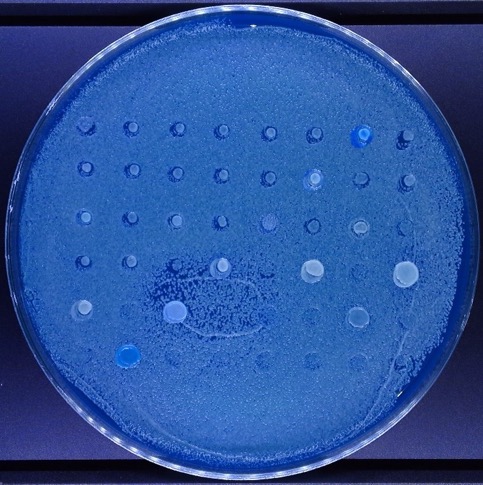


PLATE KEY (Pages 15-37)

|  | 1 | 2 | 3 | 4 | 5 | 6 | 7 | 8 | 9 | 10 | 11 | 12 |
| --- | --- | --- | --- | --- | --- | --- | --- | --- | --- | --- | --- | --- |
| A | - | - | - | - | - | - | - | - | - | - | - | - |
| B | - | n/a | n/a | n/a | n/a | n/a | n/a | n/a | n/a | n/a | Y-27788 | - |
| C | - | Y-27106 | n/a | Y-1891 | YB-432 | n/a | n/a | n/a | n/a | n/a | n/a | - |
| D | - | n/a | n/a | n/a | n/a | n/a | n/a | n/a | n/a | n/a | n/a | - |
| E | - | n/a | n/a | n/a | n/a | n/a | Y-2046 | Y-1344 | n/a | n/a | n/a | - |
| F | - | n/a | n/a | n/a | n/a | n/a | n/a | n/a | n/a | n/a | n/a | - |
| G | - | n/a | n/a | n/a | n/a | n/a | n/a | n/a | n/a | - | - | - |
| H | - | - | - | - | - | - | - | - | - | - | - | - |

Lawn: BJH001


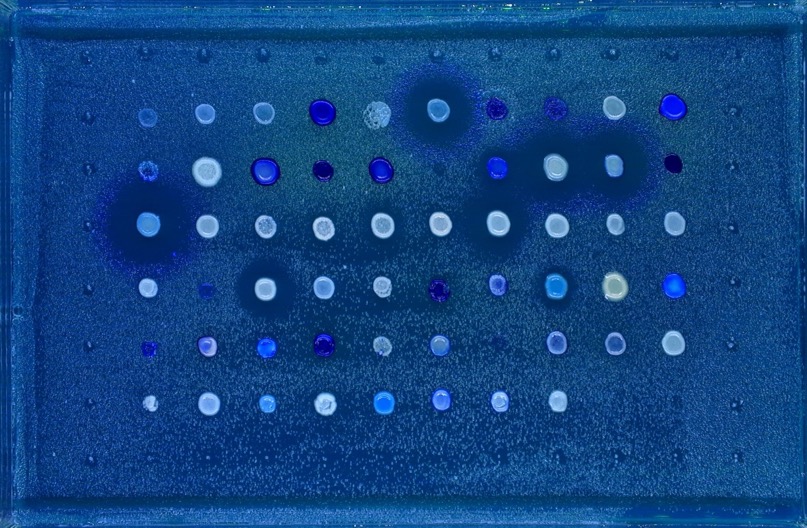


Lawn: NCYC 738


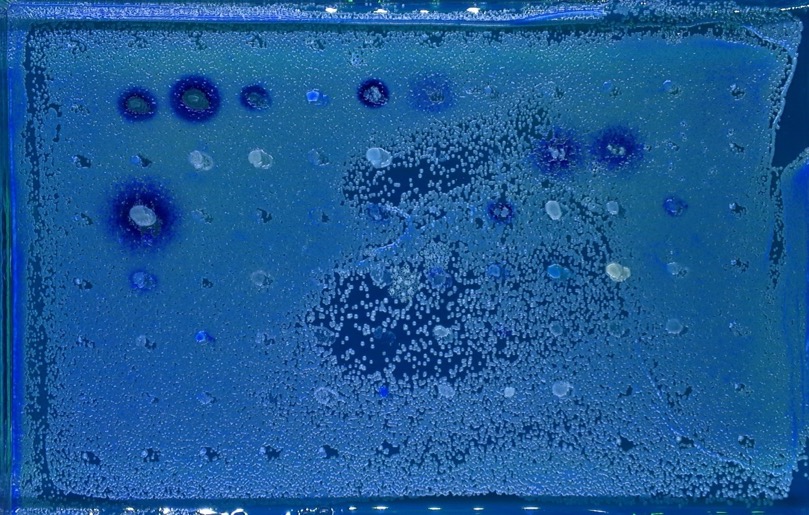


Lawn: DSM 70459


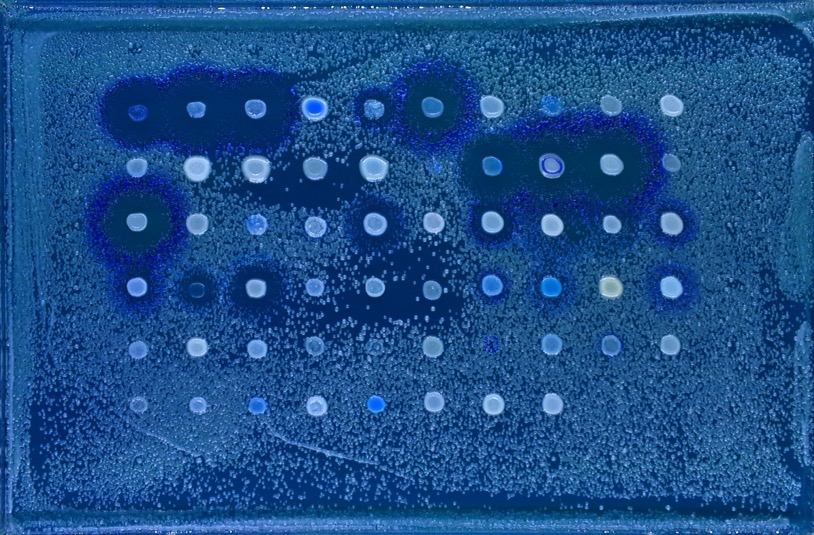


Lawn: DBVPG 1373


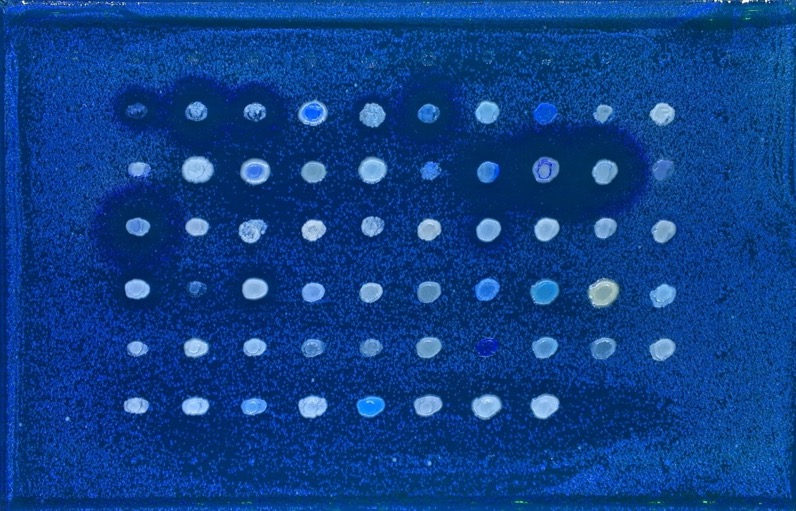


Lawn: Y-27788


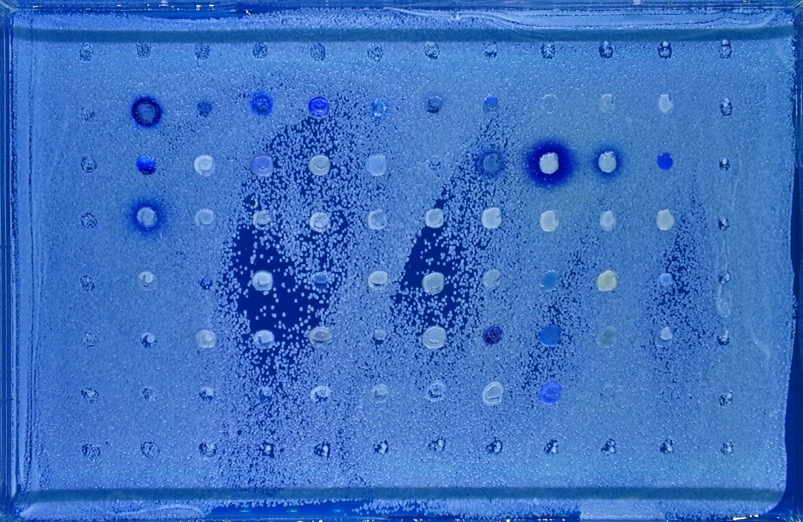


Lawn: Y-27106


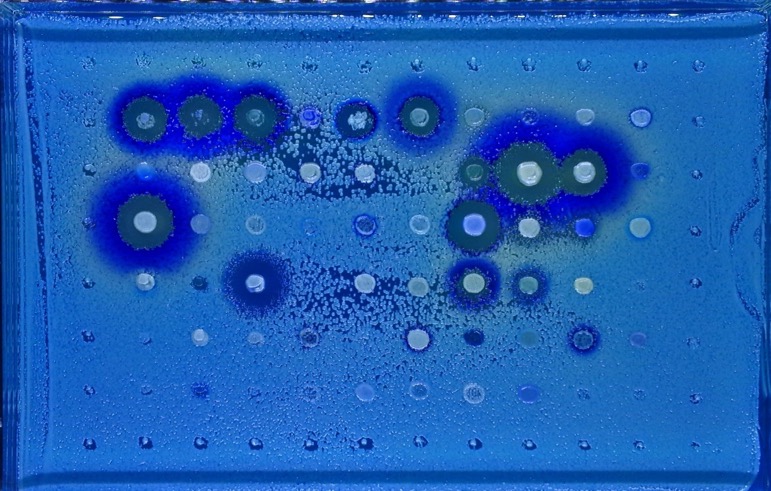


Lawn: Y-5509


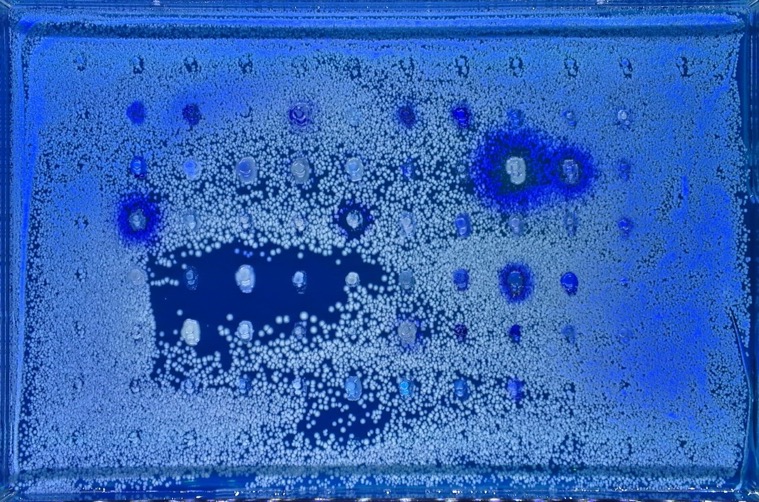


Lawn: Y-1891


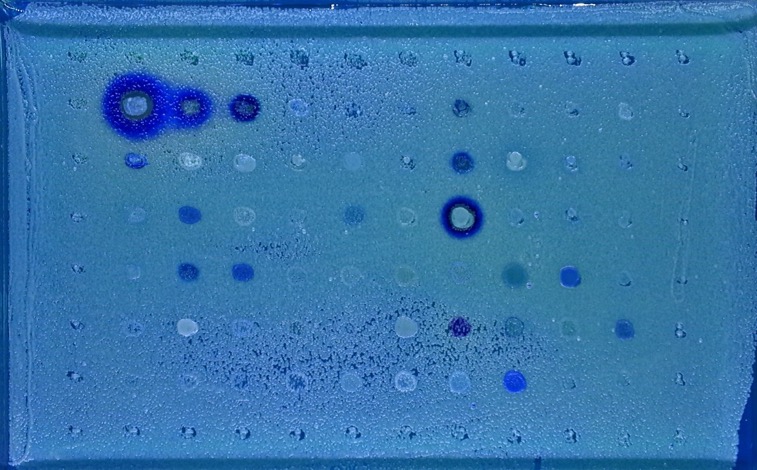


Lawn: YB-432


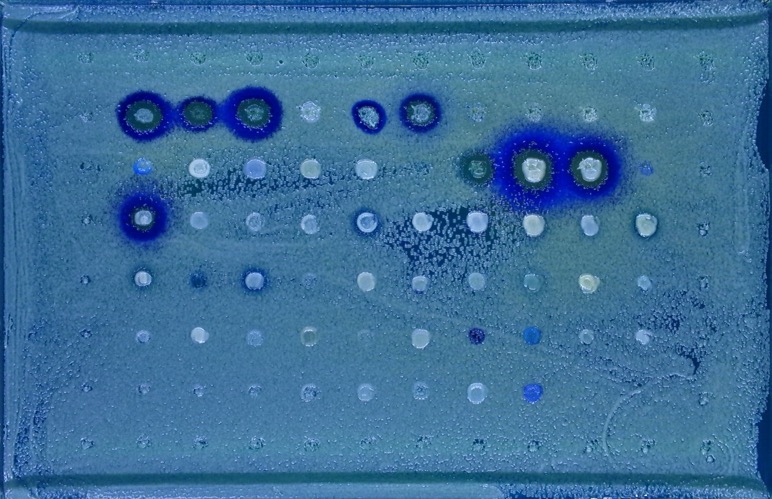


Lawn: NCYC 1001


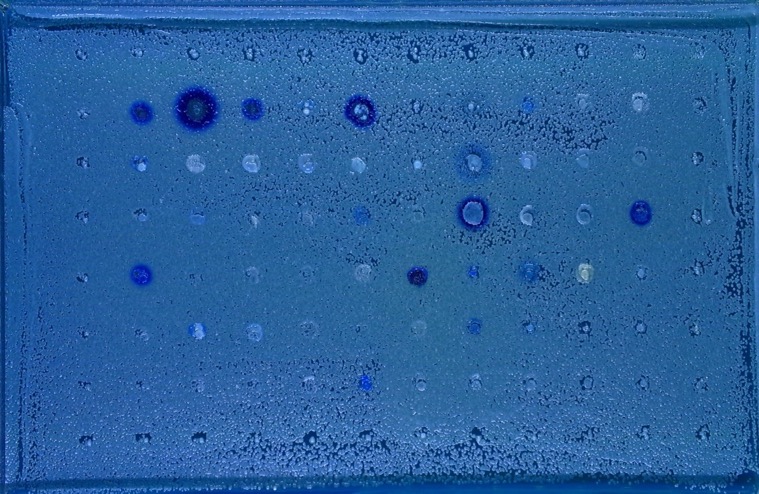


Lawn: NCYC 1006


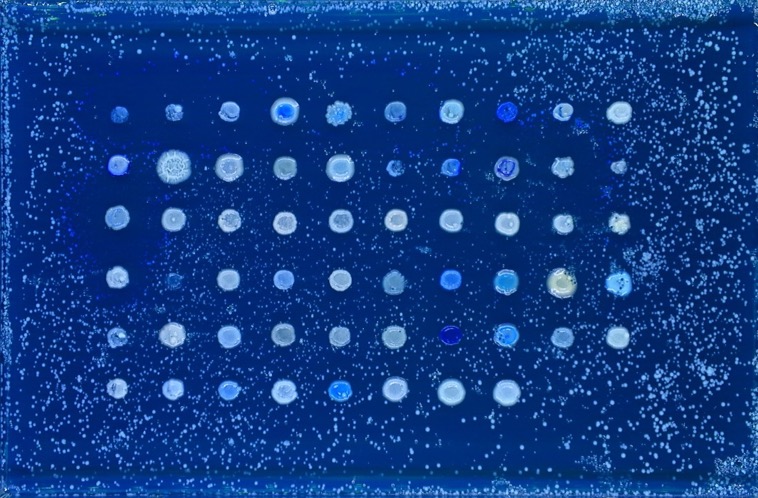


Lawn: MS 300c


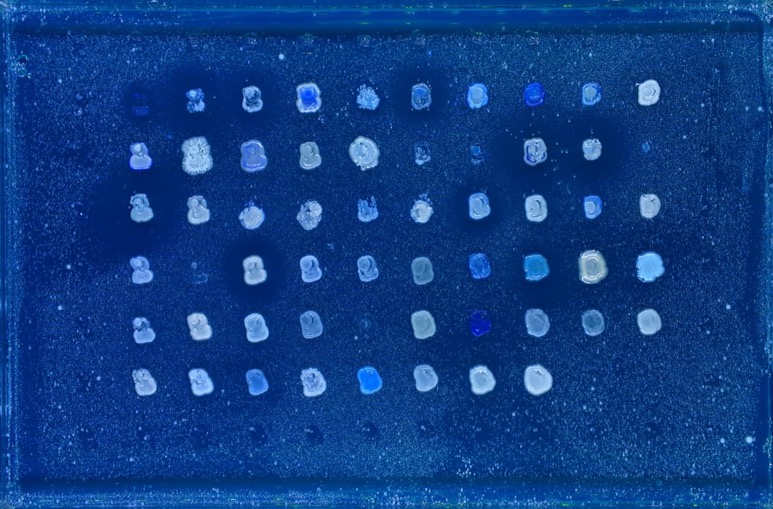


Lawn: CYC 1058


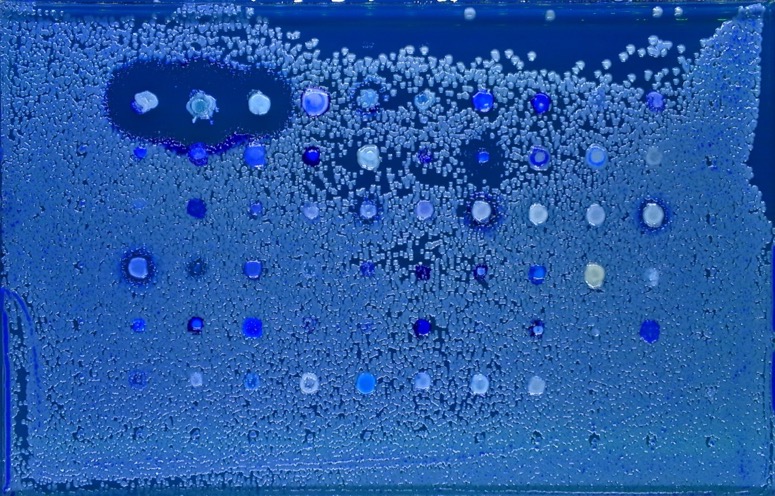


Lawn: CYC 1113


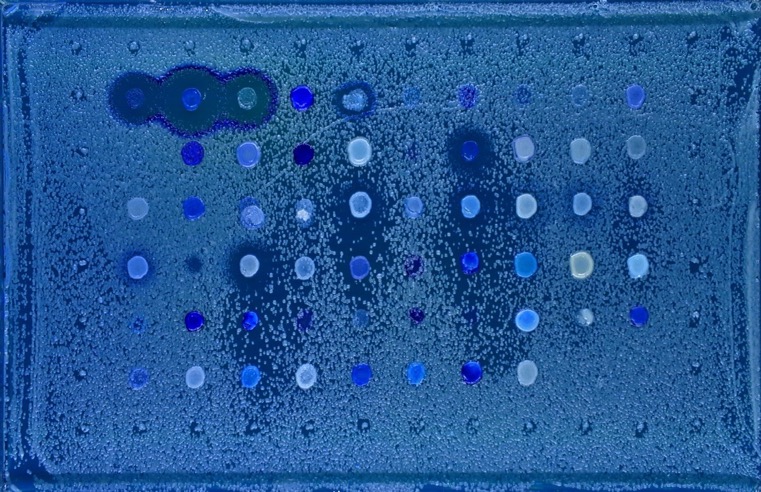


Lawn: CYC 1170


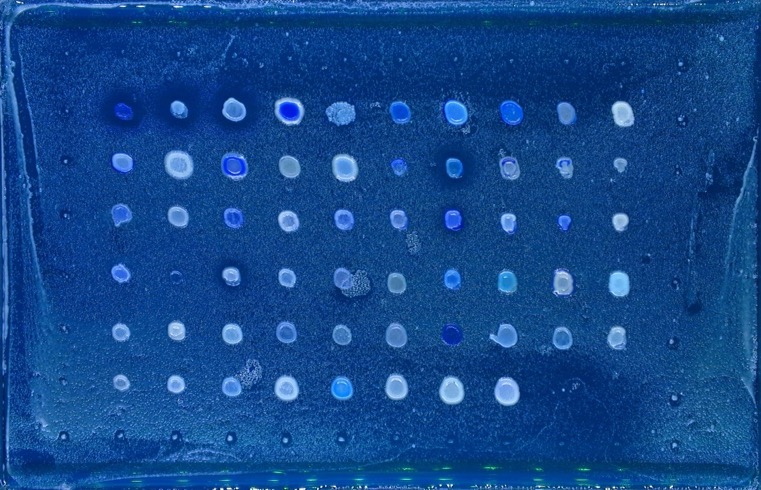


Lawn: CYC 1172


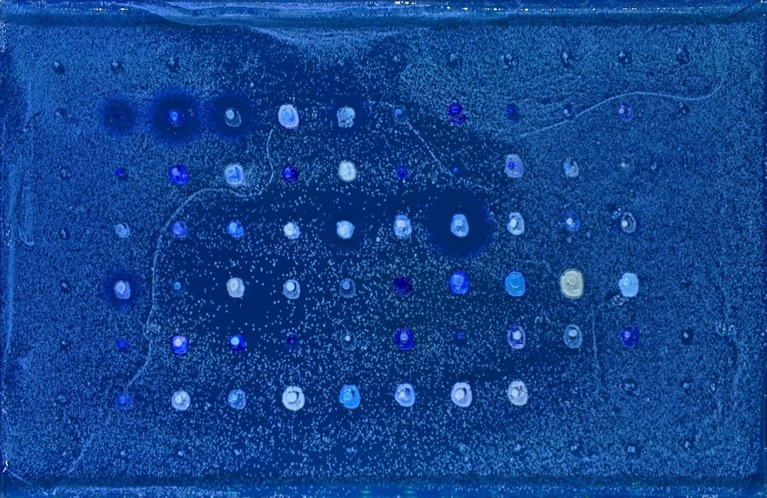


Lawn: Y-1088


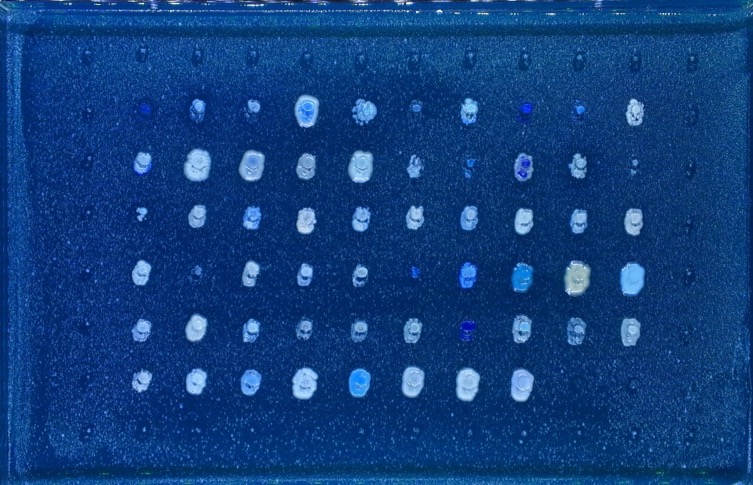


Lawn: Y-27342


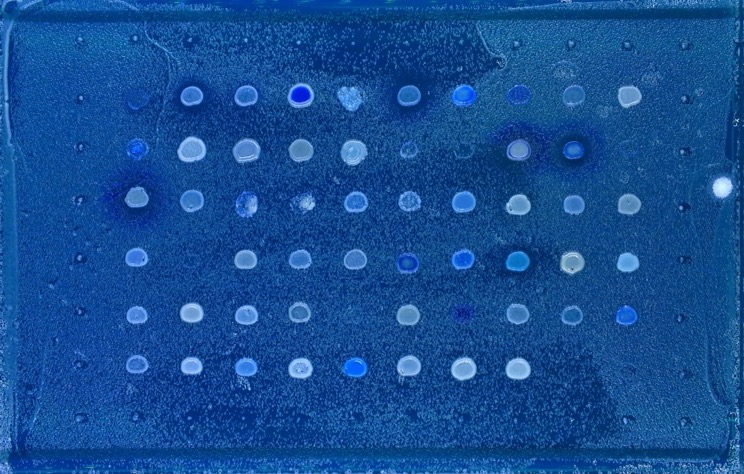


Lawn: Y-2046


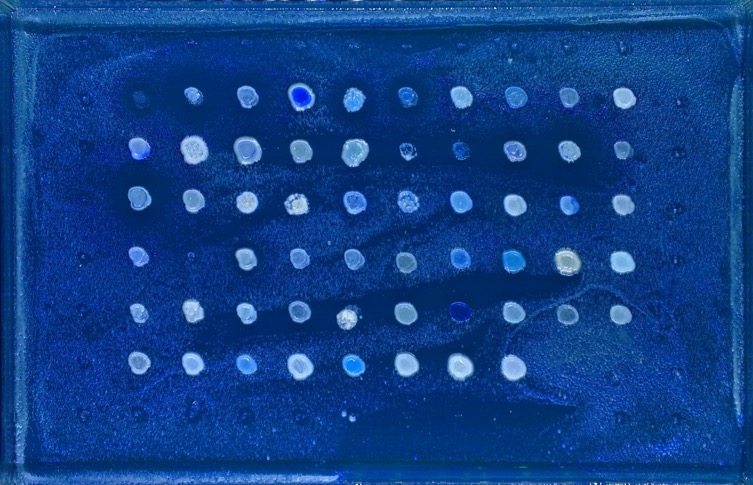


Lawn: Y-1344


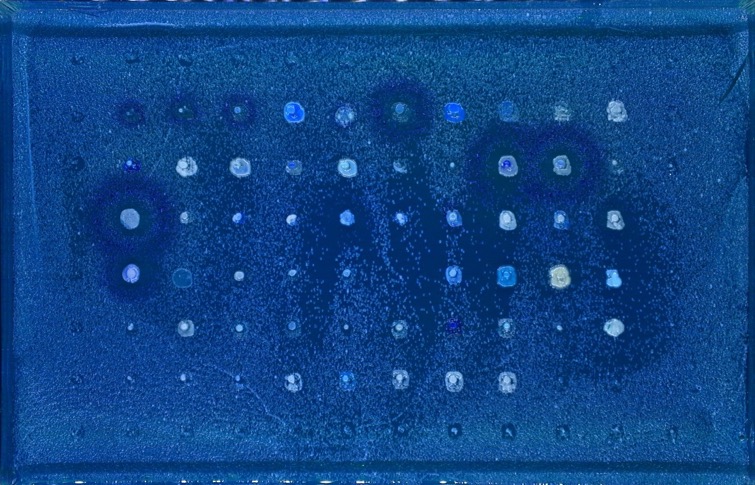


Lawn: NCYC 777


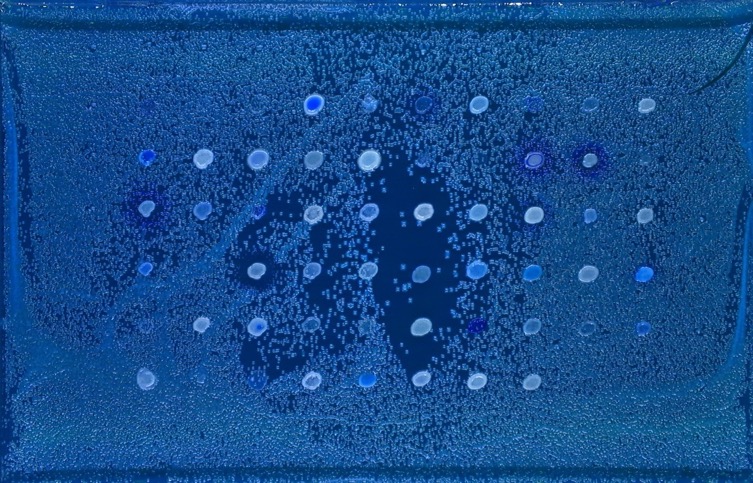


Lawn: NCYC 2898


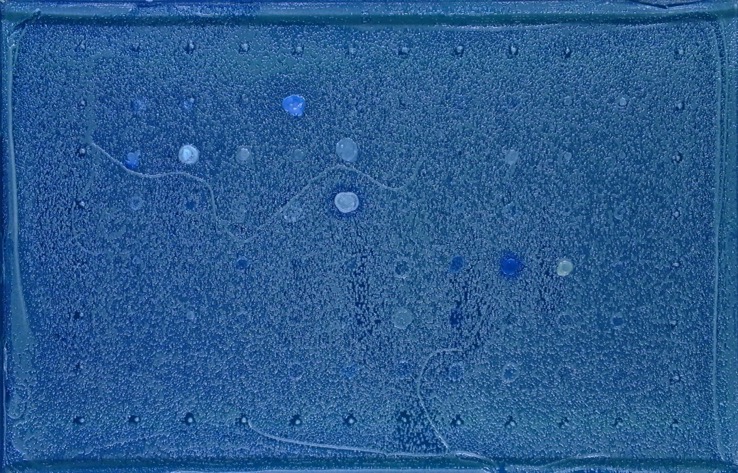


Lawn: 2729


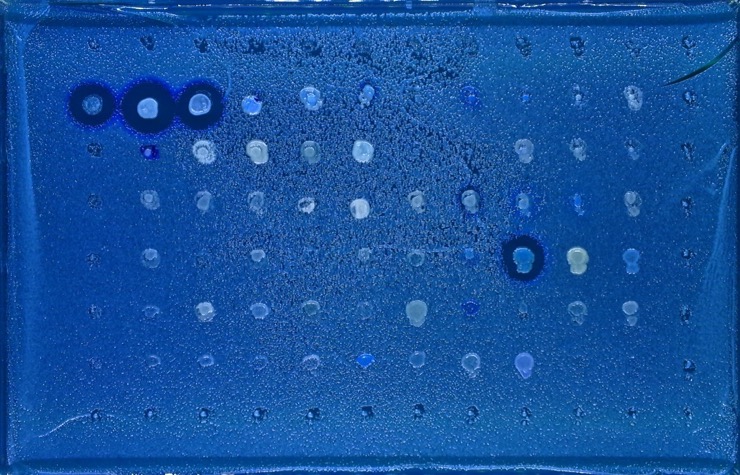


Lawn: FY4


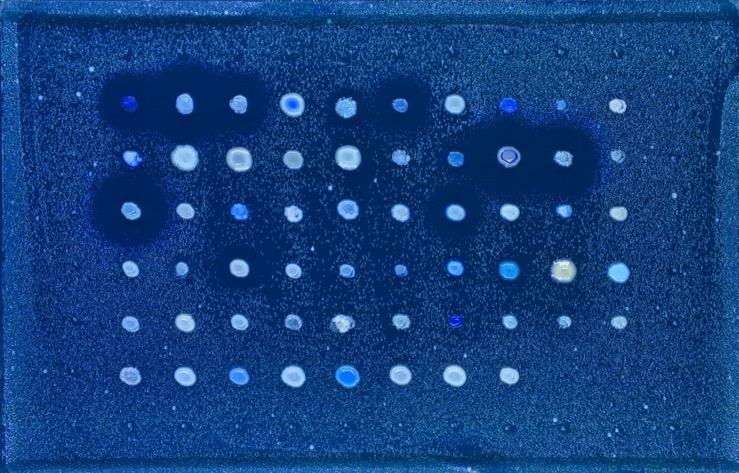


Lawn: K12


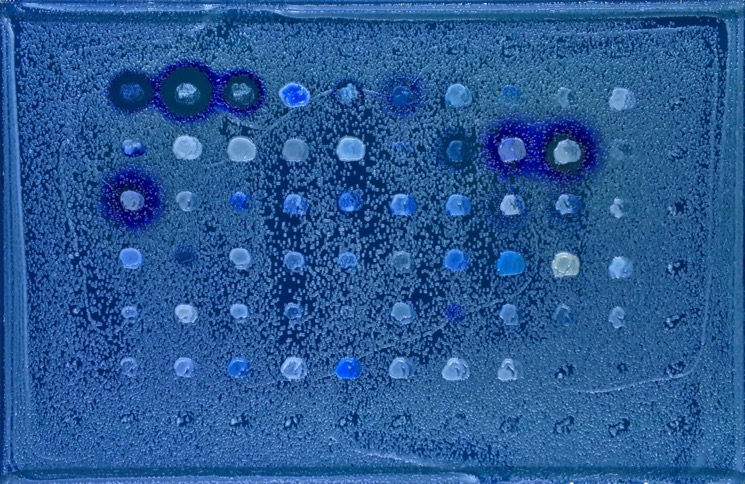


Lawn: BY4741


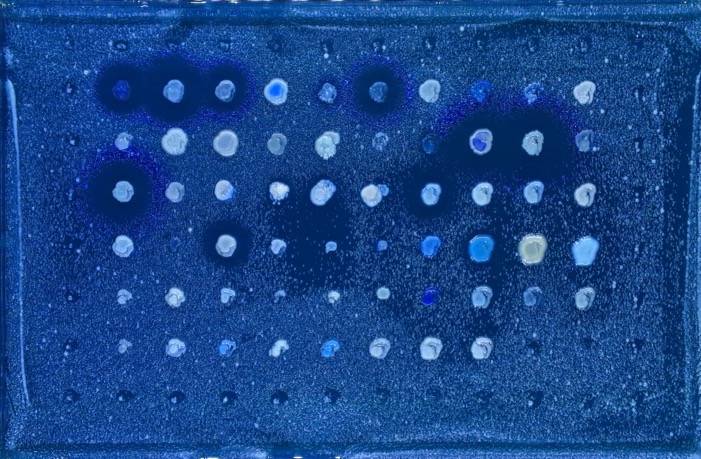


Lawn: DBVPG 6765


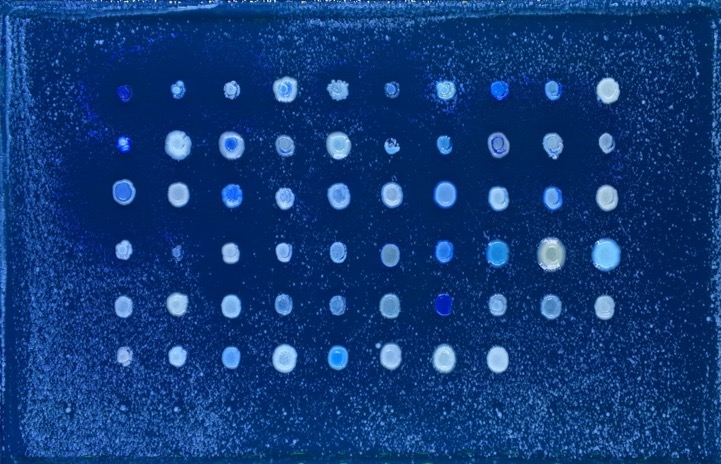


Lawn: CYC 1102


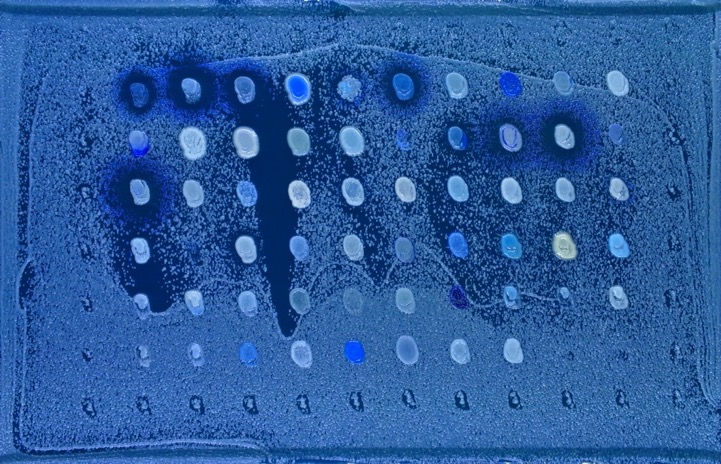


Lawn: YB-4237


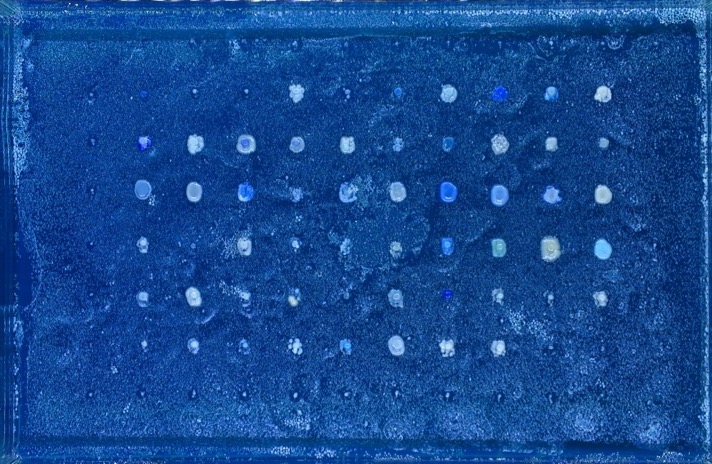


Lawn: CBS 432


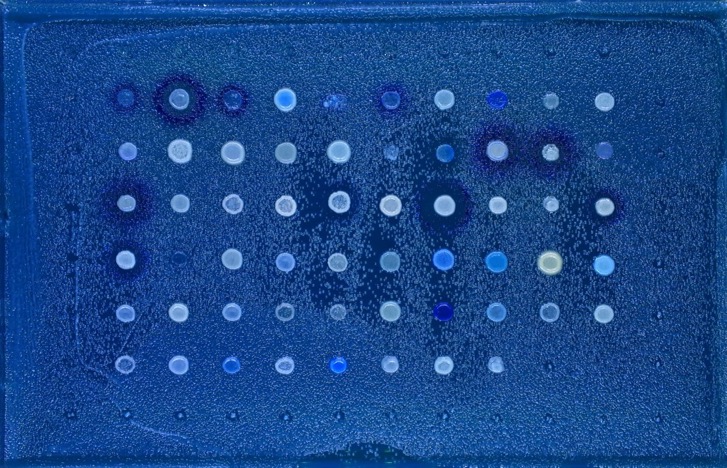


Lawn: A12C


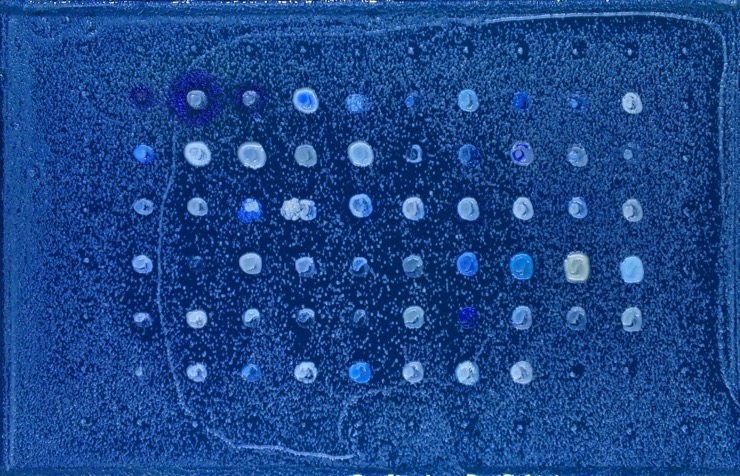


Lawn: NBRC 1815


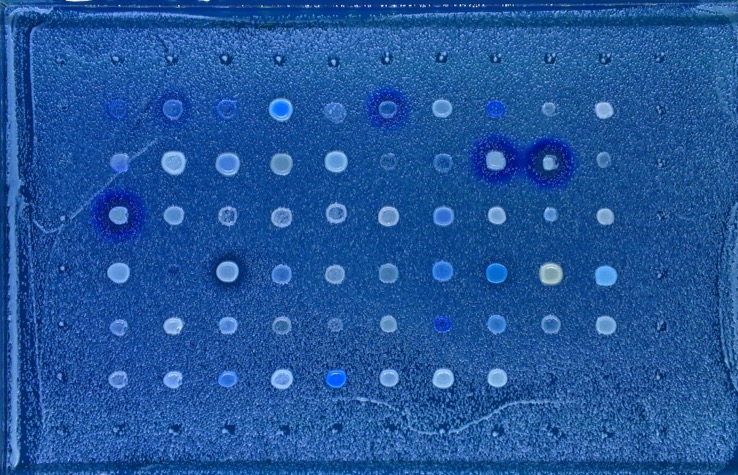


Lawn: SSS 211


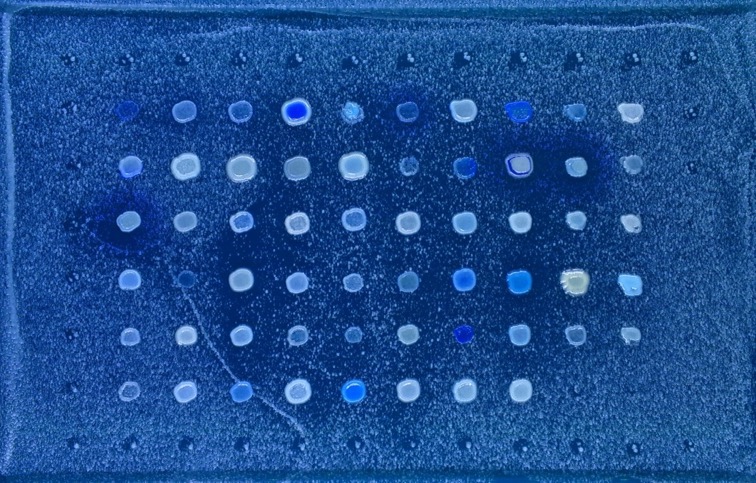


Lawn: NBRC 1802


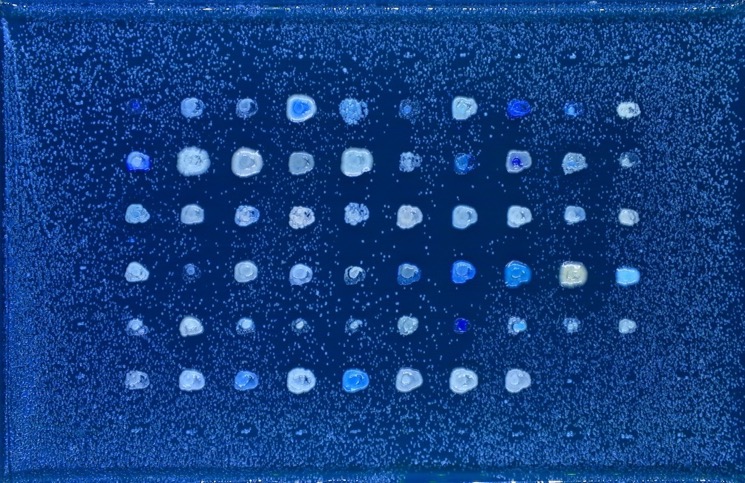


Lawn: CBS 7001


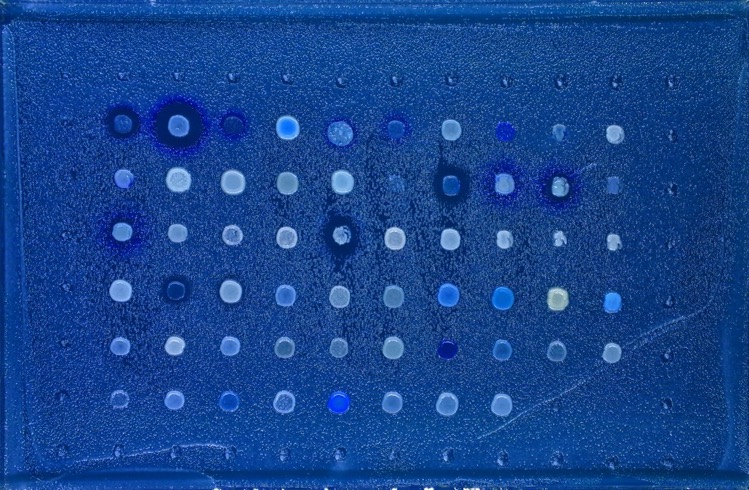


Lawn: SSS 104


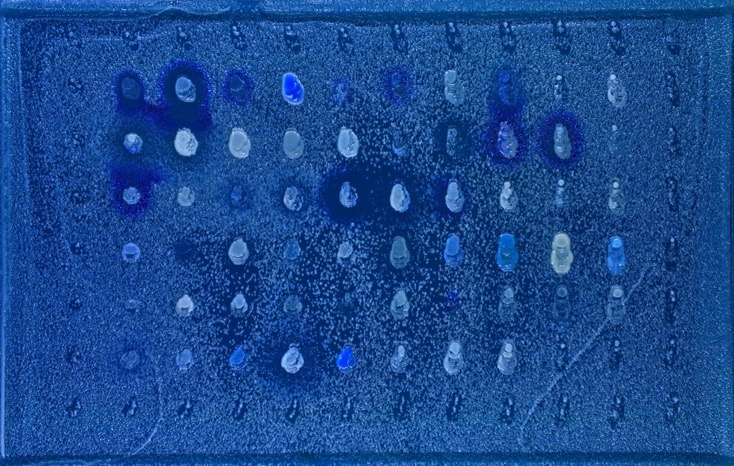


Lawn: Y-63711


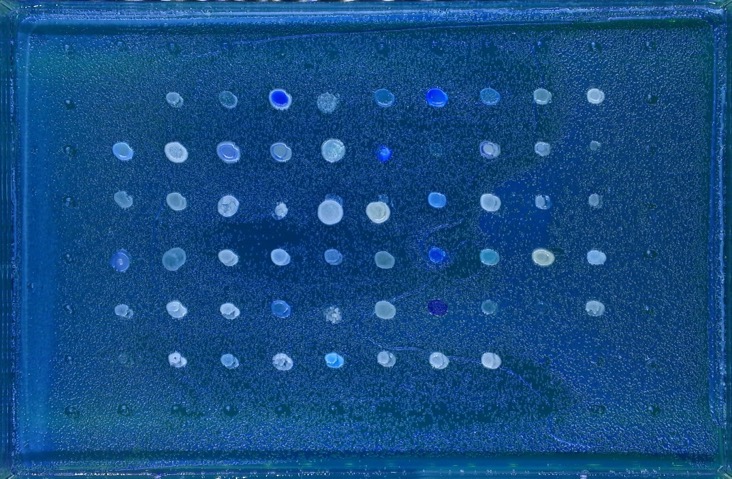


Lawn: Y-63716


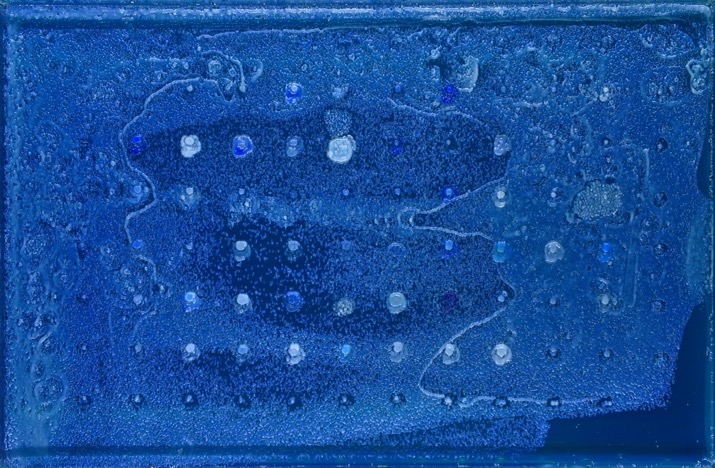


Lawn: YB-4565


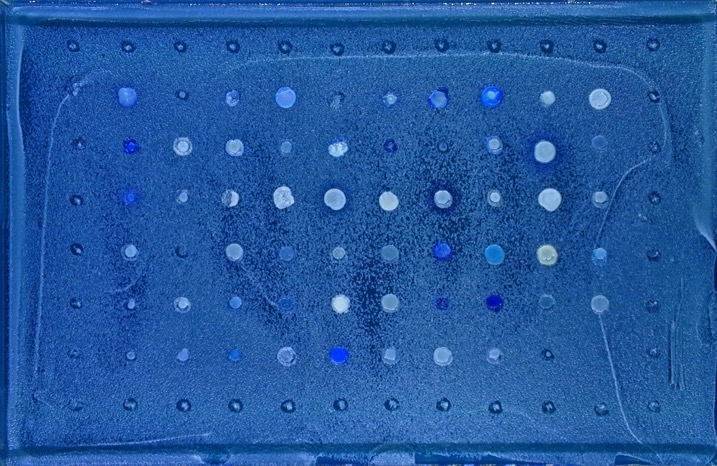


Lawn: 1116


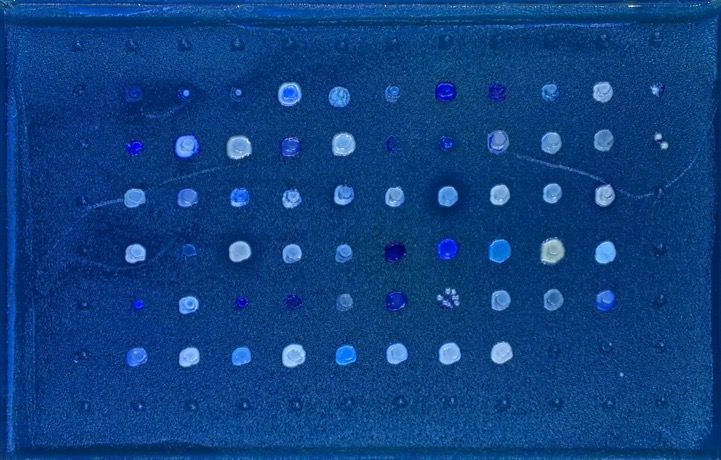


Lawn: Y-2429


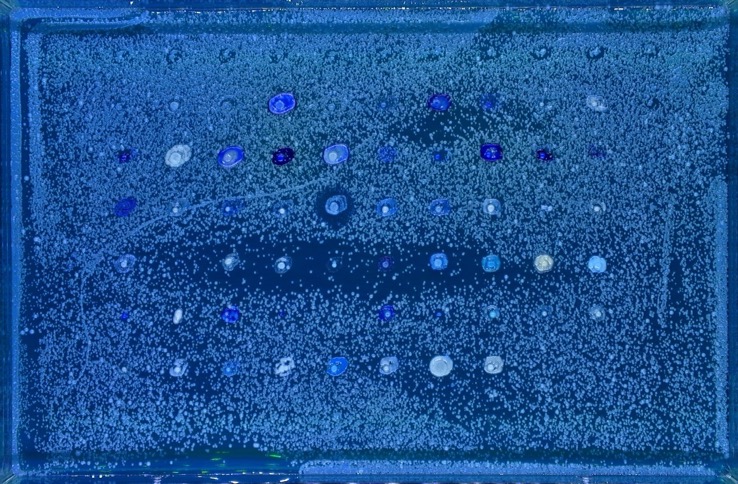


Lawn: DBVPG 6304


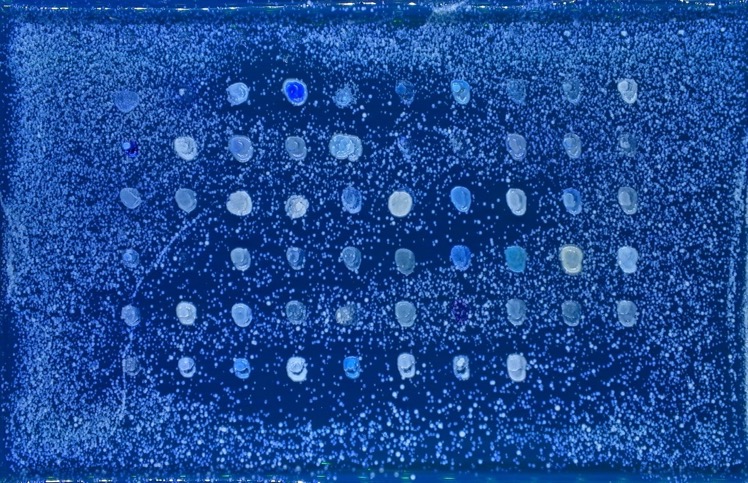


Lawn: Y-63717


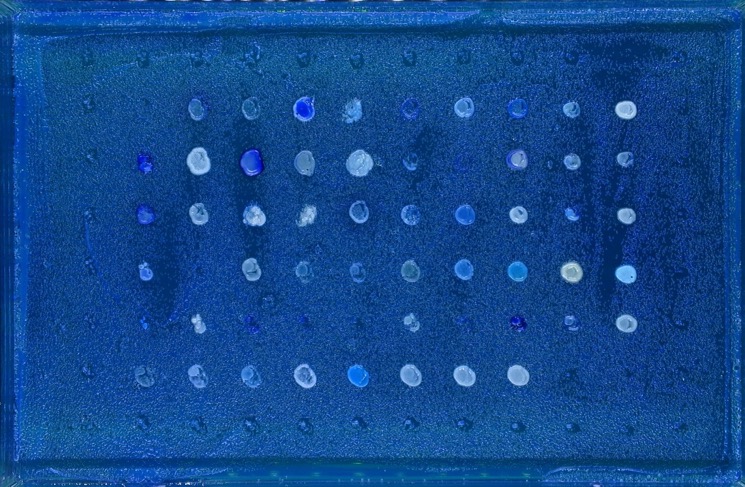


Lawn: Y8.5


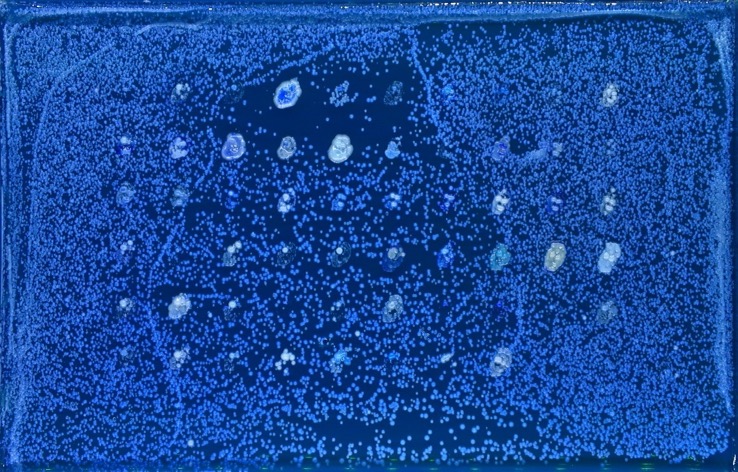


Lawn: Y-12602


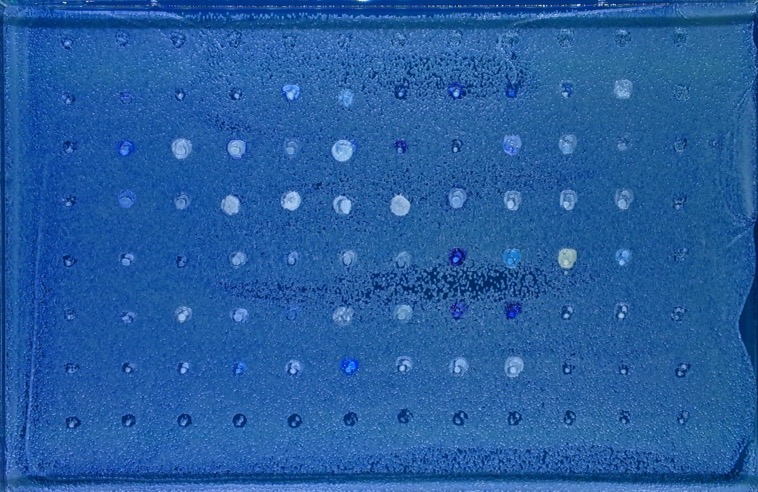

Supplement: S2 File — (DOCX) [file pgen.1009341.s017.docx]
