# Supplementary material for: The Species-Specific Acquisition and Diversification of a K1-like Family of Killer Toxins in Budding Yeasts of the Saccharomycotina
Source: PLoS Genet. 2021 Feb 4;17(2):e1009341. doi: 10.1371/journal.pgen.1009341 (PMC7888664; doi:10.1371/journal.pgen.1009341)
Supplement: S4 File — (PDF) [file pgen.1009341.s019.pdf]

Strain list

**Genus**	**Species**	**Strain**	**Source**
Saccharomyces	*cerevisiae*	BY4741	Rowley lab
Saccharomyces	*cerevisiae*	BHJ001	Rowley lab
Saccharomyces	*paradoxus*	Y-63717	NRRL(ARS)
Saccharomyces	*paradoxus*	A12C	Rowley lab
Saccharomyces	*cerevisiae*	DSM70459	DSMZ
Saccharomyces	*cerevisiae*	NCYC190	NCYC
Saccharomyces	*cerevisiae*	CYC1058	CYC
Saccharomyces	*cerevisiae*	CYC1113	CYC
Saccharomyces	*cerevisiae*	NCYC1001	NCYC
Saccharomyces	*paradoxus*	Y8.5	NCYC
Naumovozyma	*castelii*	NCYC_2898	NCYC
Naumovozyma	*dairenensis*	NCYC_777	NCYC
Kazachstania	*africana*	NCYC_2729	NCYC
Torulaspora	*delbrueckii*	Y-866	NRRL(ARS)
Tetrapisispora	*phaffii*	Y-8282	NRRL(ARS)
Pichia	*membranifaciens*	Y-2026	NRRL(ARS)
Pichia	*membranifaciens*	Y-6797	NRRL(ARS)
Pichia	*membranifaciens*	NCYC_333	NCYC
Pichia	*membranifaciens*	NCYC_2788	NCYC
Pichia	*membranifaciens*	P43C007	Okubara lab
Saccharomyces	*arboricolus*	Y-580	NRRL(ARS)
Saccharomyces	*arboricolus*	Y-670	NRRL(ARS)
Saccharomyces	*bayanus*	Y-846	NRRL(ARS)
Saccharomyces	*bayanus*	Y-969	NRRL(ARS)
Saccharomyces	*bayanus*	Y-972	NRRL(ARS)
Saccharomyces	*bayanus*	Y-1374	NRRL(ARS)
Saccharomyces	*bayanus*	Y-11845	NRRL(ARS)
Saccharomyces	*bayanus*	Y-12624	NRRL(ARS)
Saccharomyces	*bayanus*	Y-12646	NRRL(ARS)
Saccharomyces	*bayanus*	Y-12648	NRRL(ARS)
Saccharomyces	*bayanus*	Y-17034	NRRL(ARS)
Saccharomyces	*bayanus*	Y-27339	NRRL(ARS)
Saccharomyces	*bayanus*	Y-27470	NRRL(ARS)
Saccharomyces	*bayanus*	Y-48770	NRRL(ARS)
Saccharomyces	*bayanus*	Y-63707	NRRL(ARS)
Saccharomyces	*bayanus*	Y-63718	NRRL(ARS)
Saccharomyces	*cerevisiae*	YB-254	NRRL(ARS)
Saccharomyces	*cerevisiae*	YB-432	NRRL(ARS)
Saccharomyces	*cerevisiae*	Y-567	NRRL(ARS)
Saccharomyces	*cerevisiae*	Y-851	NRRL(ARS)
Saccharomyces	*cerevisiae*	Y-852	NRRL(ARS)
Saccharomyces	*cerevisiae*	Y-897	NRRL(ARS)
Saccharomyces	*cerevisiae*	Y-898	NRRL(ARS)
Saccharomyces	*cerevisiae*	YB-908	NRRL(ARS)
Saccharomyces	*cerevisiae*	Y-954	NRRL(ARS)

Saccharomyces	*cerevisiae*	Y-975	NRRL(ARS)
Saccharomyces	*cerevisiae*	Y-976	NRRL(ARS)
Saccharomyces	*cerevisiae*	Y-977	NRRL(ARS)
Saccharomyces	*cerevisiae*	Y-1018	NRRL(ARS)
Saccharomyces	*cerevisiae*	Y-1089	NRRL(ARS)
Saccharomyces	*cerevisiae*	Y-1285	NRRL(ARS)
Saccharomyces	*cerevisiae*	Y-1301	NRRL(ARS)
Saccharomyces	*cerevisiae*	Y-1370	NRRL(ARS)
Saccharomyces	*cerevisiae*	Y-1428	NRRL(ARS)
Saccharomyces	*cerevisiae*	Y-1429	NRRL(ARS)
Saccharomyces	*cerevisiae*	Y-1430	NRRL(ARS)
Saccharomyces	*cerevisiae*	Y-1436	NRRL(ARS)
Saccharomyces	*cerevisiae*	Y-1438	NRRL(ARS)
Saccharomyces	*cerevisiae*	Y-1536	NRRL(ARS)
Saccharomyces	*cerevisiae*	Y-1540	NRRL(ARS)
Saccharomyces	*cerevisiae*	YB-1773	NRRL(ARS)
Saccharomyces	*cerevisiae*	Y-1891	NRRL(ARS)
Saccharomyces	*cerevisiae*	Y-2044	NRRL(ARS)
Saccharomyces	*cerevisiae*	Y-2045	NRRL(ARS)
Saccharomyces	*cerevisiae*	Y-2046	NRRL(ARS)
Saccharomyces	*cerevisiae*	Y-2204	NRRL(ARS)
Saccharomyces	*cerevisiae*	Y-2205	NRRL(ARS)
Saccharomyces	*cerevisiae*	Y-2429	NRRL(ARS)
Saccharomyces	*cerevisiae*	Y-2430	NRRL(ARS)
Saccharomyces	*cerevisiae*	Y-2432	NRRL(ARS)
Saccharomyces	*cerevisiae*	Y-2434	NRRL(ARS)
Saccharomyces	*cerevisiae*	YB-4237	NRRL(ARS)
Saccharomyces	*cerevisiae*	YB-4255	NRRL(ARS)
Saccharomyces	*cerevisiae*	YB-4634	NRRL(ARS)
Saccharomyces	*cerevisiae*	YB-4635	NRRL(ARS)
Saccharomyces	*cerevisiae*	Y-5508	NRRL(ARS)
Saccharomyces	*cerevisiae*	Y-5509	NRRL(ARS)
Saccharomyces	*cerevisiae*	Y-5510	NRRL(ARS)
Saccharomyces	*cerevisiae*	Y-7327	NRRL(ARS)
Saccharomyces	*cerevisiae*	Y-7328	NRRL(ARS)
Saccharomyces	*cerevisiae*	Y-7567	NRRL(ARS)
Saccharomyces	*cerevisiae*	Y-10988	NRRL(ARS)
Saccharomyces	*cerevisiae*	Y-11875	NRRL(ARS)
Saccharomyces	*cerevisiae*	Y-12842	NRRL(ARS)
Saccharomyces	*cerevisiae*	Y-17009	NRRL(ARS)
Saccharomyces	*cerevisiae*	Y-17898	NRRL(ARS)
Saccharomyces	*cerevisiae*	Y-27105	NRRL(ARS)
Saccharomyces	*cerevisiae*	Y-27106	NRRL(ARS)
Saccharomyces	*cerevisiae*	Y-27437	NRRL(ARS)
Saccharomyces	*cerevisiae*	Y-27788	NRRL(ARS)
Saccharomyces	*cerevisiae*	Y-27796	NRRL(ARS)
Saccharomyces	*cerevisiae*	y-63703	NRRL(ARS)
Saccharomyces	*cerevisiae*	Y-63748	NRRL(ARS)

Saccharomyces	*cerevisiae*	Y-63749	NRRL(ARS)
Saccharomyces	*kudriavzevii*	Y-27340	NRRL(ARS)
Saccharomyces	*kudriavzevii*	Y-27341	NRRL(ARS)
Saccharomyces	*kudriavzevii*	Y-27342	NRRL(ARS)
Saccharomyces	*kudriavzevii*	Y-27471	NRRL(ARS)
Saccharomyces	*kudriavzevii*	Y-63704	NRRL(ARS)
Saccharomyces	*kudriavzevii*	Y-63705	NRRL(ARS)
Saccharomyces	*kudriavzevii*	Y-63706	NRRL(ARS)
Saccharomyces	*paradoxus*	Y-788	NRRL(ARS)
Saccharomyces	*paradoxus*	Y-863	NRRL(ARS)
Saccharomyces	*paradoxus*	Y-911	NRRL(ARS)
Saccharomyces	*paradoxus*	Y-1088	NRRL(ARS)
Saccharomyces	*paradoxus*	Y-1344	NRRL(ARS)
Saccharomyces	*paradoxus*	Y-1356	NRRL(ARS)
Saccharomyces	*paradoxus*	Y-1548	NRRL(ARS)
Saccharomyces	*paradoxus*	Y-1912	NRRL(ARS)
Saccharomyces	*paradoxus*	Y-2038	NRRL(ARS)
Saccharomyces	*paradoxus*	YB-2047	NRRL(ARS)
Saccharomyces	*paradoxus*	YB-4137	NRRL(ARS)
Saccharomyces	*paradoxus*	YB-4565	NRRL(ARS)
Saccharomyces	*paradoxus*	Y-5688	NRRL(ARS)
Saccharomyces	*paradoxus*	Y-6177	NRRL(ARS)
Saccharomyces	*paradoxus*	Y-6179	NRRL(ARS)
Saccharomyces	*paradoxus*	Y-11842	NRRL(ARS)
Saccharomyces	*paradoxus*	Y-12602	NRRL(ARS)
Saccharomyces	*paradoxus*	Y-17218	NRRL(ARS)
Saccharomyces	*paradoxus*	Y-17353	NRRL(ARS)
Saccharomyces	*paradoxus*	Y-63708	NRRL(ARS)
Saccharomyces	*paradoxus*	Y-63709	NRRL(ARS)
Saccharomyces	*paradoxus*	Y-63710	NRRL(ARS)
Saccharomyces	*paradoxus*	Y-63711	NRRL(ARS)
Saccharomyces	*paradoxus*	Y-63712	NRRL(ARS)
Saccharomyces	*paradoxus*	Y-63713	NRRL(ARS)
Saccharomyces	*paradoxus*	Y-63714	NRRL(ARS)
Saccharomyces	*paradoxus*	Y-63715	NRRL(ARS)
Saccharomyces	*paradoxus*	Y-63716	NRRL(ARS)

Primers

Name	Sequence (5'-3')	Notes
PRUI185	AACATTTTCGGTTTGTATTACTTCTTATTCTCTAAAAATGAGAAATAGTACC	K1L amplification for cloning
PRUI186	GTATCGTGATGACAGAGGCAGGGAGTGGGATCAAGCGCCAGTATCGCATTGGCTCC	"
PRUI054	CAACTGAAAACACTCCATCTGTTTCTTACC	KKT amplification from P. membranifaciens NCYC333
PRUI055	TTAACTCCCAGTATCACATTCAGTTTCGTATGG	"
PRUI052	TGCAAGGCTTGAAAAATGAAGTTAGC	KKT amplification from Naumovozyma dairenensis CBS 421 chr 7
PRUI053	CATTTGAAGGCCTATAGGGAAGAGG	"
PRUI056	TGACTAGAAACTCATGTCGCCAACC	KKT amplification from Kazachstania africana CBS2517 chr 1
PRUI057	AAAGTGAAATTTGCAAGATCATTAGTACCC	"
PRUI060	TGTTGAAGCTAAACAGTTTTAACAGAGTGG	KKT amplification from Kazachstania africana CBS2517 chr 12
PRUI061	GTCGAACAAGAAGGGAAAATTACGG	"
PRUI233	GACCAGCGGATAAACAGTATGTGC	KKT amplification from Tetrapisispora phaffii CBS 4417 chr 2
PRUI234	TTTTACAAAAGAAAATGCAAGCAAGC	"
PRUI235	AACAACCGATTCATTAGCAATAGGC	KKT amplification from Tetrapisispora phaffii CBS 4417 chr 11
PRUI236	AACGCCTTCTTATTTAAGCGTCTCG	"
PRUI237	AACGCCTTCTTATTTAAGCGTCTCG	KKT amplification from Naumovozyma castellii CBS 4309 chr 6
PRUI238	TGTGTGAACCATTCTTCAACGTACC	"
PRUI239	ACCATATTGGGGTTATTTTCGTTCG	KKT amplification from Naumovozyma dairenensis CBS 421 chr 8
PRUI240	TTGTTGAAGACATAAAAACGCATCG	"
PRUI241	AAAAACGCATATTGAAGTTGTTCTCG	KKT amplification from Naumovozyma dairenensis CBS 421 chr 3
PRUI242	GTGCCTTAATGAAACTTAGTGGTTGG	"
PRUI243	ATGAGAGTTGTTGGAATTTTTACTGTTTTGG	KKT amplification from Tetrapisispora phaffii CBS 4417 chr 1
PRUI244	CTAACTTCCTGTATCACATGAATCCC	"
PRUI245	TGAGAAATAGTACCTTCACCCTTAATTTGA	Amplify 5' region of M1L from Y-63717 (Figure S3 RXN1)
PRUI246	TTTGGCTCTGCACTTTACTCTATACTACG	"
PRUI247	AACTGGTAGTCCACGTCACTTACGG	Amplify 3' region of M1L from Y-63717 (Figure S3 RXN2)
PRUI248	TTGTTCAGTATTCAGGACCTATTGTCG	"
PRUI249	ACAGGTTGTGGAACAGTATTTGTGG	Amplify central poly(A) region of M1L from Y-63717 (Figure S3 RXN3)
PRUI250	GATGTCAGATATGCCAGCTTTTCC	"
AMC001	CCTATAGATATCTTAACAGC	Use for 5' RACE kit as GSP1 for top strand targeting
AMC002	TAACAGAATTAACTACTCAG	Use for 5' RACE kit as GSP1 for bottom strand targeting
prMDL071	GGACACCTGTGACGCTTGTA	KKT from Tetrapisispora phaffi CBS 4417 chromosome 2 in pUI111
prMDL072	TCACATGAATCCCAATAAGCA	"

prMDL073	TGTCGGATCAGTTAATGTAGCTT	KKT from Kazachstania africana CBS2517 chromosome 12 in pMDL118
prMDL074	CATTAAATTGAGCACGAGAGTTGTC	"
prMDL075	TCAATCAACCACCAATGTTATCC	KKT from Kazachstania africana CBS2517 chromosome 1 in pMDL117
prMDL076	CAAGTGTTCCGGCCAAATTAG	"
prMDL077	GCAGGTGCAAGAGGAAAAAG	KKT Naumovozyma dairenensis CBS 421 chromosome 8 in pUI112
prMDL078	GCACCTTTTTCCAGAAACGA	"
prMDL079	TGGTATGCTAAAGGGCCAAG	KKT Naumovozyma dairenensis CBS 421 chromosome 3 in pUI113
prMDL080	CTGCGAACGAAAGTGATTGA	"
prMDL081	CTGCACGCAGCTTATTGGTA	KKT fom Naumovozyma dairenensis CBS 421 chromosome 7 in pMDL115
prMDL082	CGCCAGAATCAGCACTACAA	"
prMDL083	AATACATGCAAGGCCCAGAC	KKT from Naumovozyma castellii CBS 4309 chromosome 6 in pUI114
prMDL084	TACCCAAACTGGCAAAGGTC	"
prMDL085	AGTGGTTATGGCGCTAATGG	KKT from P. membranifaciens NCYC333 in pMDL116
prMDL086	GACGTCCAGCTGCTACCTTC	"
prMDL087	GGAAACGGTTCATTTGCTGT	KKT from Tetrapisispora phaffi CBS 4417 chr 1 and 11 in pUI 109 and pUI110
prMDL088	TTCCAGCTTTCGCAAGTTTT	"
prMDL089	GGTAGCACAAAGCACCGAAT	KKT from Tetrapisispora phaffi CBS 4417 chr 1 and 11 in pUI 109 and pUI110
prMDL090	CGTAACTGAGCTTTTCCATGC	"
prMDL091	TATCGGGTCAGGTAGGAACG	K1L amplification from Saccharomyces paradoxus Y-63717 in pUI119
prMDL092	ATGGCATGGTTGACCTCTTC	"

Plasmid list

Name	Description	
pUI067	pDONR221 w/ K1L Y-63717	K1L
pUI109	pAG426-Gal w/ T. phaffi chromosome 2	KKT2
pUI110	pAG426-Gal w/ T. phaffi chromosome 11	KKT1
pUI111	pAG426-Gal w/ T. phaffi chromosome 1	KKT3
pUI112	pAG426-Gal w/ N. dair chromosome 8	KKT3
pUI113	pAG426-Gal w/ N. dair chromosome 3	KKT2
pUI114	pAG426-Gal w/ N. castelli chromosome 6	KKT1
pML115	pAG426-Gal w/ N. dair chromosome 7	KKT1
pML116	pAG426-Gal w/ P. memb	KKT1
pML117	pAG426-Gal w/ K. africana chromosome 1	KKT1
pML118	pAG426-Gal w/ K. africana chromosome 12	KKT2
pUI119	pAG426-Gal w/ S. paradoxus K1L	K1L
