# Supplementary material for: The Species-Specific Acquisition and Diversification of a K1-like Family of Killer Toxins in Budding Yeasts of the Saccharomycotina
Source: PLoS Genet. 2021 Feb 4;17(2):e1009341. doi: 10.1371/journal.pgen.1009341 (PMC7888664; doi:10.1371/journal.pgen.1009341)
Supplement: Attachment — Submitted filename: response to reviewers - PLoSgen_FINAL.docx [file pgen.1009341.s021.docx]

Nov 30^th^, 2020

Dear Editors,

Please find attached the revised manuscript with the revised title of “The Species-specific Acquisition and Diversification of a Family of K1-like Killer Toxins in Budding Yeasts of the Saccharomycotina”. We very much appreciated the reviewer’s comments and at their direction have added two new supplementary figures, two supplementary tables, and one data panel in the body of the manuscript.

We have also addressed all of the reviewer’s comments regarding the organization of the text and the interpretation of the data. Please find our detailed point-by-point response to every comment and suggestion raised by each of the three reviewers. For clarity we have indicated with a line number where each correction can be found, relative to the “clean” version of the manuscript.

**Responses can be found on the next page.**

Yours sincerely,

Paul A. Rowley

Office phone: (208) 885-4160
@DrPaulARowley
www.rowleylab.com

**Reviewer #1:** Fredericks et al explore the presence of killer phenotypes and killer toxins in a group of budding yeast species. They found additional strains that produce killer toxins, identified genes encoding killer toxins, and explored the evolutionary history of those genes. Overall, i think the paper makes a significant contribution to our understanding of an interesting topic. My biggest criticism is that I felt the novelty of the gene family was overstated. I appreciate the identified genes are greatly diverged from other K1 genes and have a ‘unique spectrum of activity’, but I wasn’t convinced it is helpful to call them a new family. Based on the title, I anticipated the family would be totally distinct from other identified killers. I think calling the family K1-like genes and including K1 is justified, but claiming they are a novel distinct family is not.

***Response:*** *On reflection,* ***w****e completely agree how this title could be misleading. As suggested by the reviewer, we have altered the title and several other sections of the manuscript to communicate that the toxins are part of a K1-like family.*

I have listed additional suggestions and points of confusion or concern below in an effort to help the authors improve the paper.

Line 153: Sequence reads were deposited to the NCBI Sequence Read Archive with the accession number: TBA.

***Response:*** *We have now uploaded the sequencing data and updated the accession numbers in the methods section of the manuscript.*

It would help increase the accessibility of the results to explain the significance of the pH and what’s happening with the methylene blue staining in the first paragraph of the results.

***Response:*** *We have expanded the description of the killer assay plates in the opening paragraph of the results.* Line 213 - “The first screen used eight yeasts from four different species to indicate toxin production on “killer assay medium”, which is a complex YPD medium buffered to pH 4.6, the optimal pH for most *Saccharomyces* killer toxins. Methylene blue was added to the medium as a redox indicator of cell death.”

Figure 1: labels for the indicator strains are missing. It is hard to match the ‘k’ labels on the left to the clusters on the right.

***Response:*** *We have added horizontal lines to help with the interpretation of the cluster graphs in the manuscript. We have also added the indicator strain names.*

Line 250: I had trouble following the numbers here. Were 19 strains tested? If so, how do you get 86% of those strains? 17 is 89% and 16 is 84%. There are 12 strains shown that have dsRNAs that could be cured of both the killing and the dsRNAs. I think i could follow this better if all the observed classes and the numbers in those classes were presented. Were the strains lacking the dsRNAs the only ones that could not be cured of the killer phenotype?

***Response:*** *We have greatly simplified this section and corrected the numbers accordingly:* Line 256 - “The production of killer toxins by yeasts is often associated with the presence of satellite dsRNAs that are maintained by totiviruses [25,45]. To determine whether satellite dsRNAs were responsible for the observed killer phenotypes, killer yeasts were treated with either cycloheximide, anisomycin, or incubated at elevated temperatures to select for the loss of killer toxin production [69,70]. The majority of killer yeast (12/19) lost their killer phenotype after exposure to chemical or thermal insult (Fig. 2A). The same treatments were unable to select for the loss of the killer phenotype in the remaining seven strains, which suggests that their killer toxins were genome-encoded (Fig. S1). To determine if the killer phenotype was correlated with the presence of dsRNAs, cellulose chromatography was used to selectively purify dsRNAs from the killer yeasts [32]. The analysis of extracted dsRNAs revealed that the loss of the killer phenotype was 100% correlated with the loss of satellite dsRNAs (Fig. 2A). Relative to the killer yeast strains, most of the isogenic cured strains exhibited an elevated copy number of totivirus dsRNAs. This phenomenon has been previously observed and is attributed to the fitness cost of parasitism by satellite dsRNAs [71].”

Line 253: “The same treatments were unable to select for the loss of the killer phenotype in five representative strains that lacked dsRNA satellites (Y-1344, Y-2046, YB-432, Y-1891, and Y-27788).” I couldn’t find the data supporting that statement.

***Response:*** *We now reference an amended figure S1 so that the reader can see the results from our efforts to cure these strains of the killer phenotype using cycloheximide as a table. Pictures of these negative data were not recorded by the students that performed the work.*

Line 255: “Relative to the wild type strains, most of the cured strains exhibited an elevated copy number of totivirus dsRNAs.” Was copy number formally assayed? If so those quantifications should be presented. Are there additional standard that would allow comparing band intensities between gels?

***Response:*** *The assessment of increased copy number was based on a qualitative assessment of the band intensities of the agarose gel. We now add a statement that clarifies that the same number of cells were used for each extraction.* Line 266 - “Relative to the killer yeast strains, most of the isogenic cured strains exhibited an elevated copy number of totivirus dsRNAs when the same numbers of cells were used during dsRNA extraction.”

Figure 2: The text says that 19 strains were assayed, but the figure doesn’t present all of them. I found the additional strains in the gel in supplement, but i didn’t see additional data for the curing treatment. It would also be helpful to see a totivirus negative control in A and a nonkiller strain in C.

***Response:*** *19 strains were assayed (including a control strains expressing K1 and K74. The data is split between Figure S1 and 2. We presented those yeasts that could be cured of their killer phenotype in figure 2. Based on the reviewers comments we have now added one representative yeast that was unable to be cured to panel 2A and have streamlined and added more information about the lack of curing to Figure S1 and to the body of the manuscript (described above). We also now include two controls in Figure 1 panel C showing the lack of toxin production by a non-killer yeast and a cured killer yeast strain.*

In 2B, It is difficult to tell which pHs were quantified. Data points in addition to the lines would help. An additional table in the supplement might also help. I can’t distinguish the colors well enough to know which line corresponds to which strain.

***Response:*** *We have now redrawn figure 2B to separate out all of the pH data for clarity. We have also clarified in the legend that discreet pH values were tested in this assay (pH 4, 4.5, 5, 5.5).*

Line 277: “BLASTn analysis of de novo assembled contigs revealed that dsRNAs within strains CYC1058 and NCYC1001 encode canonical K2 toxins and NCYC190 a canonical K1 toxin (Fig. S2)” From this, i expected S2 to show an alignment showing sequence similarity, but it plots contig length and coverage scores, which are not good support for the statement that the sequences are of K1 and K2 toxins.

***Response:*** *We have added a new supplementary table (Table S1) showing the percentage coverage and percentage identity of the nucleotide and amino acid sequences of the K2 and K1 toxins identified in these strains.*

Line 290: The reference to Fig S3 should be to Figure 3B

***Response:*** *Agreed, we have replaced this.*

Line 294: Not clear why Figure S3 is referenced here.

***Response:*** *Agreed, we have removed this.*

Line 301: Not clear why Figure 2 is referenced here.

***Response:*** *Changed to figure 3B*

Figure S5: Legend says some genes are colored green, but i can only see red and black. There is also a typo “Brocken” in the legend.

***Response****: We have corrected the figure legend to now reads: “N. dairenensis KKT2* inserted into chromosome III and *N. castellii KKT3* inserted into chromosome II. Genes flanking *KKT* insertions are represented as black arrows and demonstrate synteny between related genomes. Single red triangles represent tRNA genes. Broken lines represent gaps in synteny. (B) 5’ UTR sequence from *KKT* genes and one pseudogene identified within *K. africana*.”

Line 369: “The acquisition of KKT genes in T. phaffii appears to have occurred on three separate occasions resulting in three distinct clades of genes that have significant sequence divergence from each other and other KKT genes.” That is one model to explain that pattern, but others are possible. I suggest rephrasing this as a possibility rather than as something that is proven by the data.

“Each clade appears to be composed of 2-3 closely related paralogs, suggesting recent gene duplication events of a single ancestral gene.” It is also possible the duplications were not so recent, but that there is gene conversion between family members.

***Response:*** *We have rewritten this section to be more descriptive as is appropriate for the results section:* Line 381 - “The tree topologies of the two phylogenetic models are in general agreement and show the distinct clustering of KKT killer toxins from each species (Fig. 4C and S7). Importantly, K1L is most closely related to the KKT1 proteins from *T. delbrueckii* and *P. membranifaciens*. The KKT proteins from *K. africana* form a monophyletic clade that contrasts KKT proteins from *T. phaffii* and *N. dairenensis* that appear to be polyphyletic. There also appears to be some support for a common ancestry of KKT proteins within the *Naumovozyma* yeasts.”

Positive selection: The number of differences between the compared genes is difficult to determine from Figure S7. I am inclined to suspect the peaks of dN/dS>1 are noise based on few differences, rather than convincing evidence of positive selection. It is not clear how big the sliding windows are or how many changes differentiate the pairs of genes. The key for the symbols on the genes is also missing in S7.

***Response:*** *We now include additional information about the window size used for scanning these KKT genes and have added a key for the symbols. We also tone down the language to represent the uncertainty of our analysis:* Line 395 - “A domain-resolution approach using a sliding window to calculate dN/dS supports the predominance of purifying selection with a few localized peaks of weak positive selection (dN/dS >1) in the α- and/or γ-domains (Fig. S8).”

Figure 5: The color code for A is not provided here. Is it the same as the earlier figure? In B) Is T. delb a negative control here showing no killing, or is the phenotype minimal killing? In [figure 5] C, I suggest showing the Gal and glucose control plate together in one figure.

***Response:*** *We have added a statement in the figure legend to indicate the key is the same as in Figure 1 “*(A) *KKT*-encoding yeasts were assayed for killer toxin production on killer assay agar plates seeded with indicator strains. Killer toxin production was judged by the presence of zones of growth inhibition or methylene blue staining (see the key in Fig. 1).” *We have also replaced the original image of T. delbrueckii when assayed for killer toxin production with a clearer image in Figure 5B and added the dextrose and galactose data together in panel 5C.*

Line 400: “However, Y-2026 was still able to produce killer toxins, suggesting the production of other antifungal molecules by P. membranifaciens.” It also would not be unprecedented that the mutant can still sometimes generate a full length product. Sometimes mutations that should lead to premature truncations can still make some full length protein.

***Response:*** *We have included this suggestion:* Line 419 - “However, *P. membranifaciens* Y-2026 was still able to express killer toxins, suggesting the production of a defective KKT killer toxin or other antifungal molecules”

For the KKT genes in genomes, it would be nice to test if they are transcribed under the conditions in which you observed killing. As the authors point out, it is possible other genes are causing the killer phenotypes. Perhaps RNAseq data exists for these species? If not, perhaps do some RT-PCR? This will not prove the genes cause the killer phenotypes, but it could add further support for that notion.

***Response:*** *RT-PCR of 6 species that encode full length K1L genes was performed to detect transcripts produced by full-length KKT genes. A new panel has been added to Figure 5.*

**Reviewer #2:** Review of, “The Species-specific Acquisition and Diversification of a Novel Family of Killer Toxins
in Budding Yeasts of the Saccharomycotina” by Fredericks et al.
This is an extensive characterization of a large group of ‘killer’ strains of various yeasts, including many determined by dsRNAs and another group determined by chromosomal genes. The completeness of the work and the careful exploring of many aspects of the biology and evolution of these viruses and chromosomal genes makes a very interesting story, and the the work is of quite high quality. I have only a few suggestions for minor modifications.

A. The authors state that the killer genes are “a diverse arsenal of killer toxins for use in niche competition.” Also, a section of the Discussion, “The Benefits of Killer Toxin Acquisition” includes this view. This is certainly a widespread view of the killer systems, but it seems to be contradicted by the low incidence of toxin-producing or even toxin-resistant strains among wild isolates. It has been suggested, originally by Meinardt (?spelling) for the K. lactis DNA plasmid-based killer system, that the toxin is there to prevent the cell from losing the plasmid (or virus in this case), rather a more subversive role of the dsRNAs than the idea of helping the yeast host to conquer the niche world. The “9-10%” frequency of killer toxins is not typical of past studies, some of which have only been 1 or 2 % of isolates. I would suggest that the authors discuss this alternative view as well.

***Response****: We agree with the reviewer’s suggestion to balance our discussion of the benefits of killer toxin acquisition based on previous studies. We have renamed the discussion section as* “The Potential Benefits of Killer Toxin Acquisition” *and have highlighted previous studies that indicate that killer yeasts are a minority of strains and killer toxin resistance is relatively common and can be easily induced under laboratory conditions.* Line 524 - “However, competition between different killer yeasts likely selects for locally adapted populations that are immune to the predominant killer toxins in a specific environmental niche [6]. The laboratory evolution of killer yeast populations has also shown that the selective pressure of killer toxin exposure increases the prevalence of killer toxin resistance [112]. High levels of killer toxin resistance perhaps account for the low prevalence of killer yeasts within natural yeast populations and that killer toxin production might not always be advantageous.”

B. Line 468: It is suggested, but not quite clear to me whether you are saying that T. delbrueckii has RNAi and yet can still be infected with dsRNA viruses. If so, please include the fact that T. delbrueckii has an RNAi system.

***Response:*** *The text in the manuscript has been clarified:* Line 498 - “However, *T. delbrueckii* can support the replication of the putative totivirus TdV-LAbarr and an associated satellite [27], which suggests that this strain has lost its RNAi machinery or the totivirus has the ability to evade or disrupt RNAi similar to other mycoviruses [101–103]. The apparent invasion of these yeasts by viruses could enable the acquisition of killer toxins encoded upon satellite dsRNAs.”

C. Fig S4: I found the apparent conservation of the architecture of the M ‘putative viral particle binding sites’ quite remarkable, considering the evolutionary distance of the M dsRNAs. I assume this is a result of the ‘helper’ L-A Totiviruses, particularly their Pol domains that bind the site, being more closely related. The authors might want to comment on this aspect.

***Response:*** *We appreciate the reviewer’s particular interest in these results. We have expanded our discussion of these data in the manuscript:* Line 492 - “The apparent structural similarities of the satellite dsRNA-encoded *VBS* elements suggest that, despite the evolutionary distance between SpV-M1L and SpV-M45, each has evolved a similar mechanism to enable the hijack of the replication and packaging machinery from the L-A-45 totivirus.”

**Reviewer #3:** This manuscript describes the diversity of newly discovered K1-like (K1L) toxin-producing genetic elements found throughout the Saccharomycotina. Killer toxins are secreted by fungal cells and kill sensitive fungal cells, often of the same species. The toxin K1, which is expressed on a cytoplasmic dsRNA virus in S. cerevisiae, has been studied for decades, and a few other toxins have been discovered in S. cerevisiae and other fungi. But the diversity and evolution of these toxins are poorly studied, and this manuscript describes observations that give a window onto how toxin-coding genetic elements may evolve within and among fungal groups.

The authors discovered the K1L virus as part of a screen of S. cerevisiae and its sister species S. paradoxus. They found diverse combinations of target yeasts killed by toxin-producing Saccharomyces and diverse optimal pH values for toxins. After dsRNA sequencing, most toxins and associated toxin-coding genetic elements were previously known, but K1L, from a S. paradoxus strain, was previously undescribed (although weakly homologous to K1). A BLAST search found K1L homologs on the nuclear genomes of six other yeast species. Some of these homologs were pseudogenes, but most of the homologs coding for ORFs, when cloned, were able to be expressed in S. cerevisiae and produce toxins capable of killing sensitive yeasts. These newly discovered sequences were found on either nuclear or cytoplasmic nucleic acids, were polyphyletic, and when located on chromosomes were found in diverse and non-syntenic genomic locations (although most often in subtelomeric chromosomal regions). This diversity was taken as evidence for repeated horizontal gene transfer of toxin-coding genetic elements.

While researchers have previously found diverse toxin-coding genetic elements (both in terms of phylogeny and type of nucleic acid), and toxin genes are frequently transmitted horizontally, especially in prokaryotes, to my knowledge the result that the same toxin is likely horizontally transmitted and also coded in different ways among eukaryotic species is new. I also appreciated the new information presented on the general diversity natural history of killer toxins; while K1 is a model system in S. cerevisiae cell biology, the diversity and evolution of killer toxins among fungi is generally poorly understood. The results in this manuscript are surprising because the well-studied dsRNA viruses encoding many Saccharomyces toxins are thought to be only vertically transmitted. While the study doesn’t identify a mechanism of horizontal transmission (there are some possibilities suggested in the discussion), it does produce convincing evidence that this horizontal transmission not only happens at least sometimes, but also that conversion of viral RNA to genomic DNA or vice-versa must be a part of the process. I think the information presented in this manuscript is interesting and new, and should be published in PLoS Genetics. This manuscript is a strong contribution to our understanding of horizontal gene transfer among eukaryotes, and is also a considerable contribution to yeast researchers’ understanding of killer toxin systems.

I have a few minor suggestions for improvements to the manuscript, mostly in terms of manuscript organization and how information is presented.

1) My biggest criticism of this manuscript regards its organization: the main conclusion, that these new K1L toxins are widespread throughout the Saccharomycotina and have a pattern consistent with horizontal transfer of DNA and RNA elements, is buried in the manuscript and minimized in the abstract. The one exception is that these results are prominently presented in the abstract. I understand the need to report results in chronological order, but this strategy doesn’t effectively communicate the relative importance of results. This problem can be resolved with a rewording of the abstract and a refocusing of the introduction. Additionally, would it be possible to better integrate the two “stories” in this manuscript (that killer phenotypes within Saccharomyces are diverse and that K1L shows up in diverse places)?

***Response****: The lack of direct evidence of horizontal gene transfer informed our decision to tone down the significance of or findings. That being said, we have made several edits that we hope the reviewer thinks better present the significance of our findings:*

1. *We have reordered the abstract so that the last three sentences are focused on horizontal gene transfer of killer toxins.*
2. *The first paragraph of the introduction has been edited to add more weight to horizontal gene transfer: “*Many different species of fungi have been observed to produce proteinaceous killer toxins that inhibit the growth of competing fungal species [1–10]. The killer phenotype was reported in the budding yeast *Saccharomyces cerevisiae* in 1962, when Bevan *et al*. observed that spent culture medium had antifungal properties [11]. The potential future application of killer toxins as novel fungicides has led to the discovery of many different killer yeasts with varying specificities and toxicities [12,13]. In the *Saccharomyces* yeasts, including commonly used laboratory strains, it is estimated that 3-10% are able to produce killer toxins [7–10]. Despite the number of known killer yeasts that have been identified, a complete understanding of the diversity of killer toxins and their evolutionary history is lacking, even within the model yeast *S. cerevisiae*. The horizontal transfer of genetic material between different species of yeast is well appreciated [14–16] and the acquisition of killer toxins can be associated with the horizontal transfer of DNAs between related species that occupy similar ecological niches [17,18]. There is also evidence to support the sporadic genomic capture of killer toxins that are encoded by DNAs and RNAs that are derived from plasmids, viruses, and satellites [19,20]. These examples suggest that mobilization of killer toxins can occur by different mechanisms and that extrachromosomal elements play a role in gene flow between species.”
3. *To prepare the reader for the two sections of the manuscript we have altered the final paragraph of the introduction in the manuscript: “*This manuscript describes two novel findings related to killer toxin biology. The first is the identification of a novel killer toxin encoded by a satellite dsRNA in *S. paradoxus* by screening a large collection of *Saccharomyces* yeasts. This killer toxin has low sequence identity to the canonical K1 toxin produced by *S. cerevisiae* but has a similar secondary structure and domain organization*.* Due to its relatedness to K1, we have named this new killer toxin K1-like (K1L). The second major finding is that this is the first example of a dsRNA-encoded killer toxin from *Saccharomyces* yeasts that has significant homology to DNA-encoded killer toxins that we have named “K1 killer toxin-like” (*KKT*) within other species of the Saccharomycotina. These proteins represent a family of K1-like killer toxins that are both diverse and show signs of rapid protein evolution. This work provides insights into the expansion and horizontal transfer of killer toxin genes in yeasts, whether they are encoded by DNAs or mobilized and replicated as dsRNAs by viruses.”

2) It was sometimes difficult to keep track of the various strains discussed in this manuscript, especially in the Saccharomyces screens early in the manuscript. Would it be possible to give more information about why these particular strains are presented/how they were chosen? Indicating categories of strains on the figures would also help.

***Response****: We have simplified the inclusion of strain names used by only referring to the killer toxins that the control strains produce in the text of the manuscript. We instead now highlight them with the one non-killer yeast in Figure 1 (as suggested by the reviewer). The legend in figure 1 has been updated to indicate that “Y” and “YB” designations represent yeasts from the USDA Agricultural Research Service (ARS) culture collection. The error in the figure legend has been corrected to reflect the correct number of strains that we screened for killer toxin susceptibility.*

Additionally, in line 106, the manuscript says that 45 indicator lawn strains were used, but in Figure 1 it says 47. Did I miss the missing two somewhere? This paragraph might benefit from being rewritten to help readers keep track of strains.

***Response****: We have reformatted Figure 1 and have included more lawn strains. The number of strains (46) is now consistent within the text of the manuscript.*

3) The text indicates amino acid identity between K1 and K1L, and among K1L and newly discovered K1L-homologous nuclear sequences. But Figure 4a gives e-value and aligned length. I was interested in a plot that shows the aa identities mentioned in the text; could one be included, perhaps as an additional supplementary figure?

***Response****: We now include a supplementary table S2 that shows the percentage identity between K1 and K1L and against all other homologs. We have done this analysis using both full length killer toxins and the alpha domain alone.*

Other comments:
Lines 228-229: Could which known killers be indicated?

***Response****: Edited:* Line 240 -“The remaining killer yeasts were identified to have weaker inhibitory activities with no clustering with K1, K28, or K74 expressing killer yeasts. 

Lines 369-371: This statement might need to be weakened a little bit because the bootstrap values on the tree in Figure 4C are very low for some of these clades.

***Response****: We have amended this section:* Line 381 - “The tree topologies of the two phylogenetic models are in general agreement and show the distinct clustering of KKT killer toxins from each species (Fig. 4C and S7). Importantly, K1L is most closely related to the KKT1 proteins from *T. delbrueckii* and *P. membranifaciens*. The KKT proteins from *K. africana* form a monophyletic clade that contrasts KKT proteins from *T. phaffii* and *N. dairenensis* that appear to be polyphyletic. There also appears to be some support for a common ancestry of KKT proteins within the *Naumovozyma* yeasts.”

Lines 444-445: I’m not sure I quite understood what was meant by “independent origins”. Independent HGT events? Something else?

***Response****: We have rewritten this sentence:* Line 469 - “*KKT* genes have most likely been acquired by horizontal gene transfer because of their sporadic distribution and lack of common ancestry in closely related yeast species. Moreover, the lack of relatedness of *KKT* genes in these species suggests that they were acquired independently.”

Line 457: Yeasts in general or Saccharomyces in particular?

***Response****: We meant to refer to Saccharomyces yeasts:* Line 483 - “In particular for *Saccharomyces* yeasts, mating and hybridization between different species has been observed, and is a mechanism for gene introgression, as well as for the acquisition of retrotransposons and plasmids.”

Lines 476-478: This sentence was difficult to understand. Could it be reworded?

***Response****: We have clarified this sentence:* Line 506 - *“*The ancestral *KKT* gene in *K. africana* could have been captured directly from dsRNAs as all extant paralogs encode a 5’UTR “GAAAAA” motif, which is characteristic of satellite dsRNAs.”

Figure 2: It doesn’t look like this figure represents all of the killers identified in Figure 1. Why not/why were these chosen for the figure?

***Response****: We focused on presenting only those strains that had dsRNA satellites that could be cured of their dsRNAs. However, we now include one representative strain that represents a genome-encoded killer toxin in this figure as a reference. The rest are placed in Figure S1. We have also greatly simplified the accompanying text in the body of the manuscript:* Line 255 - “The production of killer toxins by yeasts is often associated with the presence of satellite dsRNAs that are maintained by totiviruses [25,45]. To determine whether satellite dsRNAs were responsible for the observed killer phenotypes, killer yeasts were treated with either cycloheximide, anisomycin, or incubated at elevated temperatures to select for the loss of killer toxin production [69,70]. The majority of killer yeast (12/19) lost their killer phenotype after exposure to chemical or thermal insult (Fig. 2A). The same treatments were unable to select for the loss of the killer phenotype in the remaining seven strains, which suggests that their killer toxins were genome-encoded (Fig. S1). To determine if the killer phenotype was correlated with the presence of dsRNAs, cellulose chromatography was used to selectively purify dsRNAs from the killer yeasts [32]. The analysis of extracted dsRNAs revealed that the loss of the killer phenotype was 100% correlated with the loss of satellite dsRNAs (Fig. 2A). Relative to the killer yeast strains, most of the isogenic cured strains exhibited an elevated copy number of totivirus dsRNAs when the same numbers of cells were used during dsRNA extraction. This phenomenon has been previously observed and is attributed to the fitness cost of parasitism by satellite dsRNAs [71].

Figure S1: What does “n/a” indicate? An explanation of the DNA band would be helpful in this figure.

***Response****: We have streamlined this figure and cropped all of the lanes that are not discussed in the paper. We have also mentioned that the DNA band is likely mitochondrial DNA “*Agarose gel electrophoresis is used to show dsRNA in killer yeasts. Satellite dsRNAs are labeled as dsRNAs that are smaller than the associated totivirus dsRNAs. The high molecular weight DNA is predicted to be mitochondrial in origin.”

A comparison of non-Saccharomyces killer sequence homology to K1L vs. homology to K1 would be a welcome addition to the data presented in this manuscript.

***Response****: We now include a supplementary table S2 that shows the percentage identity between K1 and K1L and against all other homologs. We have done this analysis using both full length killer toxins and the alpha domain alone.*

It might be nice to include a species tree of the species investigated (and the species mentioned on lines 331-333) so readers can easily compare phylogenetic patterns between K1L sequences and host species.

***Response****: We now include a cladogram in figure S5 representing the relatedness of each of the species that encode KKT genes, as well as including S. cerevisiae* and *S. paradoxus*.

I found a few typos:
Line 17: space in the word “these”

***Response****:* *Corrected*

Lines 65-66: “in Saccharomyces yeasts” is repeated

***Response****:* *Corrected*

Line 167: I couldn’t find these primers in table S1. Was Table S4 meant?

***Response****:* *You are correct, we have corrected this error.*

Figure S5 legend: What is colored green in this figure (this might be a pdf conversion issue)? Also, “Broken” is misspelled

***Response****: We have corrected the figure legend to now read: “*Genes flanking *KKT* insertions are represented as black arrows and demonstrate synteny between related genomes.”

Figure S10 legend: Part A seems to be missing

***Response:*** *Based on another reviewer’s suggestion, we have moved the unlabeled panel A to Figure 5C.*

**Have all data underlying the figures and results presented in the manuscript been provided?**
Large-scale datasets should be made available via a public repository as described in the *PLOS Genetics* [data availability policy](https://urldefense.com/v3/__http:/journals.plos.org/plosgenetics/s/data-availability__;!!JYXjzlvb!3Vqt5QsuCzQQ21GOCtpj0UOkKF38Kd1BsKli5EBQQiyu5hZotLQb2s7UUBSS3n7Miw$), and numerical data that underlies graphs or summary statistics should be provided in spreadsheet form as supporting information.

Reviewer #1: **No:** I listed a few places in the comments to authors where i could not find data to support statements. The SRA number was also listed as TBA in the methods.

Reviewer #2: Yes

Reviewer #3: Yes

PLOS authors have the option to publish the peer review history of their article ([what does this mean?](https://urldefense.com/v3/__https:/journals.plos.org/plosgenetics/s/editorial-and-peer-review-process*loc-peer-review-history__;Iw!!JYXjzlvb!3Vqt5QsuCzQQ21GOCtpj0UOkKF38Kd1BsKli5EBQQiyu5hZotLQb2s7UUBT4lQfXbw$)). If published, this will include your full peer review and any attached files.


If you choose “no”, your identity will remain anonymous but your review may still be made public.


**Do you want your identity to be public for this peer review?** For information about this choice, including consent withdrawal, please see our [Privacy Policy](https://urldefense.com/v3/__https:/www.plos.org/privacy-policy__;!!JYXjzlvb!3Vqt5QsuCzQQ21GOCtpj0UOkKF38Kd1BsKli5EBQQiyu5hZotLQb2s7UUBQa3BDpnA$).

Reviewer #1: No

Reviewer #2: No

Reviewer #3: No

*In compliance with data protection regulations, you may request that we remove your personal registration details at any time.* [*(Remove my information/details)*](https://urldefense.com/v3/__https:/www.editorialmanager.com/pgenetics/login.asp?a=r__;!!JYXjzlvb!3Vqt5QsuCzQQ21GOCtpj0UOkKF38Kd1BsKli5EBQQiyu5hZotLQb2s7UUBRLpvC0Ig$)*. Please contact the publication office if you have any questions.*
