# Supplementary material for: The Species-Specific Acquisition and Diversification of a K1-like Family of Killer Toxins in Budding Yeasts of the Saccharomycotina
Source: PLoS Genet. 2021 Feb 4;17(2):e1009341. doi: 10.1371/journal.pgen.1009341 (PMC7888664; doi:10.1371/journal.pgen.1009341)
Supplement: Attachment — Submitted filename: response to reviewer Dec 21st.docx [file pgen.1009341.s022.docx]

December 21^st^, 2020

Dear Editor,

Please find attached our response to the comments of reviewer #1 from the second round of review of our manuscript entitled “The Species-specific Acquisition and Diversification of a Family of K1-like Killer Toxins in Budding Yeasts of the Saccharomycotina” (PGENETICS-D-20-01491R1). We would like to apologize for not addressing the initial comments in a satisfactory manner and hope that this next round of revisions will be satisfactory to enable the acceptance of the manuscript in PLoS Genetics.

Yours sincerely,

Paul A. Rowley
Assistant Professor

Department of Biological Sciences,
The University of Idaho,
132 Gibb Hall,
875 Perimeter Drive MS 3051,
Moscow, ID 83844-3051, USA.

**Comments to the Authors:**
All changes are presented in the accompanying manuscript file.

Reviewer #1: The paper is improved by the revisions, but there are still two claims that are not sufficiently supported by data. These should be removed.

First, starting the assay with the same number of cells is insufficient to claim that there is a higher copy number of the totivirus after curing in Figure 2. I can see that the bands are brighter in the figure, but the bands we are comparing are on different gels without a common standard. I would have more confidence if it was clear if the data were all part of the same prep, assayed in the same PCR, and then run on the same gel.

***Response:*** We have removed the claim on copy number from the manuscript due to the lack of rigorous quantification of the dsRNAs that were prepared for figure 2.


The second claim is that there is positive selection. I appreciate that the authors added the size of the sliding window in their analyses. The authors have not, however, presented the number of synonymous and nonsynonymous differences between the compared proteins. Without seeing those numbers, i remain concerned that the values above 1 are just noise (eg. a window with low dS).

***Response:*** We have now updated the figure S8 to show the dN and dS values and that the elevated dN/dS ratio is not only due to a drop in dS (*K. africana*), but also a concurrent rise in dN (particularly for *T. phaffi*, but also in *N. dairenensis*). We have also removed the direct reference to the genes rapid evolution in the abstract and further weakened the language in the manuscript to represent the uncertainly of this conclusion.

One minor point:
Lines 430-433 says "...suggesting production of a defective KKT killer toxin..." This was confusing because there is killing. Why does killing suggest a defective toxin?

***Response:*** We have changed the sentence to: *“However, P. membranifaciens Y-2026 was still able to express killer toxins, suggesting the production of other alternative antifungal molecules.”*
